# Supplementary material for: Unify learns cellular evolution with universal multimodal embeddings
Source: Nat Commun. 2026 Jul 31;17:9290. doi: 10.1038/s41467-026-76230-y (PMC13527131; doi:10.1038/s41467-026-76230-y)
Supplement: Supplementary file 1 [file 41467_2026_76230_MOESM19_ESM.pdf]

nature portfolio

Corresponding author(s): Manuel Aranda, Xin Gao

Last updated by author(s): Jul 13, 2026

Reporting Summary

Nature Portfolio wishes to improve the reproducibility of the work that we publish. This form provides structure for consistency and transparency in reporting. For further information on Nature Portfolio policies, see our Editorial Policies and the Editorial Policy Checklist.

Statistics

For all statistical analyses, confirm that the following items are present in the figure legend, table legend, main text, or Methods section.

n/a	Confirmed	
☐	☒	The exact sample size (*n*) for each experimental group/condition, given as a discrete number and unit of measurement
☐	☒	A statement on whether measurements were taken from distinct samples or whether the same sample was measured repeatedly
☐	☒	The statistical test(s) used AND whether they are one- or two-sided<br>*Only common tests should be described solely by name; describe more complex techniques in the Methods section.*
☒	☐	A description of all covariates tested
☐	☒	A description of any assumptions or corrections, such as tests of normality and adjustment for multiple comparisons
☐	☒	A full description of the statistical parameters including central tendency (e.g. means) or other basic estimates (e.g. regression coefficient) AND variation (e.g. standard deviation) or associated estimates of uncertainty (e.g. confidence intervals)
☐	☒	For null hypothesis testing, the test statistic (e.g. *F*, *t*, *r*) with confidence intervals, effect sizes, degrees of freedom and *P* value noted<br>*Give P values as exact values whenever suitable.*
☒	☐	For Bayesian analysis, information on the choice of priors and Markov chain Monte Carlo settings
☒	☐	For hierarchical and complex designs, identification of the appropriate level for tests and full reporting of outcomes
☒	☐	Estimates of effect sizes (e.g. Cohen's *d*, Pearson's *r*), indicating how they were calculated

Our web collection on statistics for biologists contains articles on many of the points above.

Software and code

Policy information about availability of computer code

Data collection	All data analyzed with this manuscript are publicly available. No additional software was used for the data collection process.
Data analysis	The Unify software was developed and is publicly available at GitHub (https://github.com/huawen-poppy/Unify). Other data analyses were performed using SATURN (https://github.com/snap-stanford/SATURN), SAMap (version 1.0.15), Seurat (version 4.3.0), Scanorama (version 1.7), scVI (version 0.19.0), scGen (version 2.1.0), fastMNN (version 1.23.1), Harmony (version 1.2.0), BBKNN (version 2.0.1), Scikit-learn (version 1.0.2), SciPy (version 1.7.3), scIB (version 1.1.7), CLUSTALW (https://www.genome.jp/tools-bin/clustalw), ESPript (https://espript.ibcp.fr/ESPript/ESPript/), clusterProfiler (version 4.10.1), PHYLIP (version 3.698), scPARM (https://github.com/jiang-q19/scPRAM), Scanpy (version 1.9.1), R (version 4.3.2)

For manuscripts utilizing custom algorithms or software that are central to the research but not yet described in published literature, software must be made available to editors and reviewers. We strongly encourage code deposition in a community repository (e.g. GitHub). See the Nature Portfolio guidelines for submitting code & software for further information.

Data

Policy information about availability of data

All manuscripts must include a data availability statement. This statement should provide the following information, where applicable:

- Accession codes, unique identifiers, or web links for publicly available datasets
- A description of any restrictions on data availability
- For clinical datasets or third party data, please ensure that the statement adheres to our policy

Datasets are collected from published studies, as described in Supplementary Data 2. Source Data are provided with this paper. The output results for the benchmark section, ablation section and cross-species perturbation prediction session are available from Supplementary Data 3, Supplementary Data 5-9 and Supplementary Data 11-12, respectively. The proteomic datasets usage information in this study is available in Supplementary Data 13.

Research involving human participants, their data, or biological material

Policy information about studies with human participants or human data. See also policy information about sex, gender (identity/presentation), and sexual orientation and race, ethnicity and racism.

Reporting on sex and gender	None.
Reporting on race, ethnicity, or other socially relevant groupings	None.
Population characteristics	None.
Recruitment	None.
Ethics oversight	None.

Note that full information on the approval of the study protocol must also be provided in the manuscript.

Field-specific reporting

Please select the one below that is the best fit for your research. If you are not sure, read the appropriate sections before making your selection.

[x] Life sciences [ ] Behavioural & social sciences [ ] Ecological, evolutionary & environmental sciences

For a reference copy of the document with all sections, see nature.com/documents/nr-reporting-summary-flat.pdf

Life sciences study design

All studies must disclose on these points even when the disclosure is negative.

Sample size	No statistical method was used to predetermine sample size, as this study is a computational benchmarking and method-development study based on existing publicly available single-cell datasets rather than prospectively collected experimental samples. Dataset and task sizes were determined by data availability and by predefined inclusion criteria, including the availability of cell-type annotations, sufficient numbers of cells for integration or perturbation prediction, and the presence of comparable biological contexts across species. To assess performance across a range of biological settings, we evaluated Unify on ten phylogenetically diverse species-pair integration tasks involving 19 datasets, applied Unify to macrogene analysis using seven datasets, evaluated cross-species perturbation prediction using six datasets across 15 transfer tasks, and constructed a cell-type tree using datasets from seven species. These datasets and tasks were selected to cover multiple phylogenetic distances, species, tissues or cell types, and perturbation contexts, providing sufficient diversity to evaluate model performance across the intended use cases. Exact dataset sizes, including numbers of cells and genes used in each analysis, are reported in the Methods and Supplementary Data.
Data exclusions	Standard filtering procedures were performed to exclude low-quality cells and low-quality genes.
Replication	This study did not involve newly generated experimental samples. Replication was assessed computationally by evaluating Unify across independent publicly available datasets and multiple analysis settings, including ten species-pair integration tasks involving 19 datasets, macrogene analysis using seven datasets, cross-species perturbation prediction using six datasets across 15 transfer tasks, and cell-type tree analysis using datasets from seven species. The reported results were reproducible using the described code, parameters and input datasets.
Randomization	Our study does not involve allocating samples to experimental groups.
Blinding	Our study does not involve group allocation that requires blinding.

Reporting for specific materials, systems and methods

We require information from authors about some types of materials, experimental systems and methods used in many studies. Here, indicate whether each material, system or method listed is relevant to your study. If you are not sure if a list item applies to your research, read the appropriate section before selecting a response.

Materials & experimental systems

n/a	Involved in the study
☒	☐ Antibodies
☒	☐ Eukaryotic cell lines
☒	☐ Palaeontology and archaeology
☒	☐ Animals and other organisms
☒	☐ Clinical data
☒	☐ Dual use research of concern
☒	☐ Plants

Methods

n/a	Involved in the study
☒	☐ ChIP-seq
☒	☐ Flow cytometry
☒	☐ MRI-based neuroimaging

Plants

Seed stocks	No plant used.
Novel plant genotypes	No plant used.
Authentication	No plant used.
