# Supplementary material for: Unify learns cellular evolution with universal multimodal embeddings
Source: Nat Commun. 2026 Jul 31;17:9290. doi: 10.1038/s41467-026-76230-y (PMC13527131; doi:10.1038/s41467-026-76230-y)
Supplement: Supplementary file 2 [file 41467_2026_76230_MOESM1_ESM.pdf]

Unify learns cellular evolution with universal multimodal embeddings

Zhong and Han *et al.*

Supplementary Note 1. Gene functional description

Functional annotations for each gene can be drawn from various sources—Gene Ontology (GO) terms, Kyoto Encyclopedia of Genes and Genomes (KEGG) pathways, Reactome pathways, or other curated descriptions. In this study, we uniformly adopted GO terms to represent gene function across all species.

For well–studied organisms (human and mouse), we retrieved the reviewed GO annotations from the UniProt database[1]. For other species, we obtained their GO annotation files directly through the Gene Ontology Consortium's download portal[2]. If GO annotations are unavailable or incomplete (in less-studied species), they can be inferred using orthology-based tools such as eggNOG-mapper[3, 4].

To ensure consistent and functionally meaningful gene representation across diverse species, we standardized annotations using Gene Ontology (GO) terms. Where possible, we prioritized high-confidence, manually reviewed entries (e.g., from UniProtKB/Swiss-Prot), minimizing annotation bias and maximizing cross-species comparability.

Supplementary Note 2. Methods used in the benchmarking analysis

SATURN

SATURN[5] is a weakly-supervised autoencoder that incorporates cell type information from each species individually. In combination with the protein embeddings derived from a pretrained protein language model, it is trained to reconstruct the raw count expression data using the negative log likelihood of a ZINB distribution.

SAMap

SAMap[6] is a method designed to integrate single-cell RNA-seq datasets across species by aligning cell types based on both gene expression and gene homology information. It extends the Self-Assembling Manifold (SAM) algorithm by incorporating cross-species gene-gene mappings to guide the integration process. SAMap constructs manifolds for each species dataset and iteratively aligns them by updating gene-gene and cell-cell correspondences, allowing it to detect conserved and species-specific cell types.

fastMNN

fastMNN (Fast Mutual Nearest Neighbors)[7] is a method for integrating single-cell RNA-seq datasets by identifying shared cell populations across batches. It works by detecting mutual nearest neighbors (MNNs) between batches, i.e. cells that are closest to each other in expression space despite coming from different datasets. These MNN pairs help estimate the batch effect, which is

then corrected by aligning the batches in a common low-dimensional space. fastMNN improves computational efficiency by applying dimensionality reduction early and processing batches sequentially. fastMNN for cross-species data integration is restricted to orthologous genes.

Harmony

Harmony[8] is an algorithm designed to integrate single-cell RNA-seq datasets by iteratively adjusting the generated cell embedding to align cells from different batches. It operates in a reduced dimensional space (typically PCA), where it groups cells into clusters based on similarity and then iteratively adjusts their positions to ensure each cluster contains a balanced mix of cells from different batches. By aligning shared cell types across datasets without relying on prior annotations, Harmony effectively removes technical variation and produces corrected embeddings suitable for downstream analysis. Harmony for cross-species data integration is restricted to orthologous genes.

scGen

scGen[9] is a deep learning-based method that uses variational autoencoders (VAEs) to model and predict gene expression responses in single-cell RNA-seq data. It learns a latent representation of cells that captures both biological variability and batch effects. During integration, scGen disentangles these factors and uses vector arithmetic in the latent space to simulate how cells from one condition or batch would appear in another. scGen for cross-species data integration is restricted to orthologous genes.

scVI

scVI (single-cell Variational Inference)[10] is a deep generative model that uses variational autoencoders (VAEs) to learn a probabilistic representation of single-cell RNA-seq data. It models gene expression counts using a negative binomial distribution, accounting for both biological variation and technical noise such as batch effects. By encoding cells into a shared latent space, scVI enables effective integration across different datasets or conditions. scVI for cross-species data integration is restricted to orthologous genes.

BBKNN

BBKNN (Batch Balanced KNN)[11] is a method for integrating single-cell RNA-seq datasets by correcting batch effects at the level of neighborhood graph construction. BBKNN builds a graph where each cell is connected to a specified number of nearest neighbors from each batch separately. This ensures that the local neighborhood of each cell is balanced across batches, reducing technical variation while maintaining the underlying biological structure. BBKNN operates on the principal components of the datasets. BBKNN for cross-species data integration is restricted to orthologous genes.

Scanorama

Scanorama[12] is a tool designed to integrate single-cell RNA-seq datasets from different batches or studies. The method starts by reducing the dimensionality of each dataset, identifying mutual nearest neighbors between pairs, and then correcting differences to bring shared cell types into alignment. Subsequently, Scanorama merges the data into a unified, corrected representation. It progressively merges datasets in a way that maintains biological variation while minimizing technical noise. Scanorama for cross-species data integration is restricted to orthologous genes.

Seurat V4

Seurat (version 4.3.0)[13] integrates single-cell datasets from different conditions, batches, technologies, or even species by identifying shared biological signals while correcting for technical differences. After dataset normalization, highly variable gene selection and dimension reduction, Seurat uses Canonical Correlation Analysis (CCA) to project datasets into a shared space and identifies anchors, which are pairs of biologically similar cells across datasets based on the mutual nearest neighbors. Seurat further uses the anchors to align the datasets into a common coordinate system. Seurat for cross-species data integration is restricted to orthologous genes.

Supplementary Note 3. Evaluation of batch correction and biological conservation

We treat species labels as "batches" and cell-type labels as "biological groups." After integration, we quantify batch removal and biological signal preservation for each method. For batch correction, we use the following sub-metrics: Adjusted Rand Index (ARI) batch, Adjusted Silhouette Width (ASW) batch, Normalized Mutual Information (NMI) batch, Local Inverse Simpson's Index (iLISI), kBET, graph connectivity, and Principal Component Regression. For bio-conservation, we use the following metrics: ARI cell type, ASW cell type, Cell-type LISI (cLISI), NMI cell type, highly variable gene (HVG) conservation and trajectory conservation. We run these evaluations through the scIB package[14].

Adjusted rand index (ARI)

ARI_{batch} measures the overall agreement between the inferred clusters and known species labels with adjustment for chance, while the $ARI_{celltype}$ measures the overall agreement between the inferred clusters and the known cell type labels with adjustment for chance.

$$ARI = \frac{\sum_{ij}\binom{n_{ij}}{2} - [\sum_i \binom{a_i}{2} \sum_j \binom{b_j}{2}]/\binom{n}{2}}{\frac{1}{2}\left[\sum_i \binom{a_i}{2} + \sum_j \binom{b_j}{2}\right] - [\sum_i \binom{a_i}{2} \sum_j \binom{b_j}{2}]/\binom{n}{2}} \qquad \textbf{(1)}$$

where n_{ij} is the number of cells in cluster i and batch/celltype j, $a_i = \sum_j n_{ij}$, $b_j = \sum_i n_{ij}$, and n is the total number of cells.

We used the Louvain clustering to compute the inferred clusters. The value ranges from 0 to 1. The higher value represents a higher match between the clusters. To ensure that higher values correspond to better batch mixing, we transform the ARI_{batch} score as follows:

$$ARI_{batch} = 1 - ARI_b \quad (2)$$

Adjusted silhouette width (ASW)

ASW computes the average silhouette s(i) over all cells. It measures how intermixed species/cell type labels are by comparing within-clusters and between-clusters distances, where the clusters refer to species or cell type for ASW batch and ASW cell type separately. The value ranges from -1 to 1. Higher values represent better-quality clusters.

$$s(i) = \frac{b(i)-a(i)}{max\{a(i),b(i)\}}, ASW = \frac{1}{n}\sum_{i=1}^{n} s(i) \quad (3)$$

where a(i) is the average distance of cell i to cells of its own cluster, and b(i) is the minimum average distance to any other clusters.

Graph local inverse simpson's index (LISI)

Graph LISI quantifies the diversity in a neighborhood, ranging from 1 (minimum diversity) to B (maximum diversity), where B is the number of batches or the cell types. To standardize interpretation, LISI scores are rescaled to the interval [0,1] following Luecken *et al.*[14]. We first calculate the median LISI across all the cells:

$$cLISI = median(f(x)), x \text{ is each cell} \quad (4)$$

$$iLISI = median(g(x)), x \text{ is each cell} \quad (5)$$

We further normalize the score between 0 and 1:

For cLISI:

$$f(x) = \frac{B-x}{B-1} \quad (6)$$

For iLISI:

$$g(x) = \frac{x-1}{B-1} \quad (7)$$

After normalization, higher value in cLISI and iLISI means perfect cell type separation and perfect species mixing.

Normalized mutual information (NMI)

NMI measures the similarity between two clusters. It ranges from 0 to 1. The higher value means better alignment between clusters. The NMI between cluster label C and class labels Y is defined as:

$$NMI(C, Y) = \frac{2 \times I(Y;C)}{H(C)+H(Y)} \quad \textbf{(8)}$$

where H(.) is the entropy and I(Y;C) is the mutual information between Y and C

$$I(Y; C) = \sum_{y \in Y} \sum_{c \in C} p(y, c) \log(\frac{p(y,c)}{p(y)p(c)}) \quad \textbf{(9)}$$

H(C) and H(Y) are the entropies

$$H(C) = -\sum_{c \in C} p(c) \log \text{ p(c)}, H(Y) = -\sum_{y \in Y} p(y) \log\ p(y) \quad \textbf{(10)}$$

p(c,y) is the joint probability of a cell being assigned to cluster c and label y, and p(c), p(y) are the marginal probabilities.

Similar to ARI, we use the Louvain clustering to compute the inferred clusters. For NMI batch, we reversed it by subtracting it from 1.

kNN batch effect test (kBET)

The kNN Batch-Effect Test (kBET) evaluates how well different batches are mixed in an integrated embedding. For each cell, kBET examines whether its local neighborhood's batch composition matches the expected global distribution. A high overall kBET value indicates successful batch mixing. We followed the code in scIB[14] to compute the kBET.

Principal component regression (PCR)

PCR quantifies how much of the variance in the top K principal components (PC) is explained by batch labels. For PC k, let $p_k = (p_{1k}, \dots, p_{nk})^T$ be the scores of all n cells along that component, and let B_i be the batch label of cell i. Fit the linear model

$$p_{ik} = \beta_0 + \sum_{b=1}^{B} \beta_b 1\{B_i = b\} + \varepsilon_{ik} \quad \textbf{(11)}$$

And compute

$$R_k^2 = 1 - \frac{\sum_{i=1}^{n}(p_{ik} - \hat{p}_{ik})^2}{\sum_{i=1}^{n}(p_{ik} - \bar{p}_k)^2} \quad \textbf{(12)}$$

where $\widehat{p_{ik}}$ are the fitted values and $\overline{p_k}$ is the mean of p_k.

Weight each R_k^2 by the variance explained λ_k of PC k, and normalize:

$$PCR_{batch} = \frac{\sum_{k=1}^{K} \lambda_k R_k^2}{\sum_{k=1}^{K} \lambda_k} \quad \textbf{(13)}$$

Here $\lambda_k = Var(p_{1k}, \dots, p_{nk})$ is the variance explained by PC k.

Graph connectivity

Graph connectivity evaluates how well cells sharing the same label (batch or cell type) remain interconnected in the integrated embedding's k-nearest-neighbor (kNN) graph. After building a kNN graph on all cells, one examines each label group by extracting the subgraph induced by that label's cells and measuring the size of its largest connected component relative to the total number of cells in the group. These proportions are then averaged over all labels to yield the overall connectivity score. High connectivity for batch labels indicates thorough mixing (cells from different batches interleave). This metric captures global structural coherence in the embedding and complements local measures (like silhouette or LISI) by assessing group-level continuity across the entire graph.

HVG conservation

HVG conservation measures the extent to which integration preserves key gene-level signals that drive cellular heterogeneity. Before and after applying an integration method, we independently identify the top set of highly variable genes within each species or batch. The conservation score is then defined as the proportion of overlapping genes between the pre- and post-integration HVG sets. A high HVG conservation score indicates that the integration process has maintained the most informative, biologically driven gene variation rather than smoothing it away. Conversely, a low score suggests that important gene-level differences, often linked to true cell type distinctions, have been attenuated by the integration. By focusing on gene-level variability, HVG conservation complements embedding-based metrics, ensuring that integration methods not only mix datasets effectively but also retain the critical molecular features underlying cell biology.

Trajectory conservation

Trajectory Conservation (TC) quantifies how well integration preserves dynamic, pseudotemporal relationships. We infer trajectories before and after integration using Scanpy's diffusion pseudotime (sc.tl.dpt)[15] and designate the most extreme cell in each cluster as the root. The Spearman rank correlation between pre- and post-integration pseudotime values, computed via the scIB pipeline, yields the TC score, which is then normalized to [0,1]. Higher TC values indicate better preservation of biological progression through the integration process.

Supplementary Note 4. Cell type tree construction

We built cross-species cell-type phylogenies following Jasmine *et al.*[16]. First, for both SATURN and Unify embeddings, we performed PCA and retained the top 20 components to reduce noise and computational load. To represent each of the 45 annotated cell types, we randomly sampled one cell per type, yielding a 45 × 20 data matrix.

Phylogenetic inference was carried out using PHYLIP's contml (v3.698) under a Brownian motion model of continuous character evolution. We enabled the continuous-data option (–C) and global rearrangements (–G), and randomized taxon order 100 times per run (–J 100), selecting the highest-likelihood tree each time. We repeated this process for 250 independent runs with 100 jitters per run, then midpoint-rooted the best-scoring tree for presentation.

To evaluate tree robustness, we calculate two repeatability measures:

(1) Technical repeatability (jumble score): the consistency of topology across random taxon-order perturbations.
(2) Biological repeatability (sc-jackknife score): the recovery rate of the original tree when resampling one cell per type (500 replicates).

Together, these analyses quantify both the stability and the biological fidelity of our integrated cell-type trees.

To assess the robustness of the cell-type representation used in the centroid-cell-based tree, we constructed an alternative meta-cell-based tree, in which each species-cell-type was represented by the mean embedding of all cells in the corresponding population. Using the same tree-construction procedure as for the centroid-cell-based tree, we then inferred the topology from these meta-cell representations (Supplementary Figure 38). The weighted Robinson–Foulds distance[17] between the two trees was 0.28, indicating strong topological similarity and suggesting that the inferred evolutionary relationships are robust to the choice of cell-type representation.

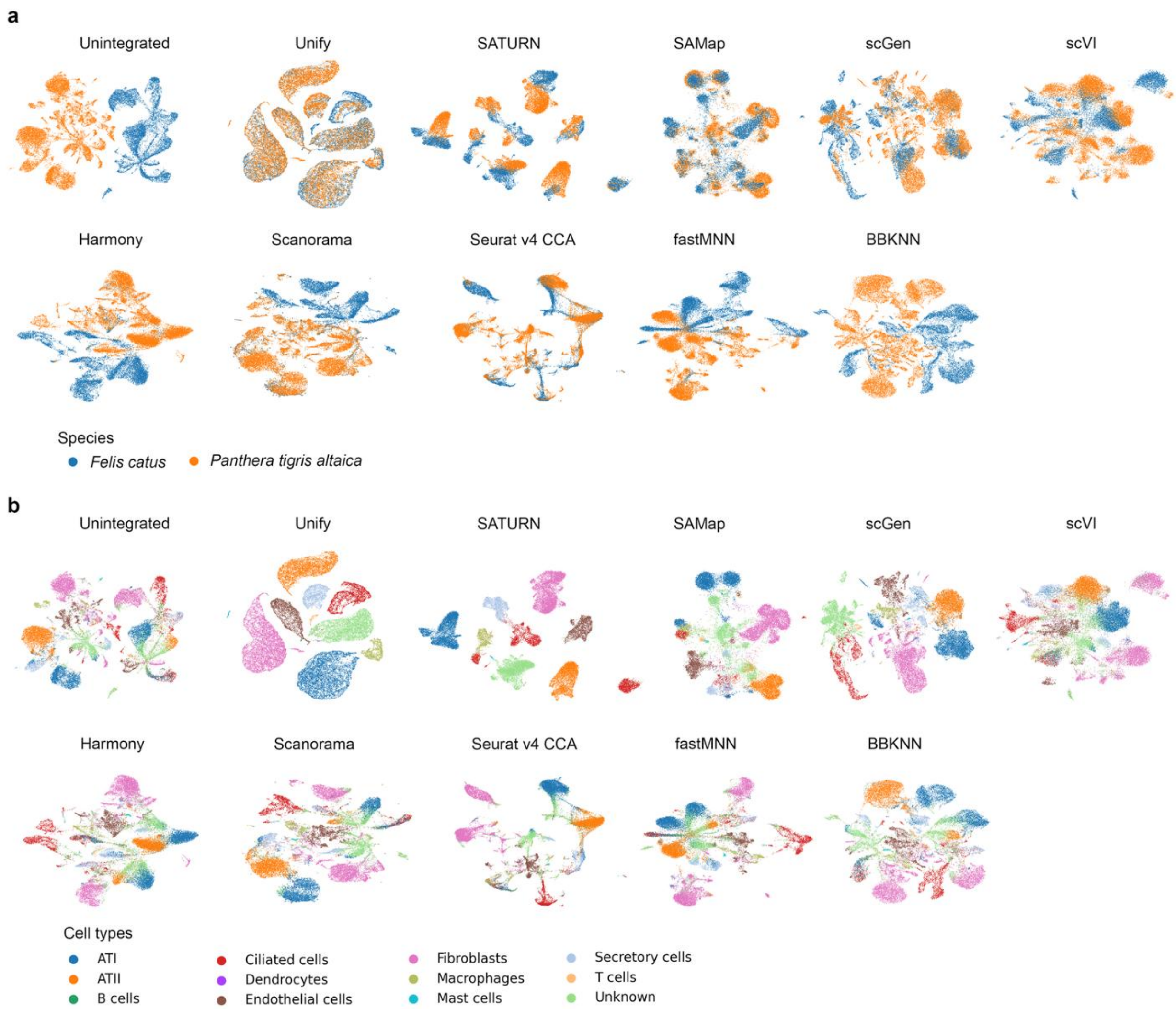

Supplementary Figure 1. UMAP layouts for the unintegrated and integrated *Felis catus* (cat) and *Panthera tigris altaica* (tiger) datasets (task1). (**a**) Plots are colored by species labels. (**b**) Plots are colored by cell type labels.

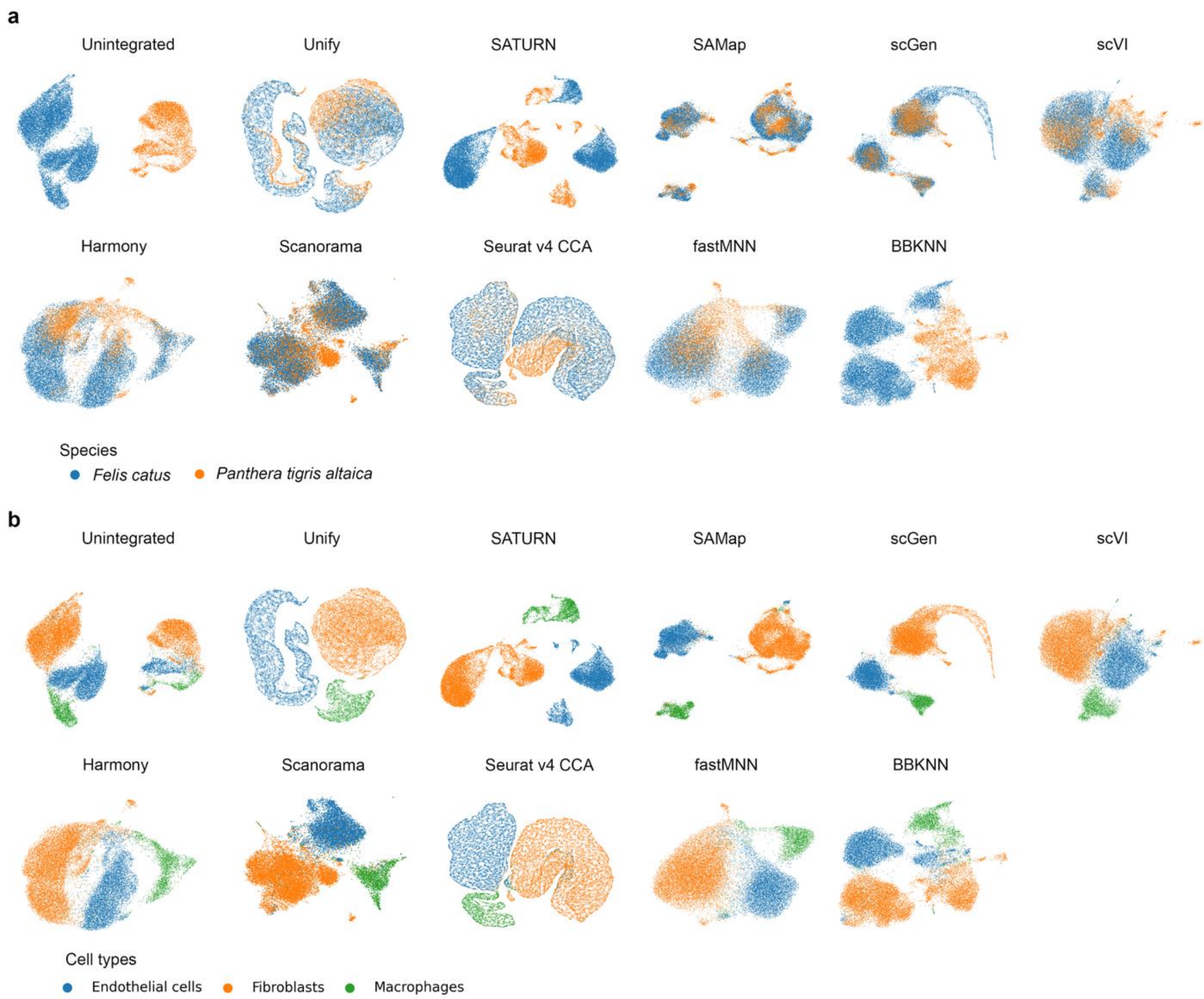

Supplementary Figure 2. UMAP layouts for the unintegrated and integrated *Felis catus* (cat) and *Panthera tigris altaica* (tiger) datasets (task2) from different tissues. (**a**) Plots are colored by species labels. (**b**) Plots are colored by cell type labels.

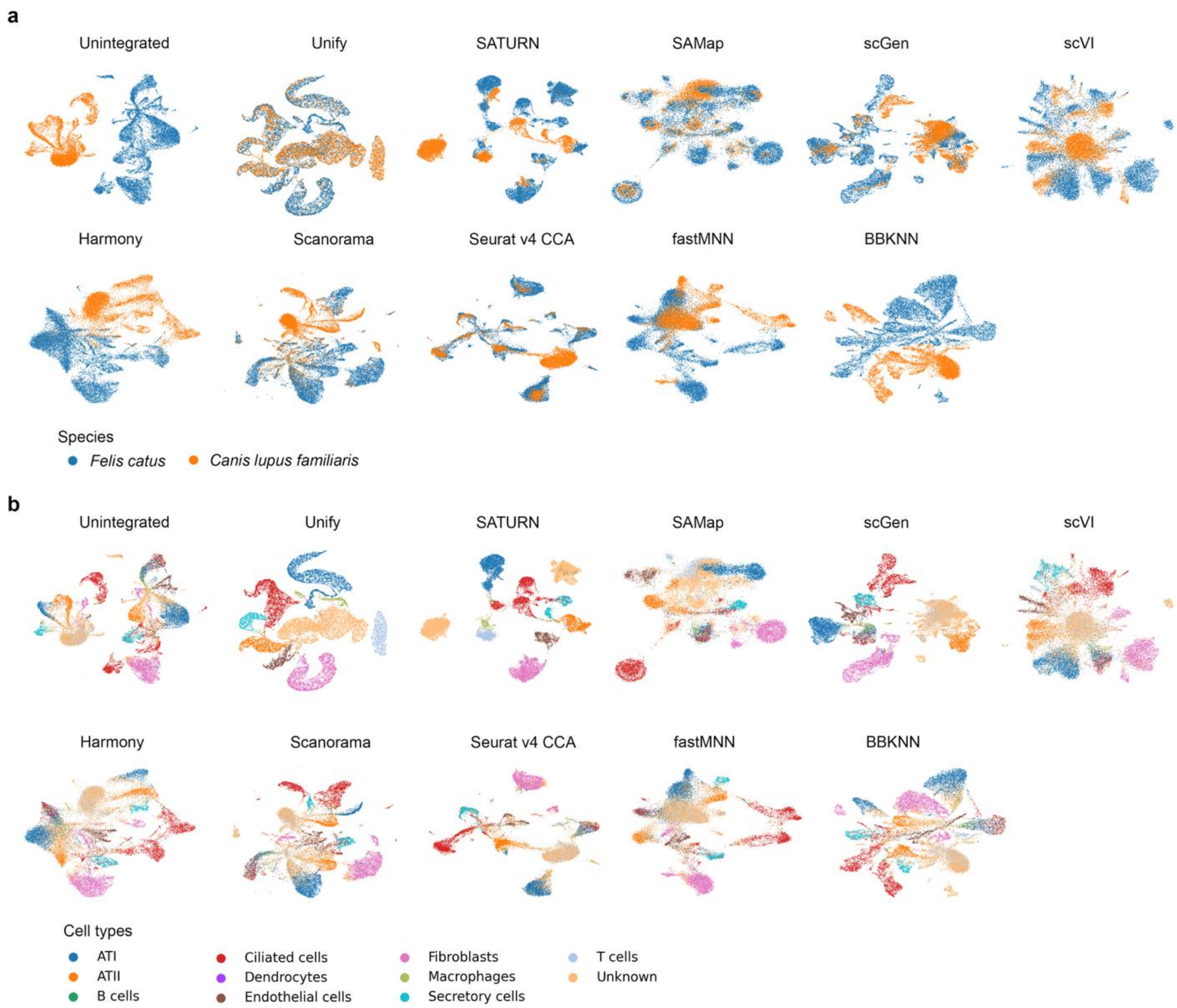

Supplementary Figure 3. UMAP layouts for the unintegrated and integrated *Felis catus* (cat) and *Canis lupus familiaris* (dog) datasets (task3). (**a**) Plots are colored by species labels. (**b**) Plots are colored by cell type labels.

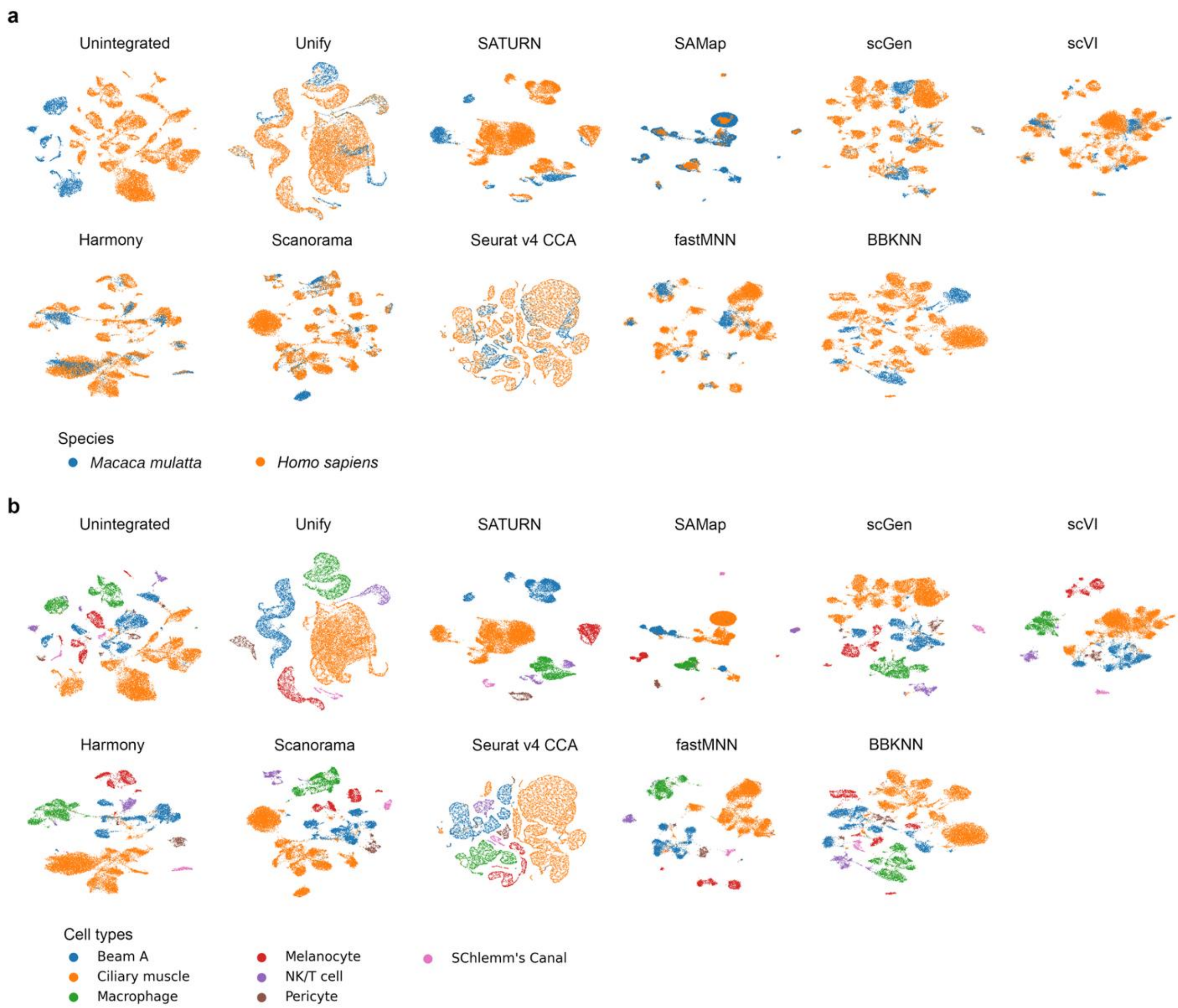

Supplementary Figure 4. UMAP layouts for the unintegrated and integrated *Homo sapiens* (human) and *Macaca mulatta* (monkey) datasets (task4). (**a**) Plots are colored by species labels. (**b**) Plots are colored by cell type labels.

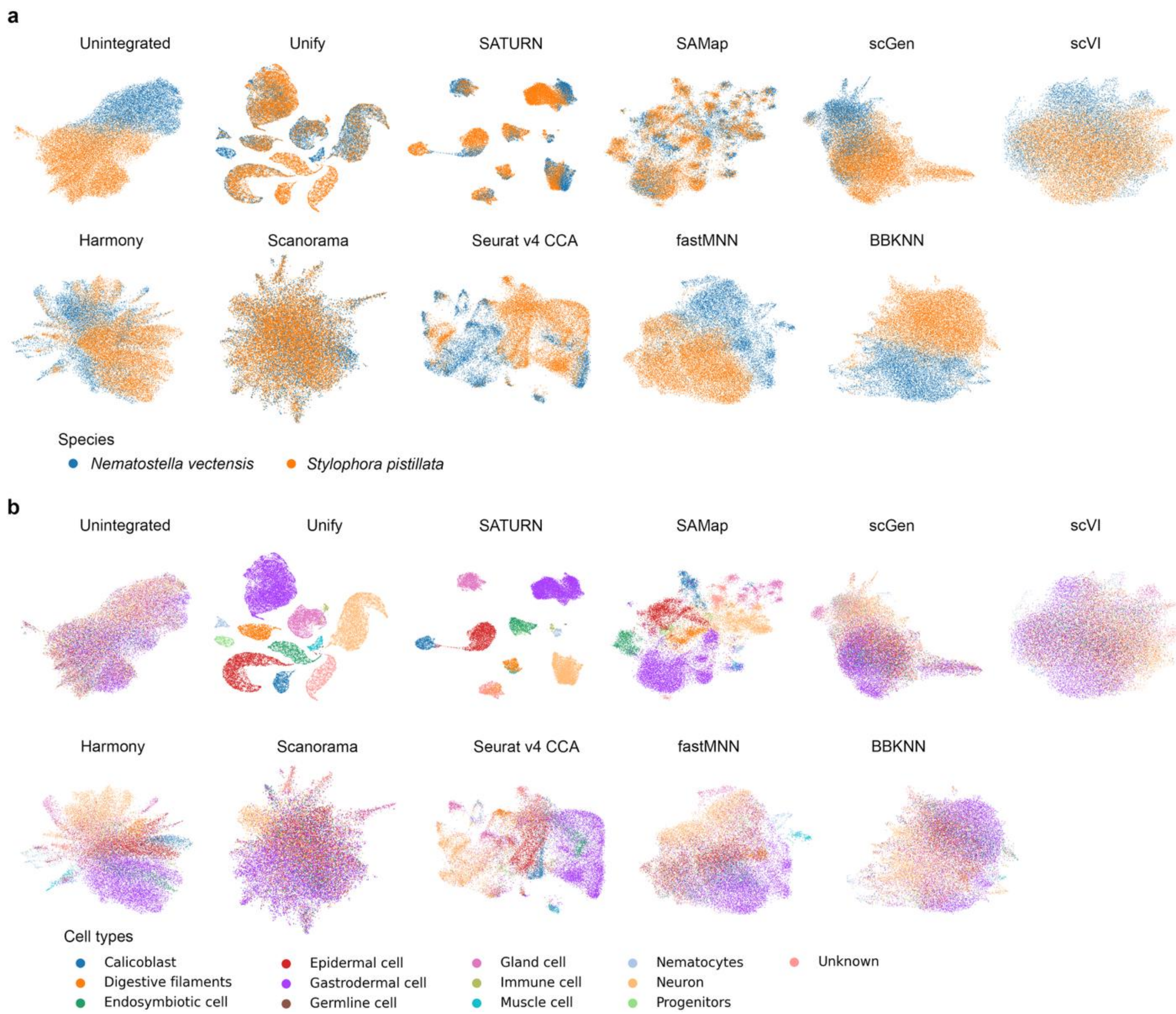

Supplementary Figure 5. UMAP layouts for the unintegrated and integrated *Nematostella vectensis* (sea anemone) and *Stylophora pistillata* (hard coral) datasets (task5). (**a**) Plots are colored by species labels. (**b**) Plots are colored by cell type labels.

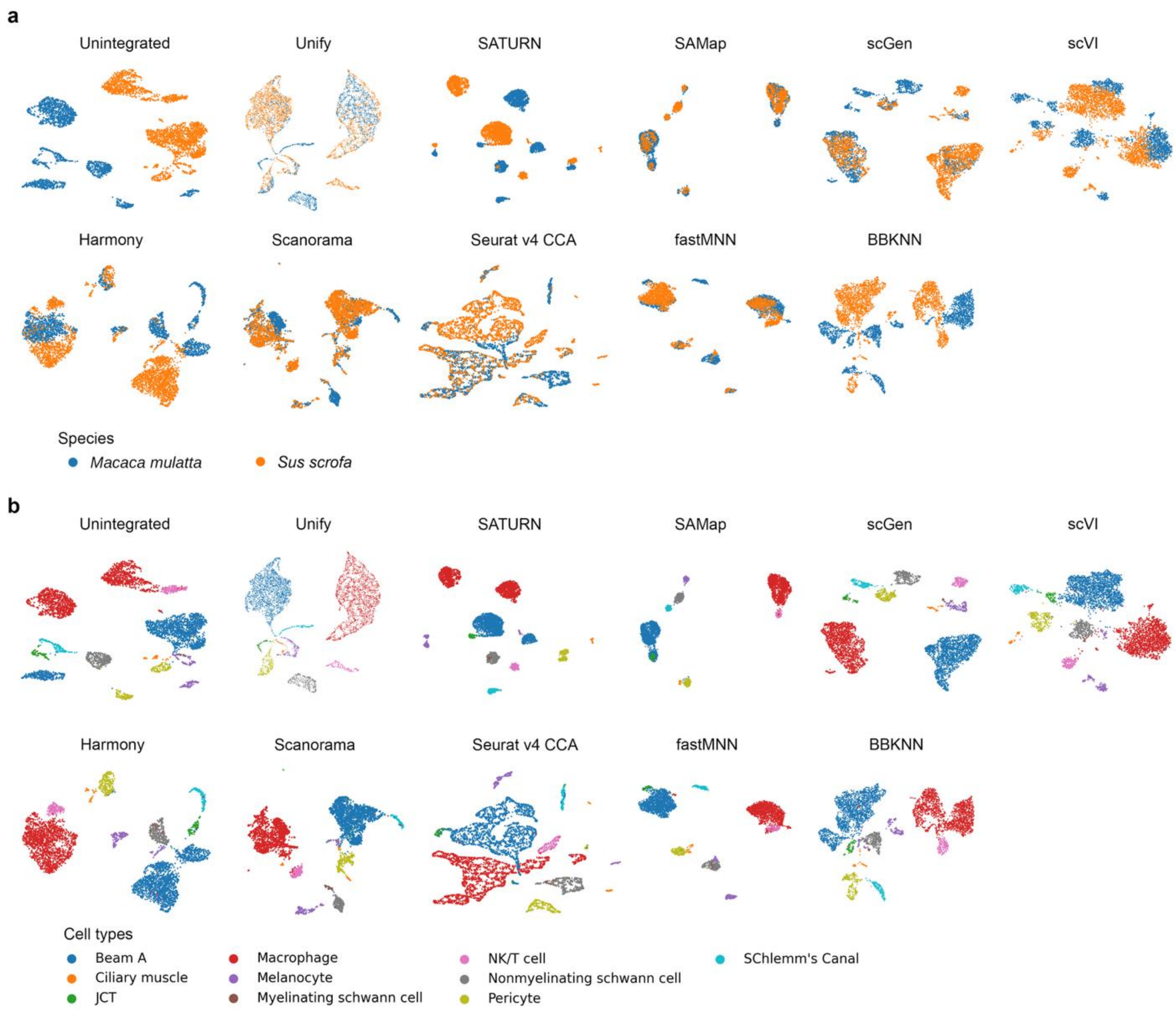

Supplementary Figure 6. UMAP layouts for the unintegrated and integrated *Macaca mulatta* (monkey) and *Sus scrofa* (pig) datasets (task6). (**a**) Plots are colored by species labels. (**b**) Plots are colored by cell type labels.

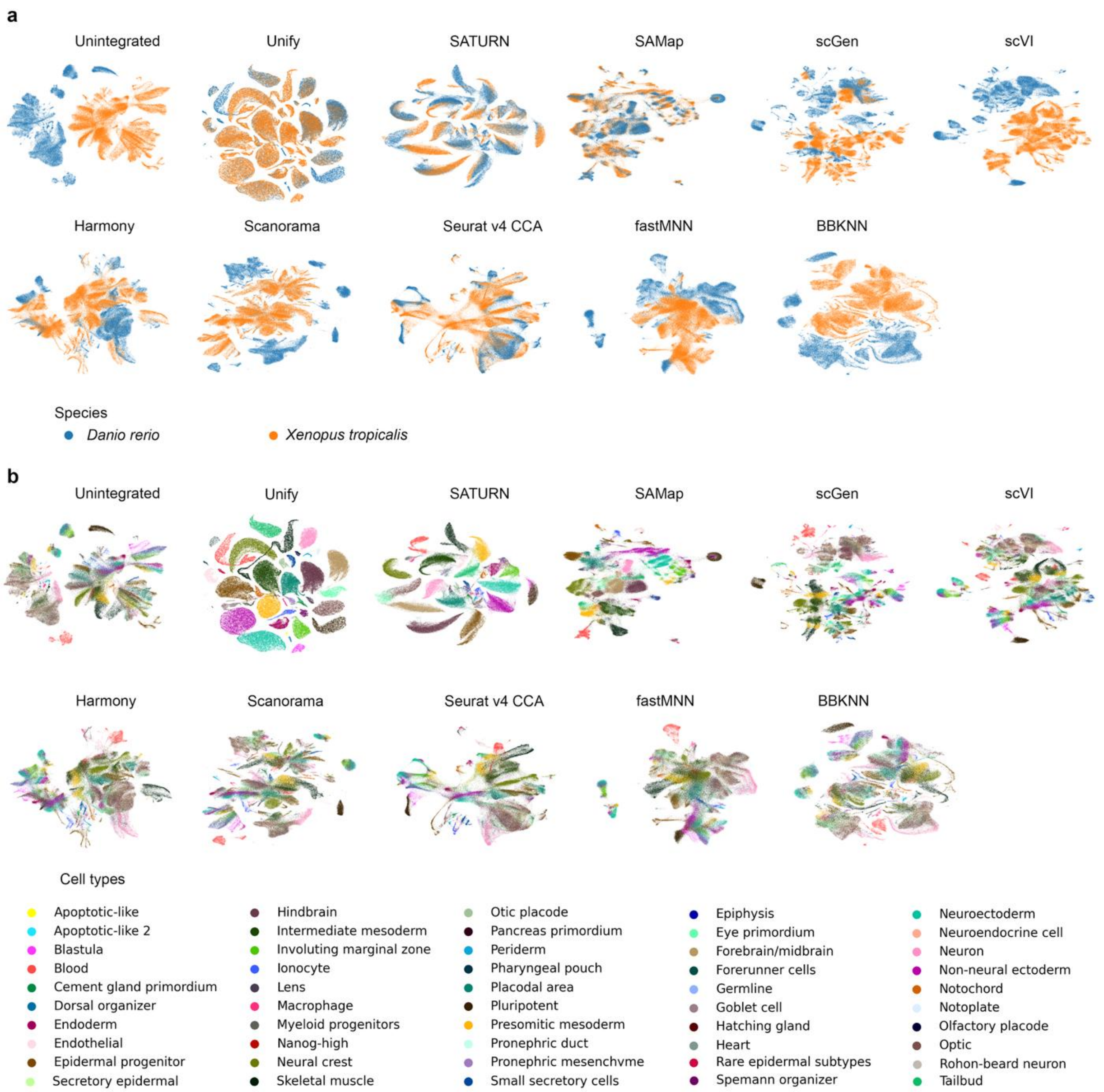

Supplementary Figure 7. UMAP layouts for the unintegrated and integrated *Danio rerio* (zebrafish) and *Xenopus tropicalis* (frog) datasets (task7). (**a**) Plots are colored by species labels. (**b**) Plots are colored by cell type labels.

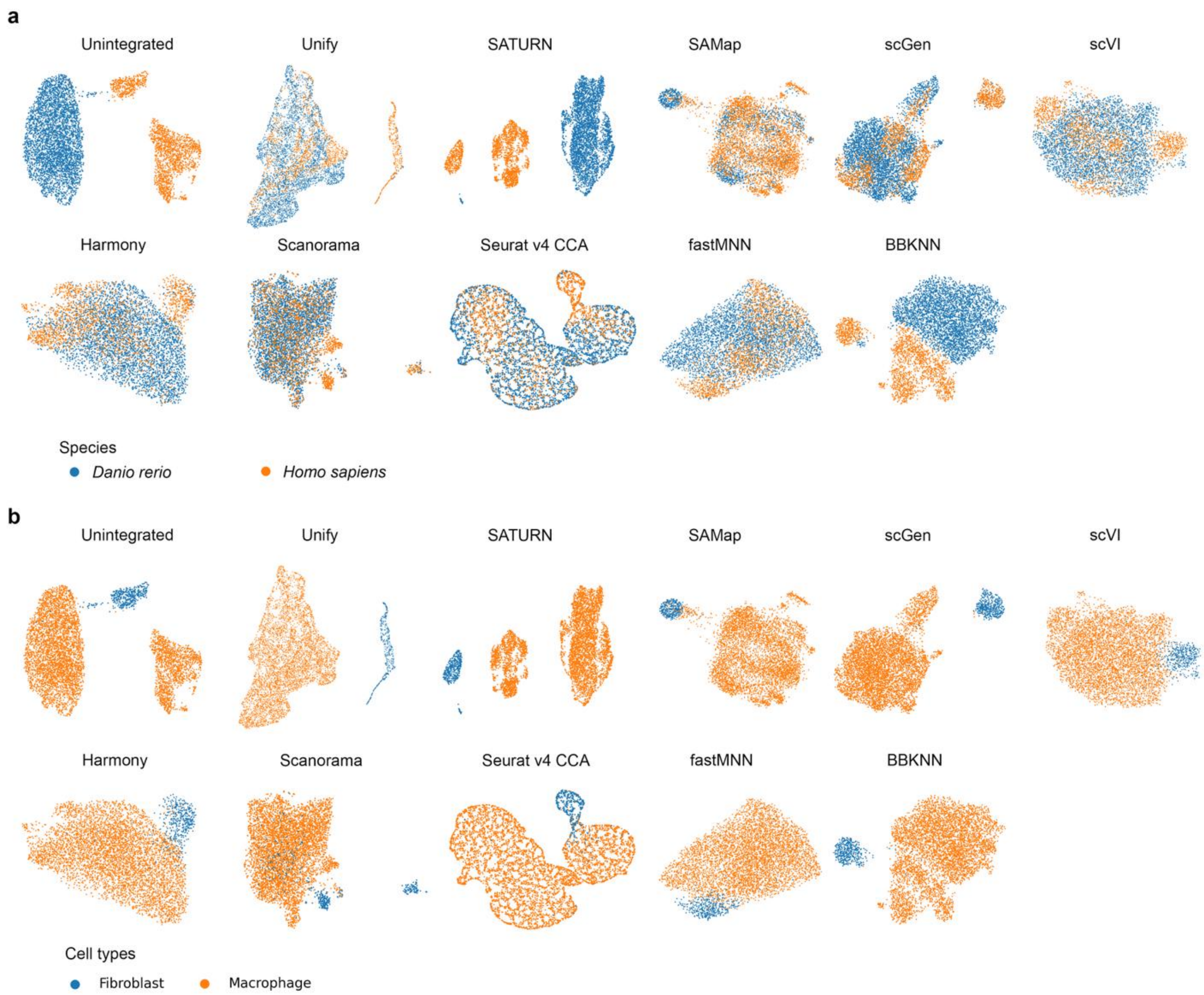

Supplementary Figure 8. UMAP layouts for the unintegrated and integrated *Danio rerio* (zebrafish) and *Homo sapiens* (human) datasets (task8). (**a**) Plots are colored by species labels. (**b**) Plots are colored by cell type labels.

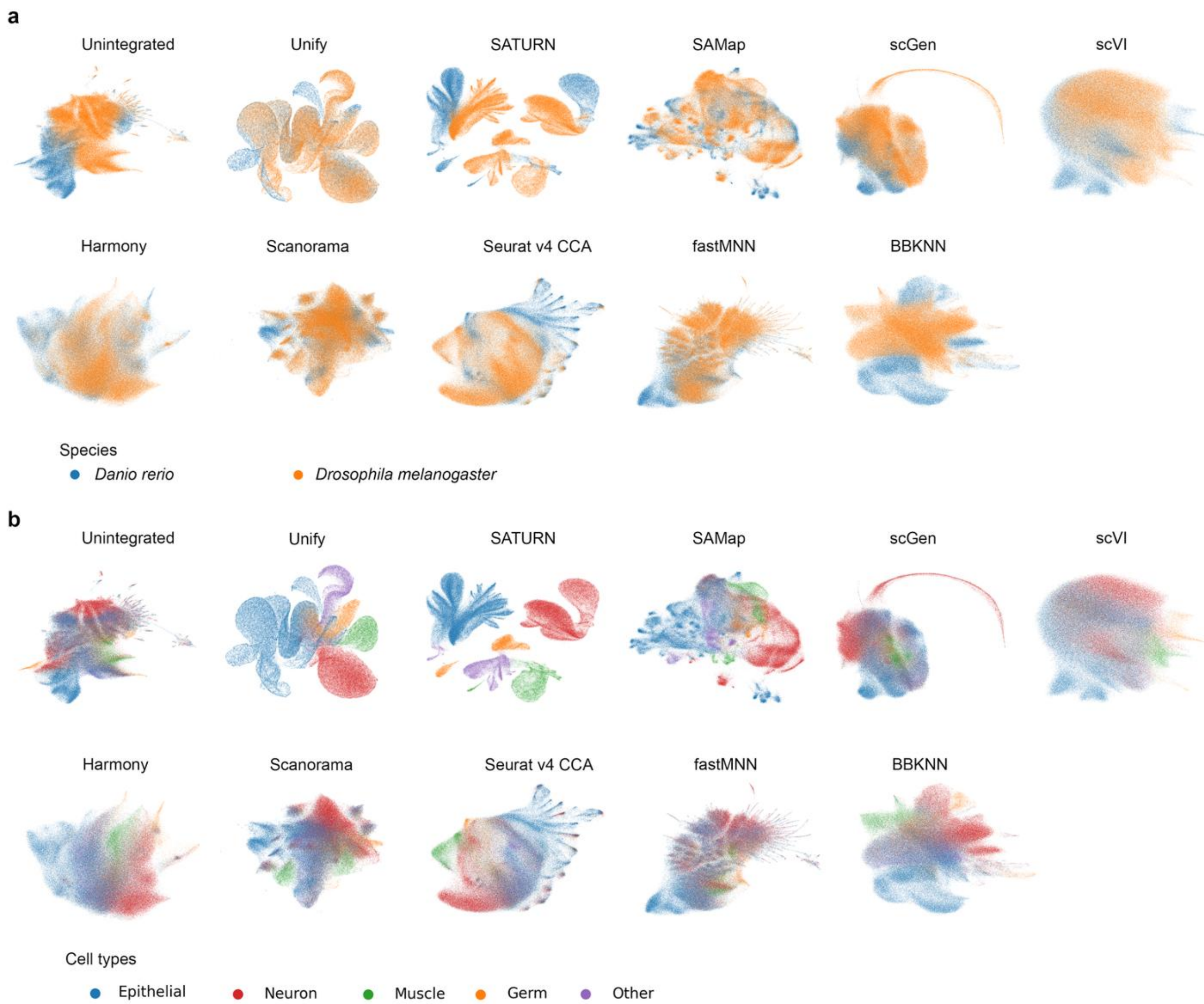

Supplementary Figure 9. UMAP layouts for the unintegrated and integrated *Danio rerio* (zebrafish) and *Drosophila melanogaster* (fly) datasets (task9). (**a**) Plots are colored by species labels. (**b**) Plots are colored by cell type labels.

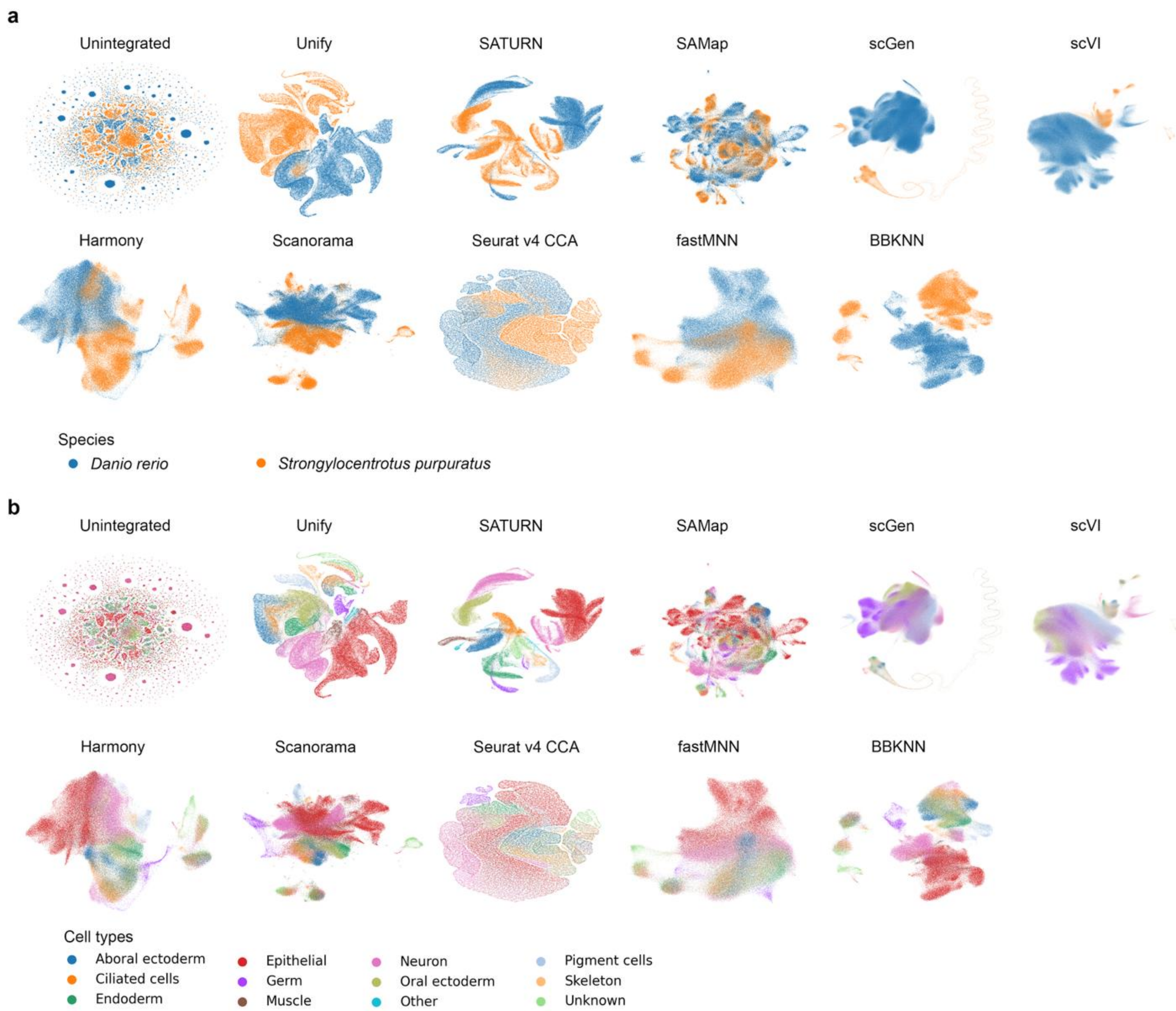

Supplementary Figure 10. UMAP layouts for the unintegrated and integrated *Strongylocentrotus purpuratus* (Sea urchin) and Danio rerio (Zebrafish) datasets (task10). (a) Plots are colored by species labels. (**b**) Plots are colored by cell type labels.

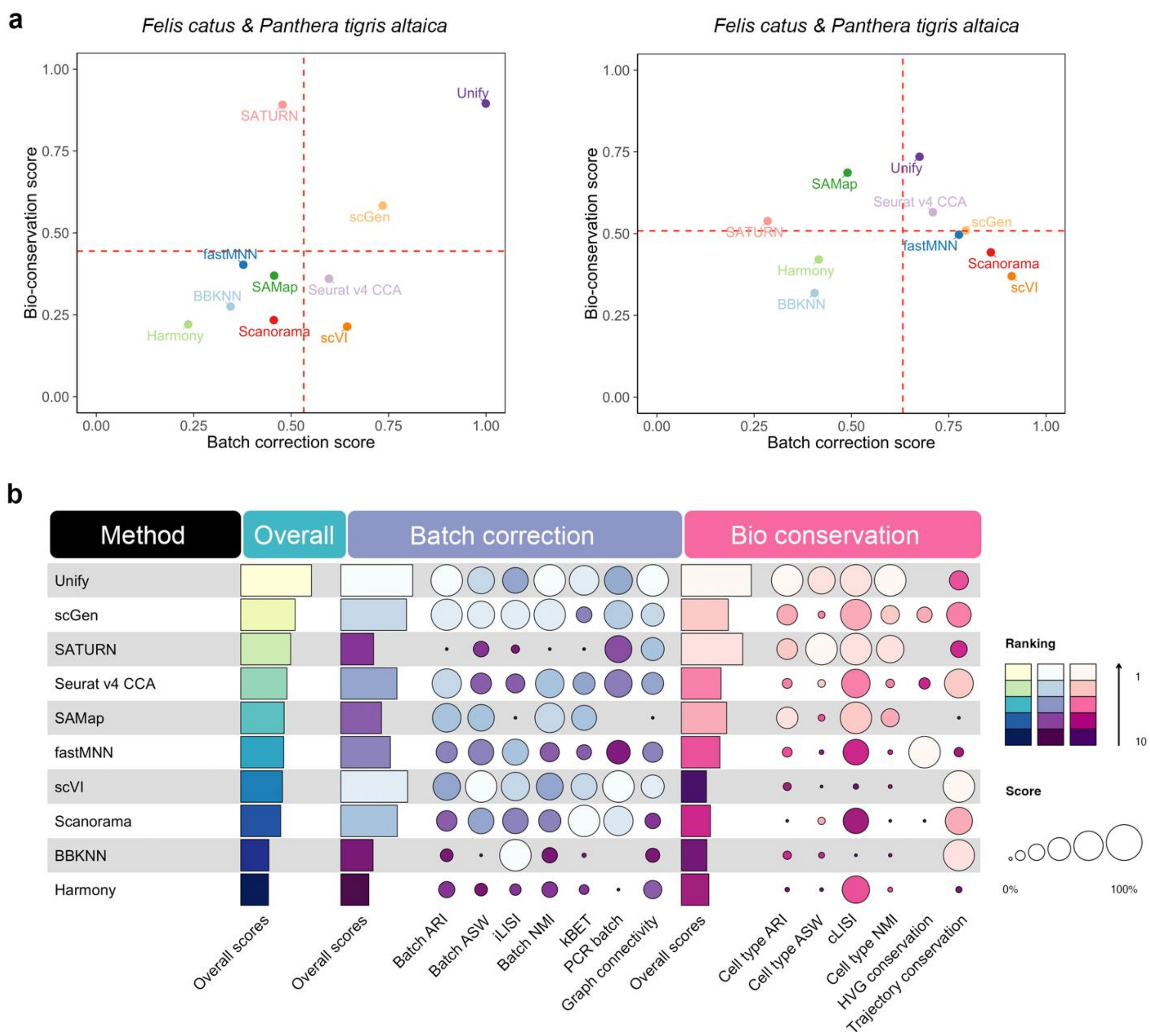

Supplementary Figure 11. Benchmark results for the cross-genus species integration tasks. (**a**) Scatter plot of the overall batch correction scores against overall bio-conservation scores for ten methods in all the cross-genus species integration tasks. The vertical dashed line represents the average batch correction score for all methods. The horizontal dashed line represents the average bio-conservation score for all methods. (**b**) Overview of the ten ranked methods for cross-genus species integration. Methods are ordered by their mean overall score across all cross-genus integration tasks. Each displayed value represents the average score across tasks. Source data are provided as a Source Data file.

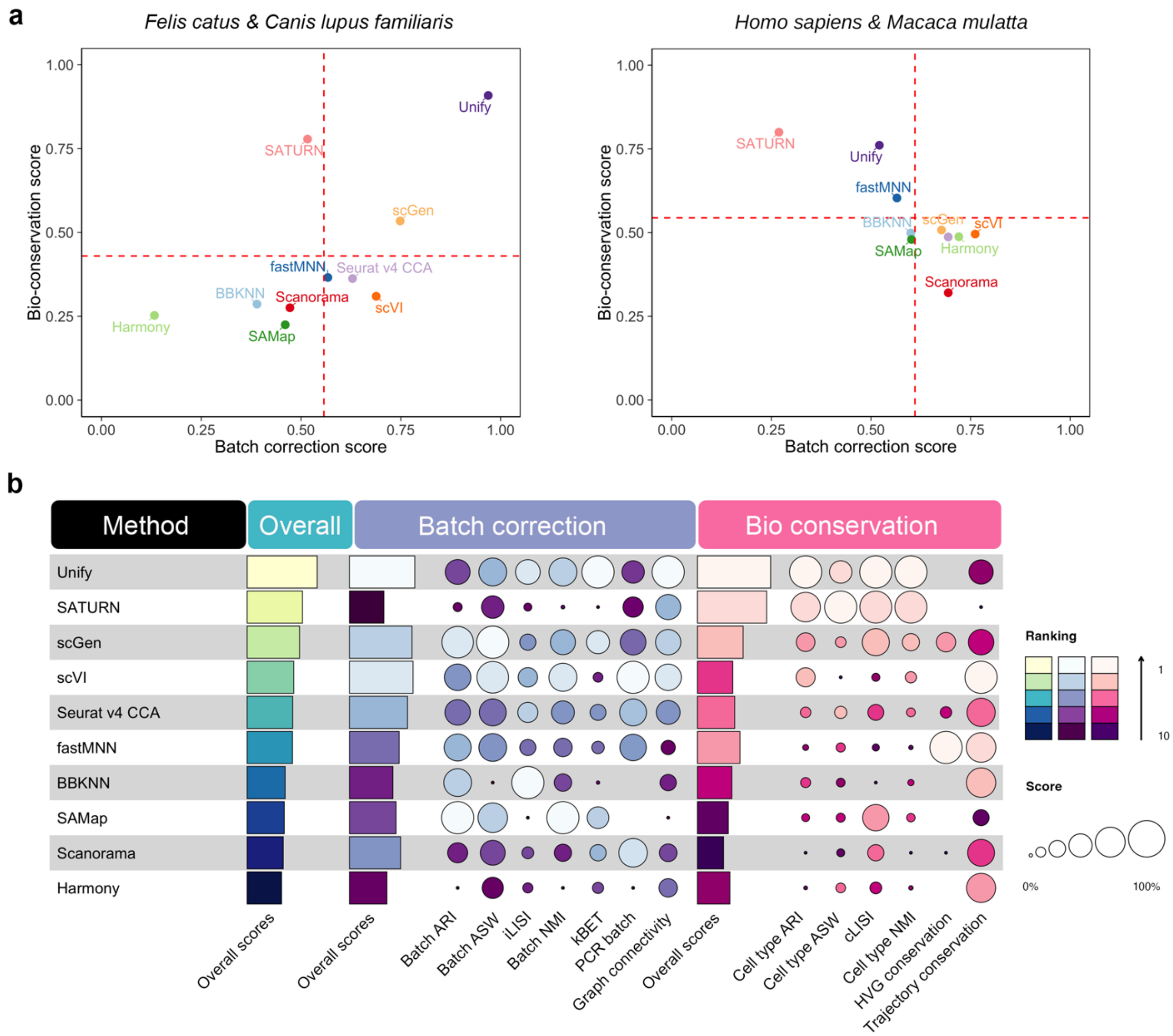

Supplementary Figure 12. Benchmark results for the cross-family species integration tasks. (**a**) Scatter plot of the overall batch correction scores against overall bio-conservation scores for ten methods in all the cross-family species integration tasks. The vertical dashed line represents the average batch correction score for all methods. The horizontal dashed line represents the average bio-conservation score for all methods. (**b**) Overview of the ten ranked methods for cross-family species integration. Methods are ordered by their mean overall score across all cross-family integration tasks. Each displayed value represents the average score across tasks. Source data are provided as a Source Data file.

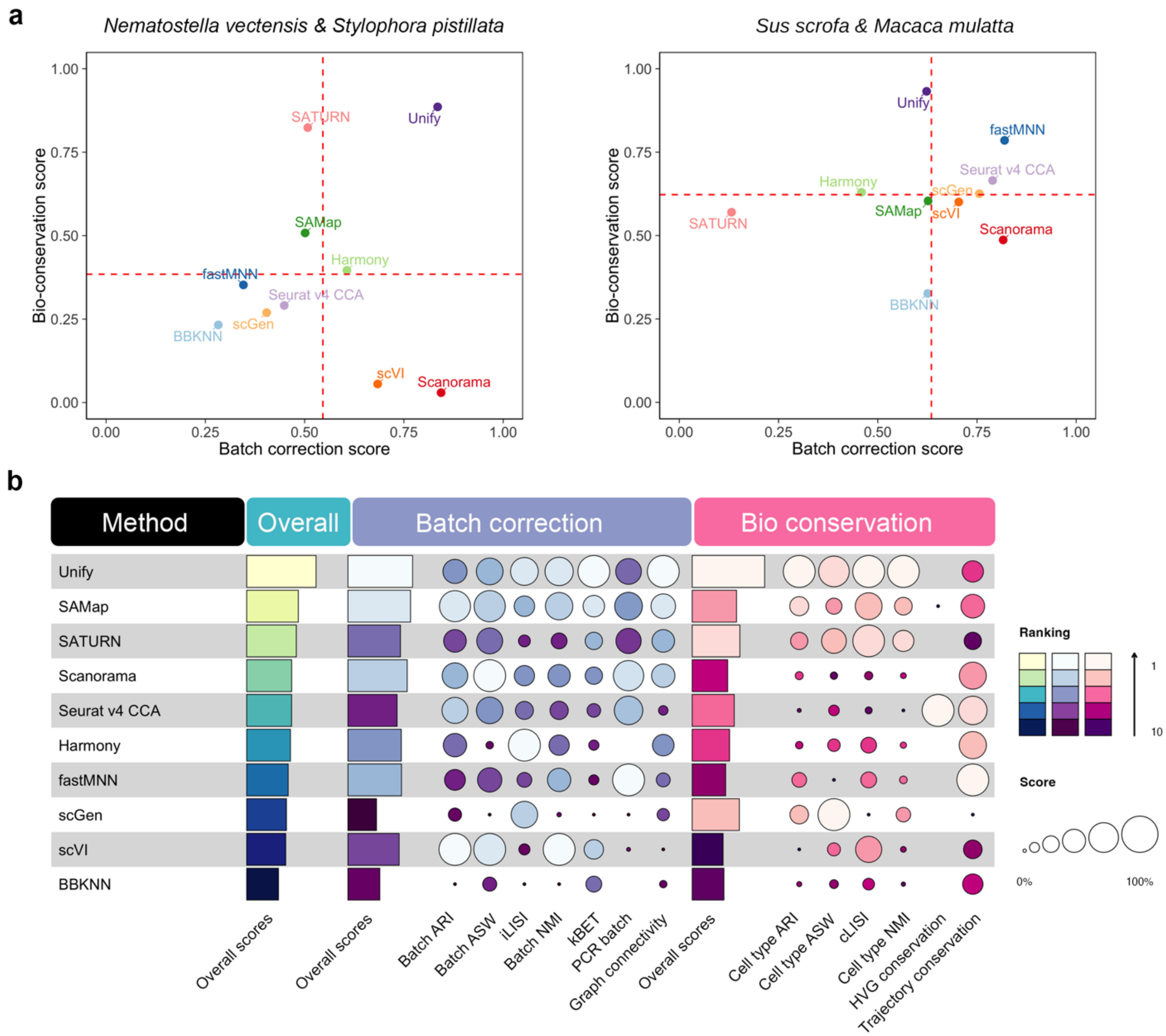

Supplementary Figure 13. Benchmark results for the cross-order species integration tasks. (**a**) Scatter plot of the overall batch correction scores against overall bio-conservation scores for ten methods in all the cross-order species integration tasks. The vertical dashed line represents the average batch correction score for all methods. The horizontal dashed line represents the average bio-conservation score for all methods. (**b**) Overview of the ten ranked methods for cross-order species integration. Methods are ordered by their mean overall score across all cross-order integration tasks. Each displayed value represents the average score across tasks. Source data are provided as a Source Data file.

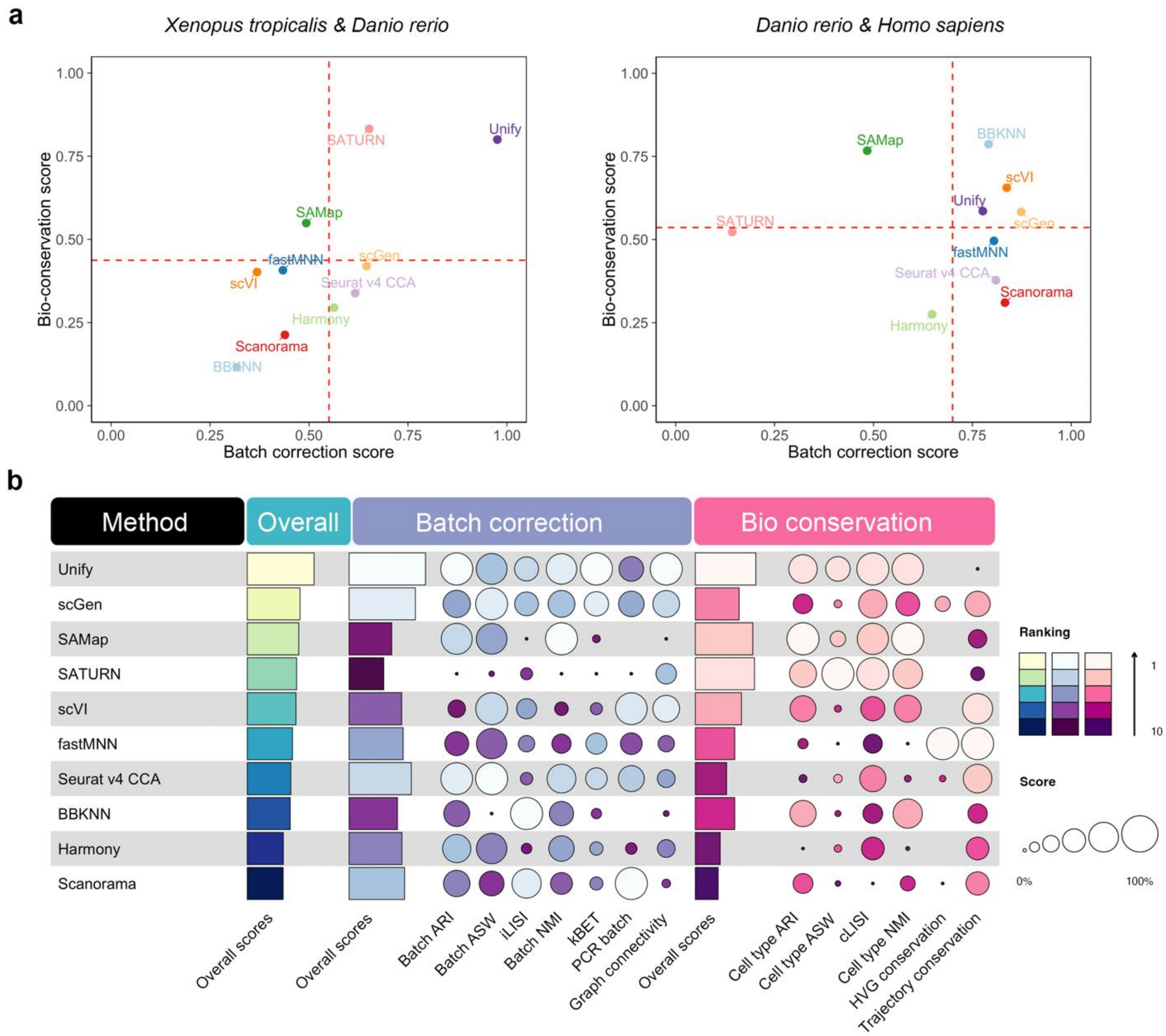

Supplementary Figure 14. Benchmark results for the cross-class species integration tasks. (**a**) Scatter plot of the overall batch correction scores against overall bio-conservation scores for ten methods in all the cross-class species integration tasks. The vertical dashed line represents the average batch correction score for all methods. The horizontal dashed line represents the average bio-conservation score for all methods. (**b**) Overview of the ten ranked methods for cross-class species integration. Methods are ordered by their mean overall score across all cross-class integration tasks. Each displayed value represents the average score across tasks. Source data are provided as a Source Data file.

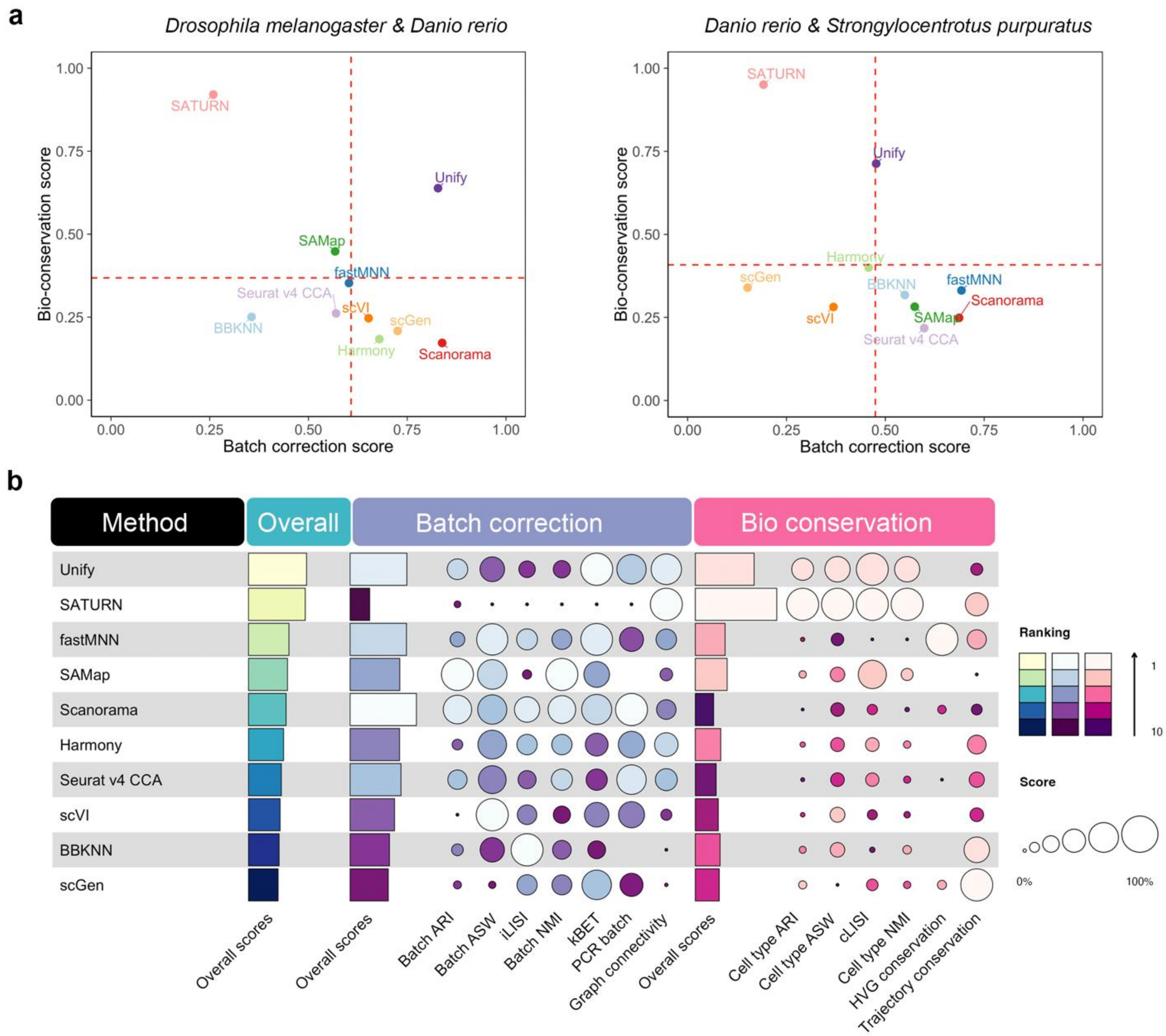

Supplementary Figure 15. Benchmark results for the cross-phylum species integration tasks. (**a**) Scatter plot of the overall batch correction scores against overall bio-conservation scores for ten methods in all the cross-phylum species integration tasks. The vertical dashed line represents the average batch correction score for all methods. The horizontal dashed line represents the average bio-conservation score for all methods. (**b**) Overview of the ten ranked methods for cross-phylum species integration. Methods are ordered by their mean overall score across all cross-phylum integration tasks. Each displayed value represents the average score across tasks. Source data are provided as a Source Data file.

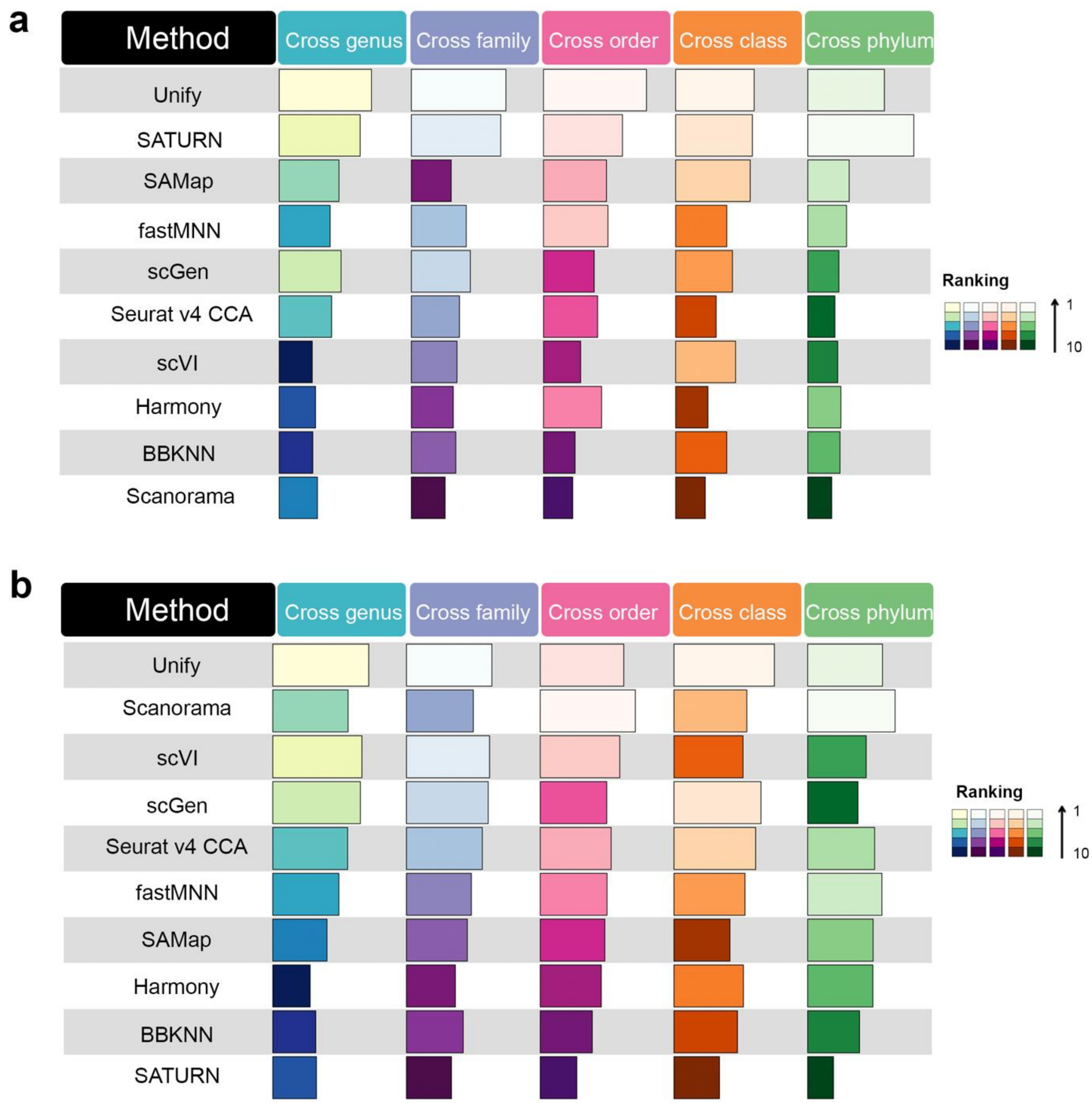

Supplementary Figure 16. Overall performance of all benchmarked methods across cross-species integration tasks stratified by phylogenetic distance between the species. (**a**) Bio-conservation scores. (**b**) Batch-correction scores. Source data are provided as a Source Data file.

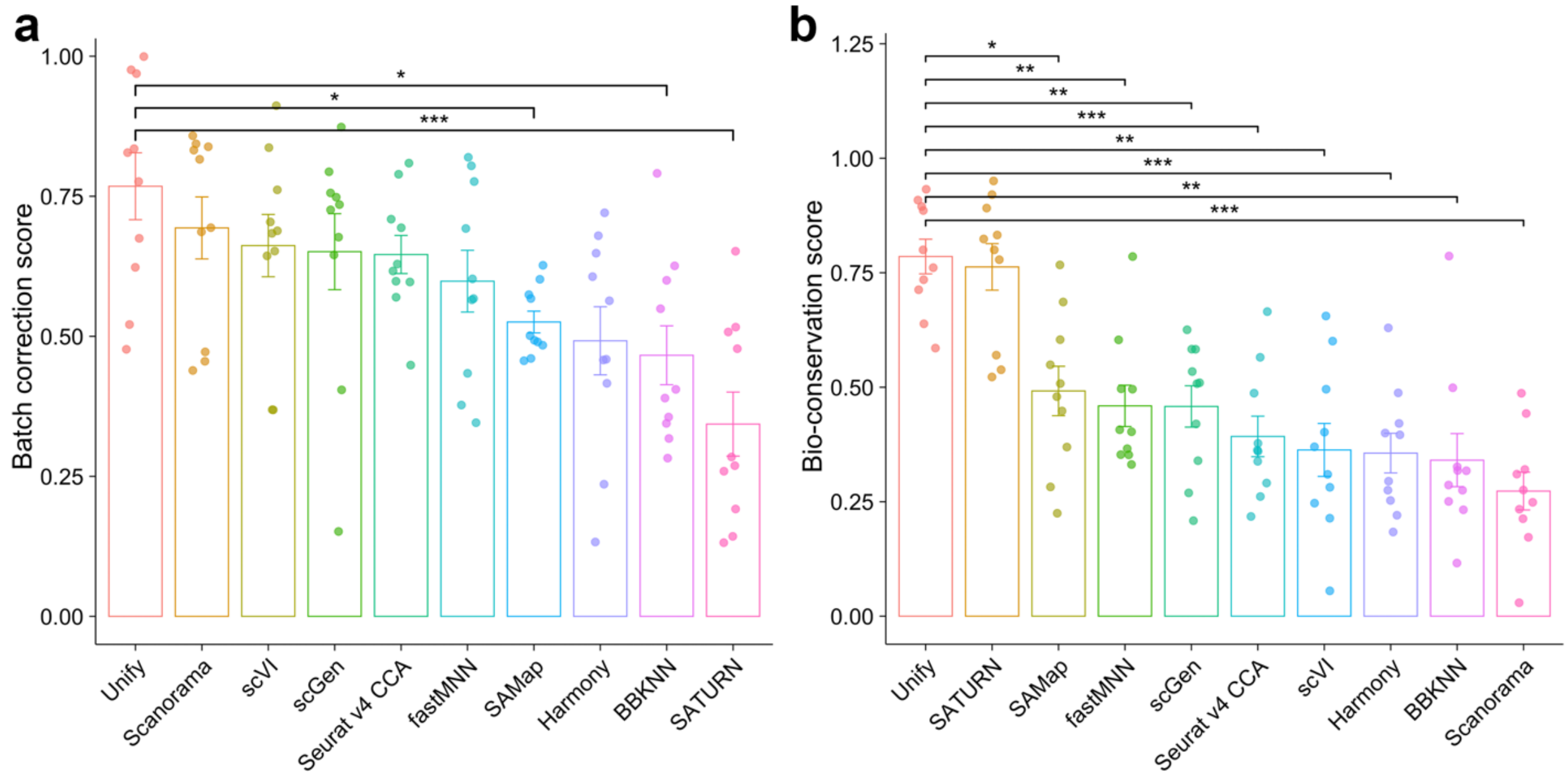

Supplementary Figure 17. Average performance across cross-species integration tasks (n=10 tasks). (**a**) Bar plot showing the average batch-correction scores of the ten methods across all tasks. (**b**) Bar plot showing the average bio-conservation scores across all tasks. Bars represent average values across tasks, and error bars indicate the standard error (s.e.) of the mean. Individual points represent the score obtained from each integration task. Statistical significance between methods was assessed using paired two-sided *t*-tests across tasks, and Benjamini-Hochberg-adjusted *P* values are indicated (*** adjusted $P<0.001$, ** adjusted $P < 0.01$, * adjusted $P<0.05$). Source data are provided as a Source Data file.

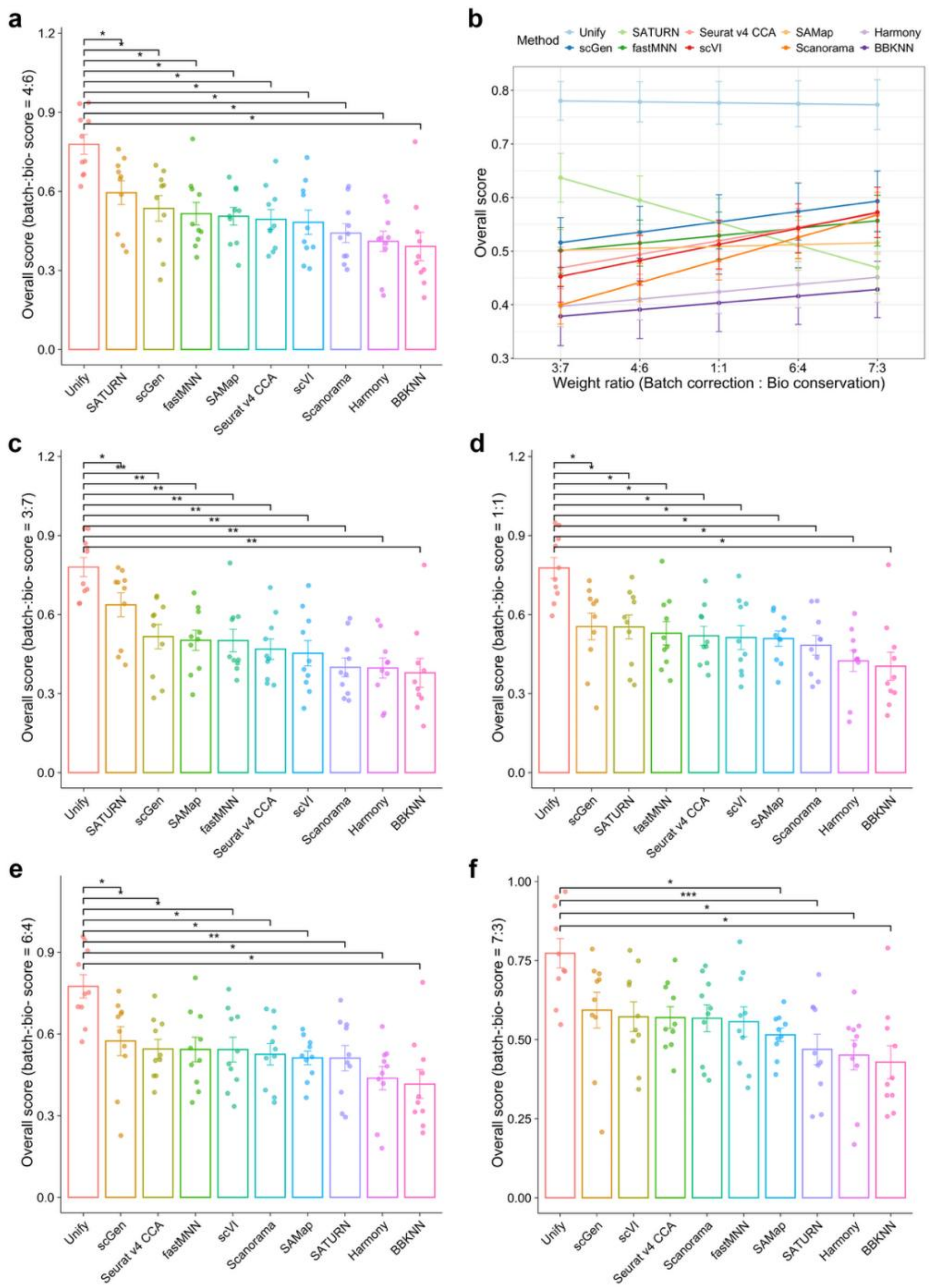

Supplementary Figure 18. Sensitivity of average overall performance to different weightings of batch-correction and bio-conservation scores across cross-species integration tasks (n=10 tasks). (**a**) Bar plot showing the average overall scores ± standard error (s.e.) of the ten methods using the 4:6 weighting between batch correction and bio-conservation adopted in this study. (**b**) Line plot showing the average overall score ± s.e. for each method across different weighting schemes. (**c-f**) Bar plots showing the mean overall scores across all tasks using batch-correction:bio-conservation weightings of 3:7 (**c**), 1:1 (**d**), 6:4 (**e**), and 7:3 (**f**). Individual points represent the score obtained from each integration task. Statistical significance between methods was assessed using paired two-sided *t*-tests across tasks, and Benjamini-Hochberg-adjusted *P* values are indicated (*** adjusted $P<0.001$, ** adjusted $P<0.01$, * adjusted $P<0.05$). Source data are provided as a Source Data file.

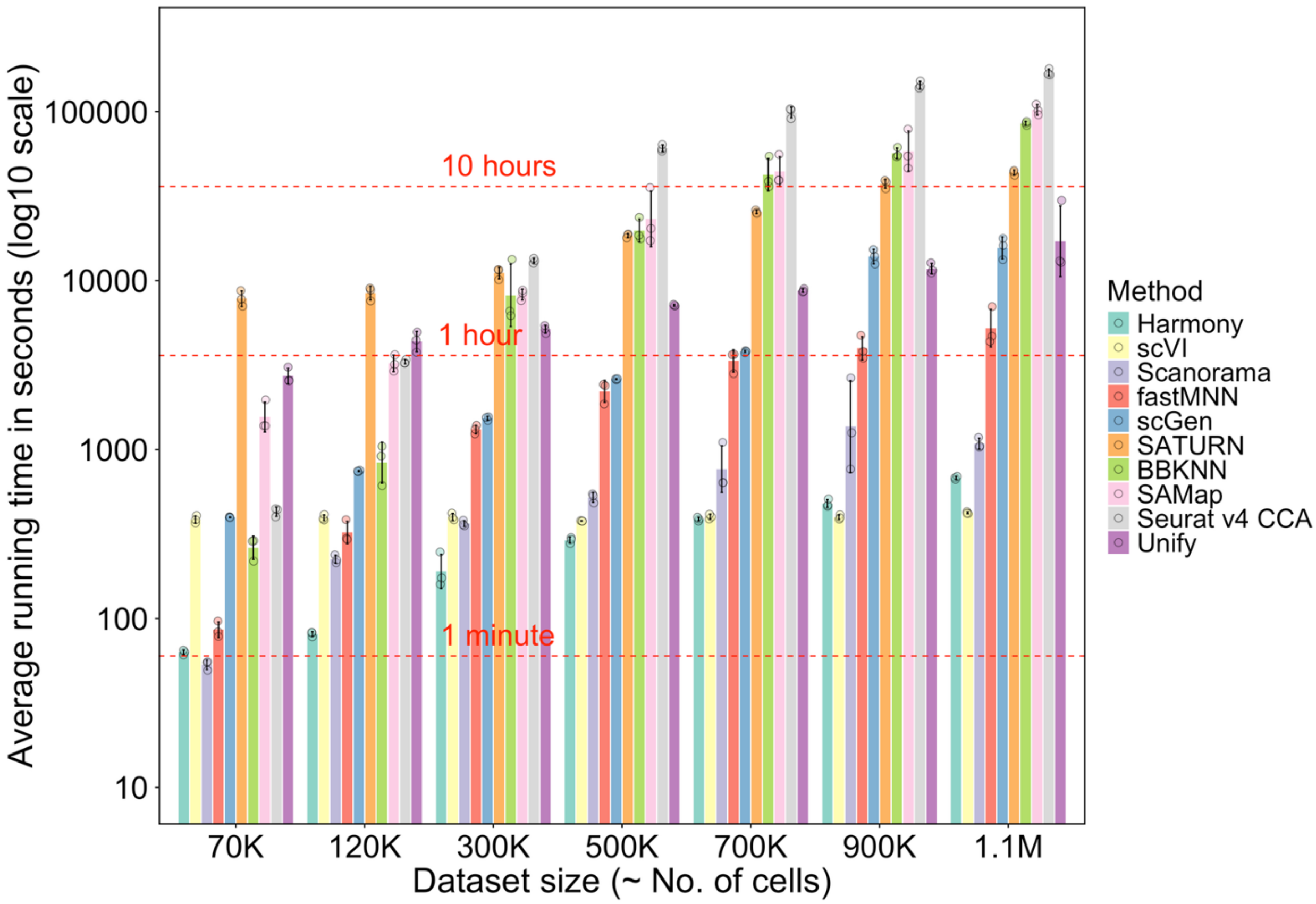

Supplementary Figure 19. Average running time of benchmarked methods across integration tasks with different dataset sizes. Bar plot showing the average running time of the ten benchmarked methods across datasets of increasing size, ranging from approximately 70K to 1.1M cells. Running time was measured for three replicate runs per method and dataset, and values were log10-transformed before plotting. Bars represent the mean running time across replicates, error bars indicate the standard deviation, and individual points represent the running time of each replicate. Red dashed horizontal lines indicate reference running times of 1 minute, 1 hour, and 10 hours. Source data are provided as a Source Data file.

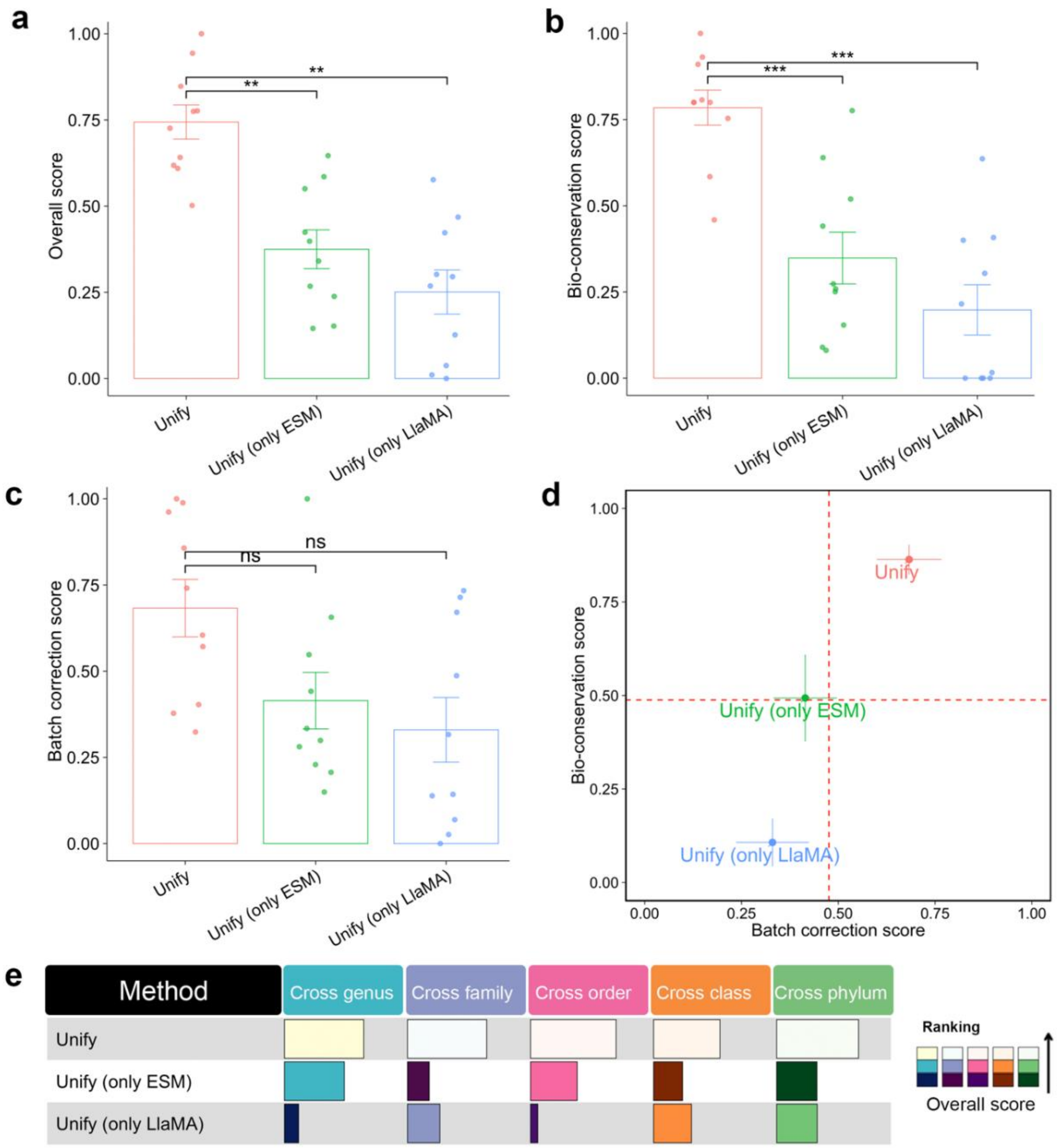

Supplementary Figure 20. Ablation study comparing the use of ESM embeddings alone, LLaMA embeddings alone, or both embeddings across cross-species integration tasks (n=10 tasks). Bar plots show the mean ± standard error for the three model variants in overall score (**a**), bio-conservation score (**b**), and batch-correction score (**c**). Individual points represent the score obtained from each integration task. (**d**) Scatter plot showing average overall batch-correction scores versus average overall bio-conservation scores with the standard error across all species integration tasks for the three model variants. The vertical dashed line indicates the mean batch-correction score across all methods, and the horizontal dashed line indicates the mean bio-conservation score across all methods. Statistical significance between methods was assessed using paired *t*-tests across tasks, and Benjamini-Hochberg-adjusted *P* values are indicated (***adjusted *P*<0.001, **adjusted *P*<0.01, *adjusted *P*<0.05, ns: no significant difference). (**e**) Overall performance of the model variants across cross-species integration tasks stratified by phylogenetic distance between the species. Source data are provided as a Source Data file.

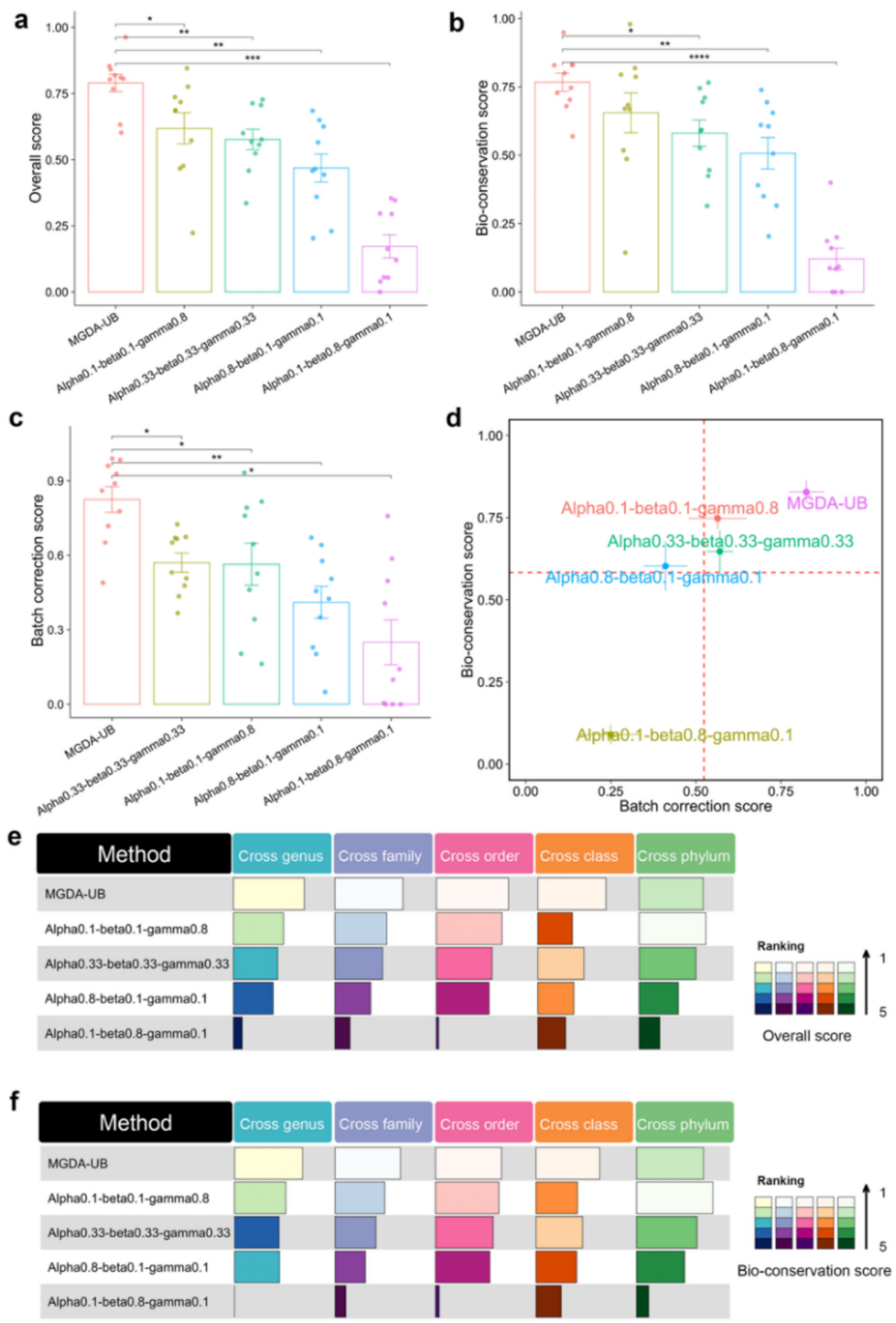

Supplementary Figure 21. Ablation study comparing MGDA-UB with fixed loss-weighting strategies across cross-species integration tasks (n=10 tasks). Bar plots show the mean ± standard error overall score (**a**), bio-conservation score (**b**), and batch-correction score (**c**) for Unify trained with MGDA-UB or with different fixed weight combinations of the reconstruction (alpha), species classifier (beta) and cell type classifier (gamma) losses. Individual point represents the score obtained from one integration task for the corresponding model variant. (**d**) Scatter plot of average overall batch-correction scores versus average overall bio-conservation scores with standard error for all weighting strategies across tasks. The vertical dashed line indicates the mean batch-correction score across all compared methods, and the horizontal dashed line indicates the mean bio-conservation score across all compared methods. Ranking summaries of the different weighting strategies across phylogenetic categories based on overall score (**e**) and bio-conservation score (**f**). Statistical significance between methods was assessed using paired two-sided *t*-tests across tasks, and Benjamini-Hochberg-adjusted *P* values are indicated (***adjusted *P*<0.001, **adjusted *P*<0.01, *adjusted *P*<0.05). Source data are provided as a Source Data file.

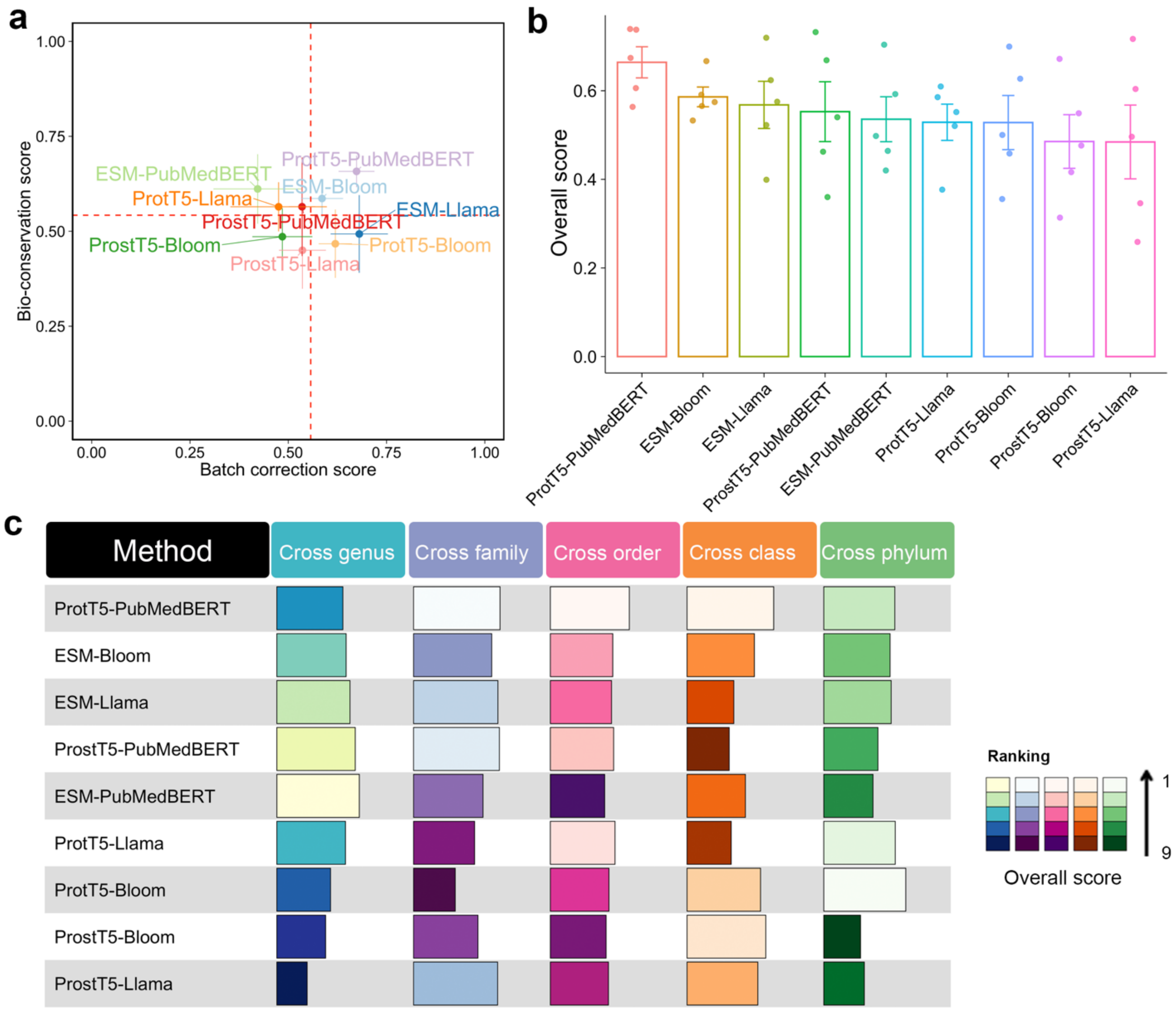

Supplementary Figure 22. Ablation study comparing different combinations of protein language models and large language models across cross-species integration tasks (n=5 tasks). (**a**) Scatter plot of average overall batch-correction scores versus average overall bio-conservation scores with standard error for Unify variants constructed with different protein language model and large language model combinations. The vertical dashed line indicates the mean batch-correction score across all compared variants, and the horizontal dashed line indicates the mean bio-conservation score across all compared variants. (**b**) Bar plot showing the mean overall scores ± standard error across all tasks for each embedding combination. Individual point represents the score obtained from one integration task for the corresponding model variant. (**c**) Ranking summary of the different embedding combinations across phylogenetic categories based on overall score. Source data are provided as a Source Data file.

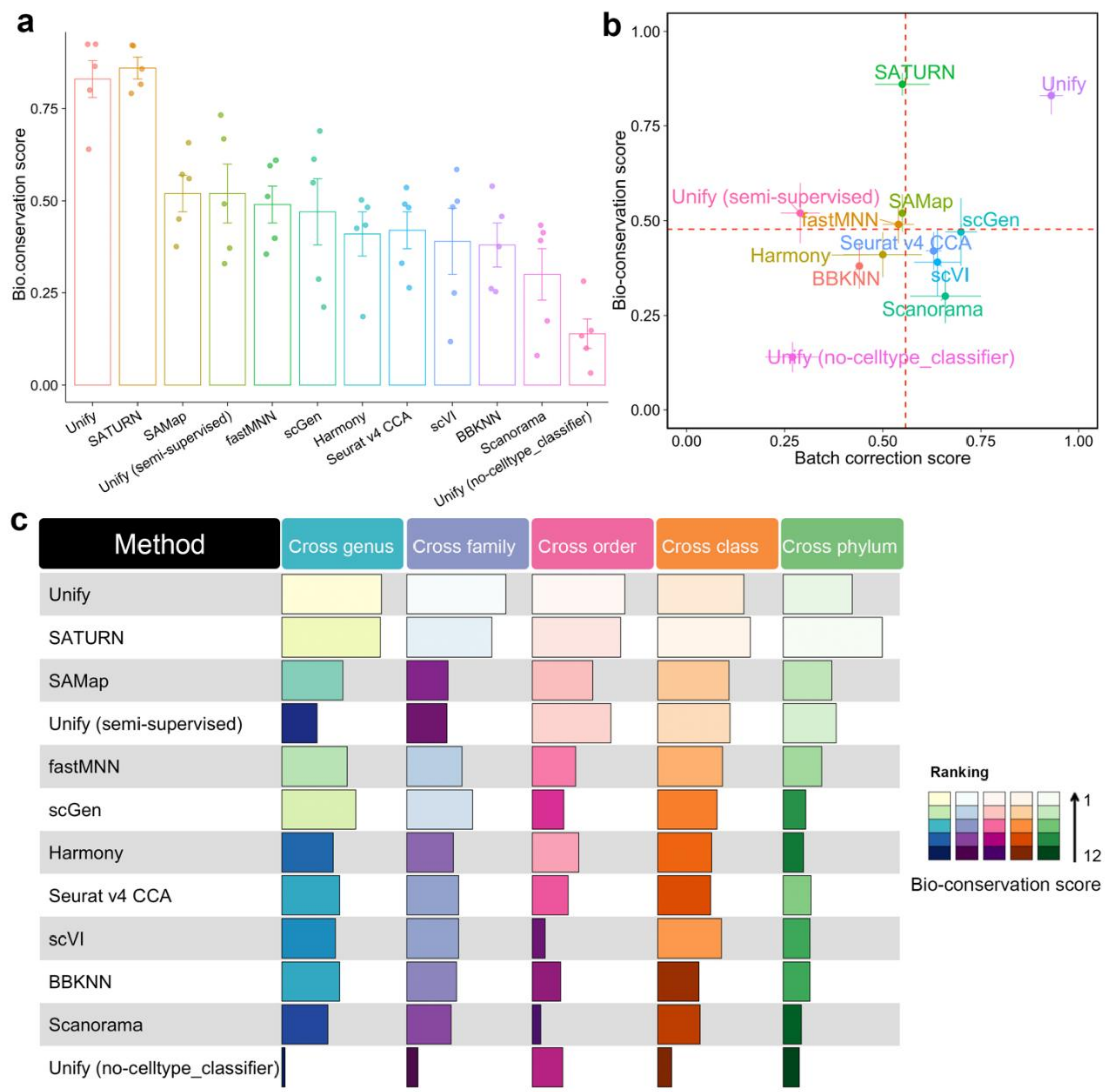

Supplementary Figure 23. Ablation study evaluating the influence of partial annotation supervision and the cell type classifier on bio-conservation performance across cross-species integration tasks (n=5 tasks). (**a**) Bar plot showing the mean bio-conservation scores ± standard error across all tasks for Unify, baseline methods, and two Unify ablation variants: Unify (semi-supervised), where one species is trained with cell type annotations while the other uses unsupervised cluster IDs, and Unify (no-celltype_classifier), where the cell type classifier is removed. Individual point represents the score obtained from one integration task for the corresponding model variant. (**b**) Scatter plot of average overall batch-correction scores versus average overall bio-conservation scores with standard error for all compared methods and ablation variants. The vertical dashed line indicates the mean batch-correction score across all compared methods, and the horizontal dashed line indicates the mean bio-conservation score across all compared methods. (**c**) Ranking summary of all compared methods and ablation variants across phylogenetic categories based on bio-conservation score. Source data are provided as a Source Data file.

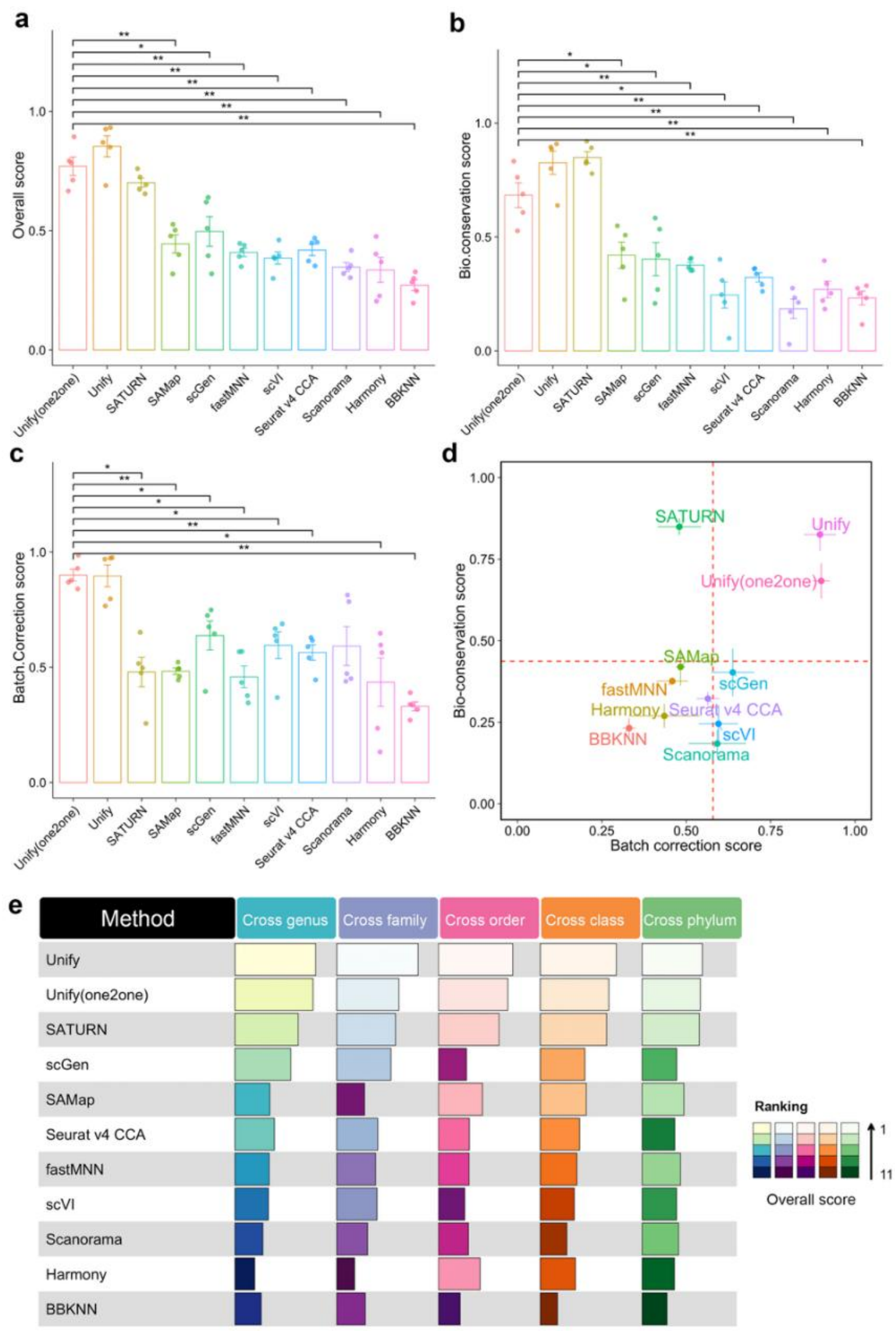

Supplementary Figure 24. Ablation study evaluating the use of one-to-one orthologs as input across cross-species integration tasks (n=5 tasks). Bar plots showing the mean ± standard error overall score (**a**), bio-conservation score (**b**) and batch-correction score (**c**) for Unify (one2one), the full Unify model, and the baseline methods across all tasks. Unify (one2one) denotes the variant in which the model is restricted to one-to-one orthologous genes as input. Individual point represents the score obtained from one integration task for the corresponding model variant. (**d**) Scatter plot of average overall batch-correction scores versus average overall bio-conservation scores with standard error for all compared methods and the Unify (one2one) variant. The vertical dashed line indicates the mean batch-correction score across all compared methods, and the horizontal dashed line indicates the mean bio-conservation score across all compared methods. (**e**) Ranking summary of all compared methods across phylogenetic categories based on overall score. Statistical significance between methods was assessed using paired two-sided *t*-tests across tasks, and Benjamini-Hochberg-adjusted *P* values are indicated (***adjusted *P*<0.001, **adjusted *P*<0.01, *adjusted *P*<0.05). Source data are provided as a Source Data file.

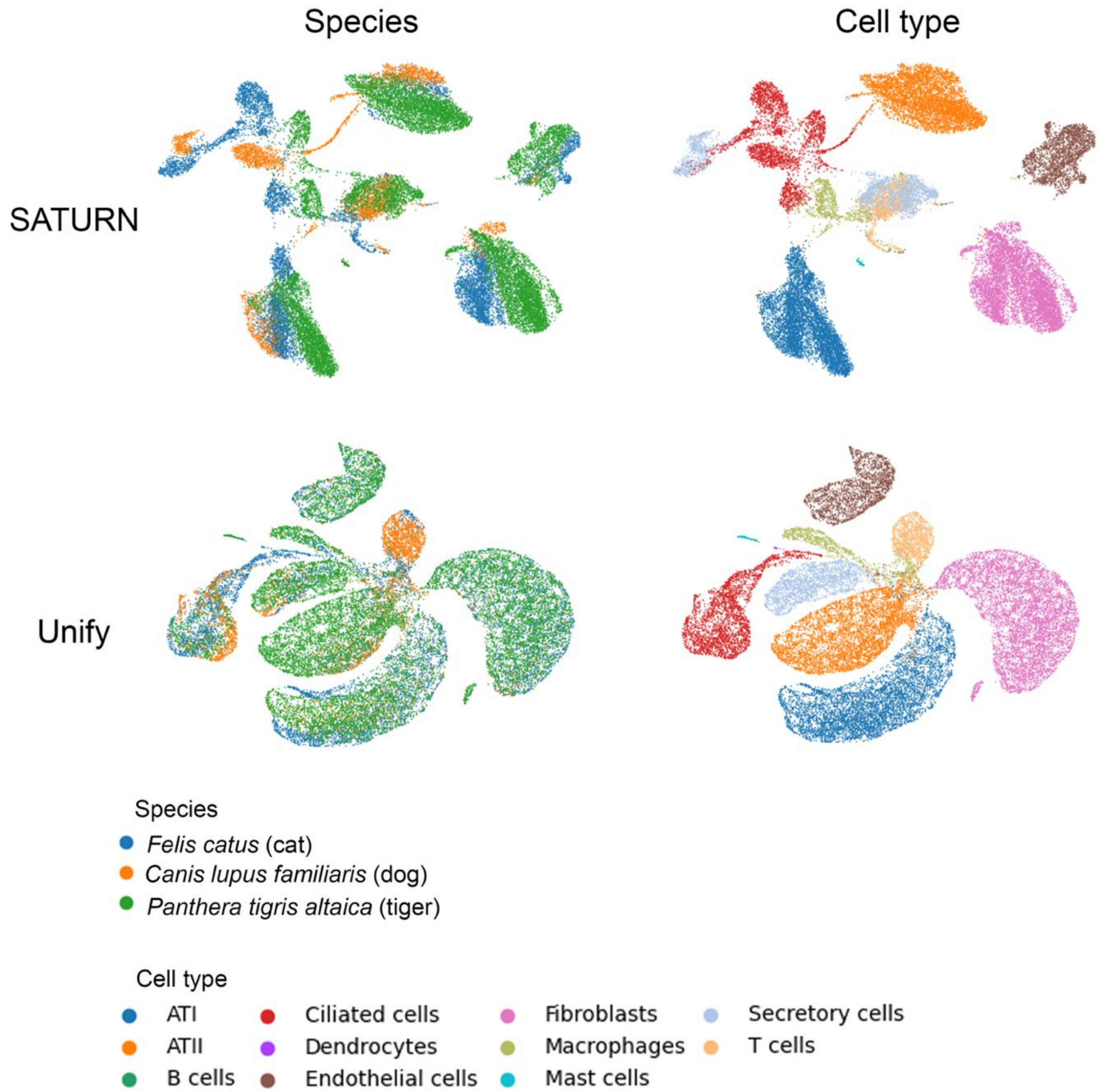

Supplementary Figure 25. UMAP layouts for the results from SATURN and Unify of integrating *Felis catus* (cat), *Canis lupus familiaris* (dog) and *Panthera tigris altaica* (tiger) datasets. SATURN fails to mix the species well for some cell types, such as Ciliated cells and Secretory cells.

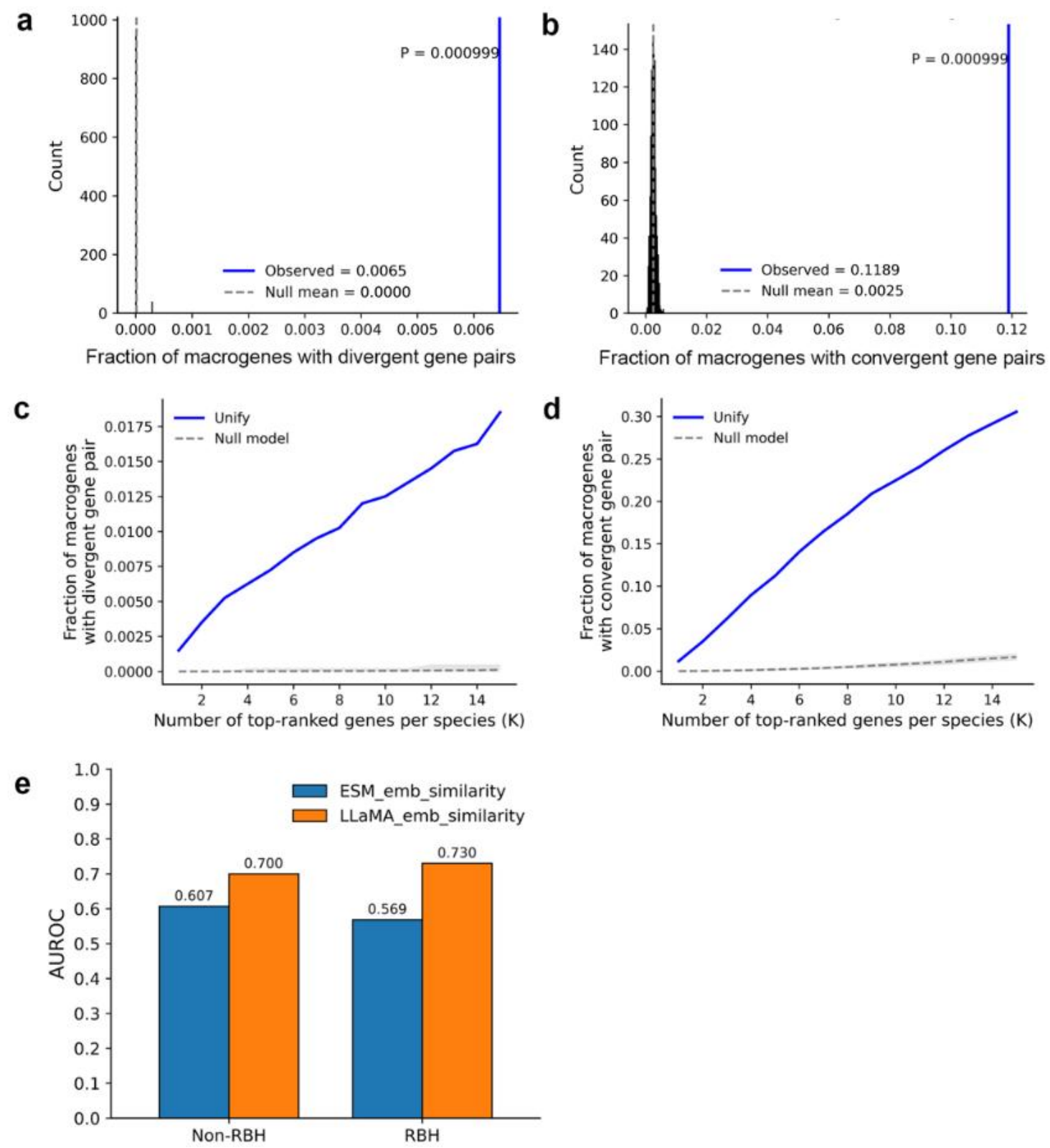

Supplementary Figure 26. Systematic evaluation of functional divergence and convergence captured by Unify macrogenes in the human–mouse comparison. (**a**) Permutation test (n=1000) for functional divergence, using the fraction of macrogenes containing at least one divergent gene pair as the test statistic. The histogram shows the null distribution obtained by randomizing gene-to-macrogene assignments while preserving the number of genes assigned to each macrogene in each species. The blue vertical line indicates the observed value and the dashed gray line indicates the null mean. (**b**) Permutation test (n=1000) for functional convergence, using the fraction of macrogenes containing at least one convergent gene pair as the test statistic, with the same permutation framework as in (**a**). In both analyses, observed values were significantly higher than expected under the null model. Panels (**a**) and (**b**) show enrichment results using the top-ranked gene from human and mouse in each macrogene. (**c,d**) Corresponding enrichment results obtained when the top K genes assigned to each macrogene were considered. (**e**) AUROC comparison of structural similarity (ESM embedding similarity) and functional text similarity (LLaMA embedding similarity) for distinguishing convergent and divergent relationships. For non-reciprocal best-hit (non-RBH which BLAST E-value > 0.05) pairs, AUROC was computed for identifying gene pairs with high functional similarity, corresponding to the ground-truth convergent set. For reciprocal best-hit (RBH) ortholog pairs, AUROC was computed for identifying orthologs with low functional similarity, corresponding to the ground-truth divergent set. Source data are provided as a Source Data file.

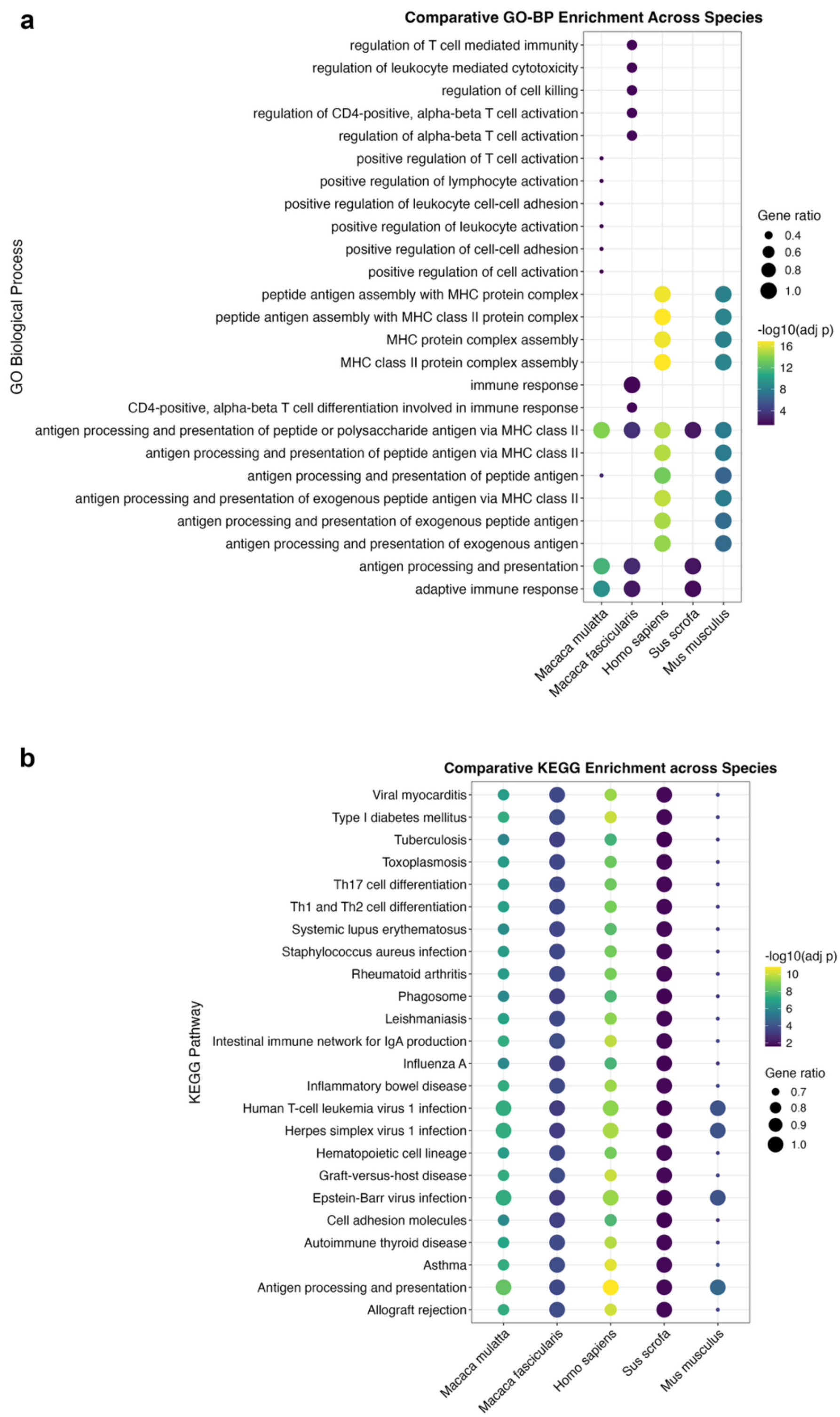

Supplementary Figure 27. Gene Ontology term enrichment and KEGG enrichment plots for the component genes of macrogene 857 for *Macaca mulatta, Macaca fascicularis, Homo sapiens, Sus scrofa* and *Mus musculus* in the AH atlas. (**a**) Gene ontology term enrichment plot. (**b**) KEGG enrichment plot. Source data are provided as a Source Data file.

a

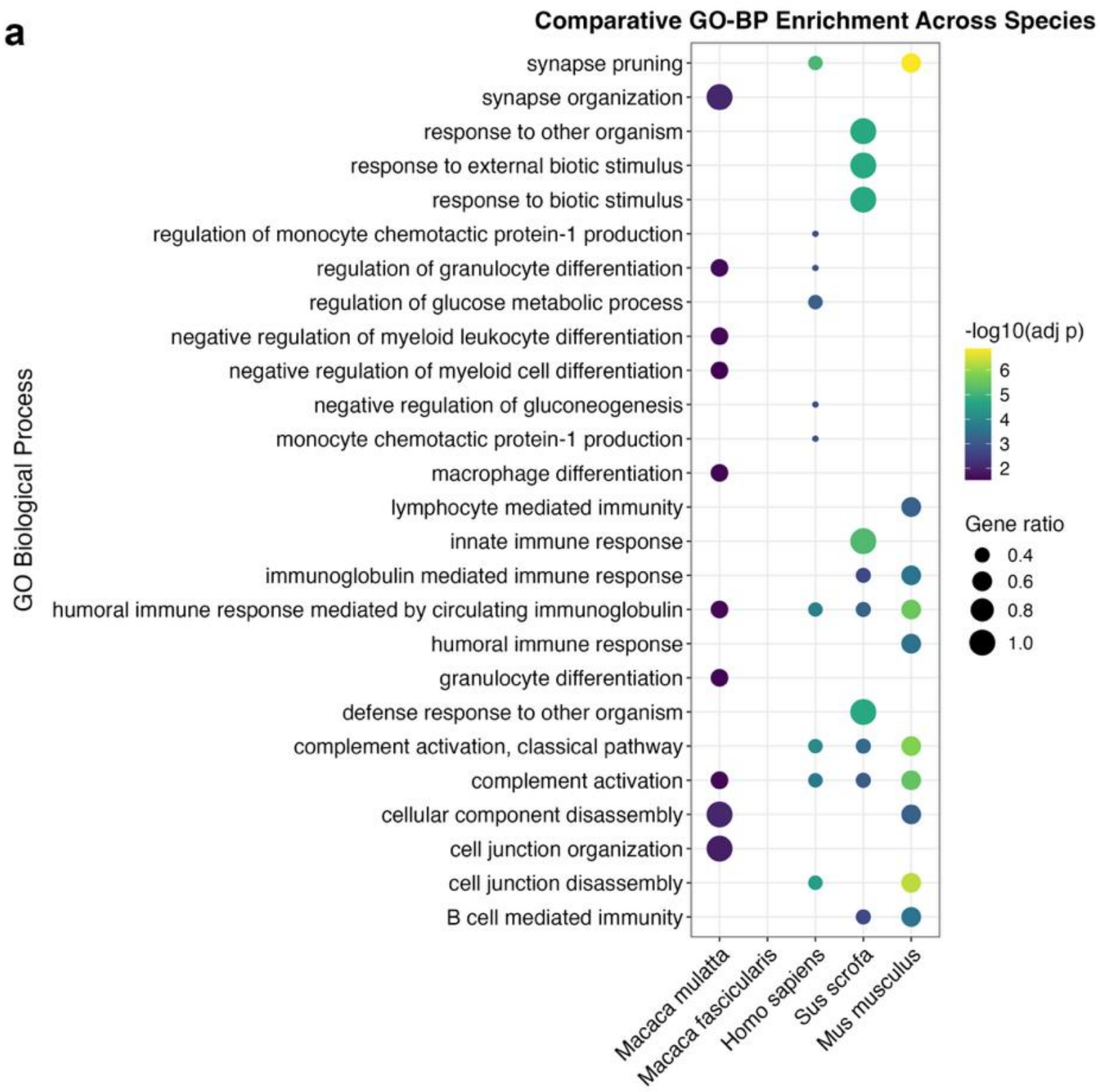

b

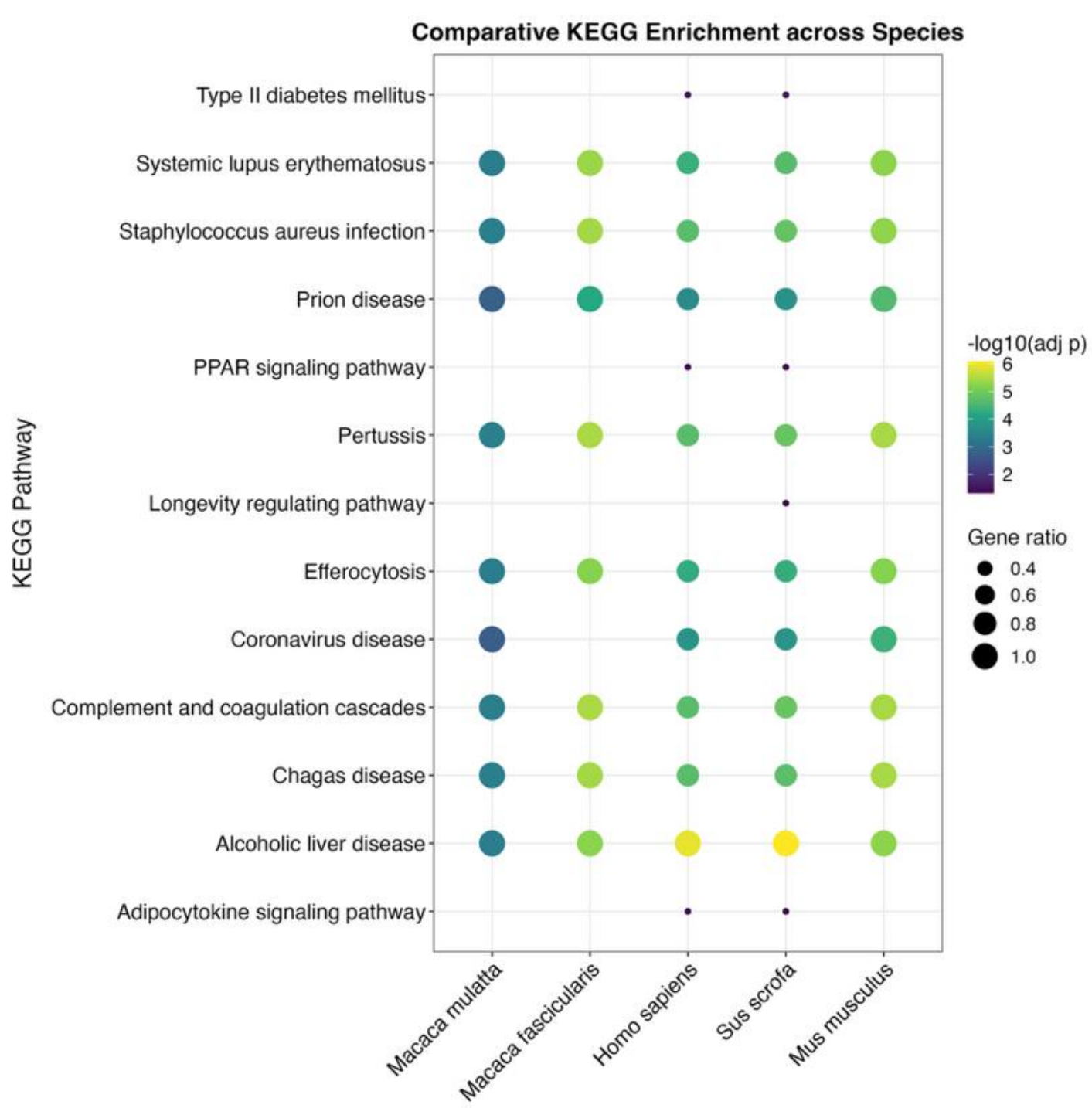

Supplementary Figure 28. Gene Ontology term enrichment and KEGG enrichment plots for the component genes of macrogene 838 for *Macaca mulatta, Macaca fascicularis, Homo sapiens, Sus scrofa* and *Mus musculus* in the AH atlas. (**a**) Gene ontology term enrichment plot. (**b**) KEGG enrichment plot. Source data are provided as a Source Data file.

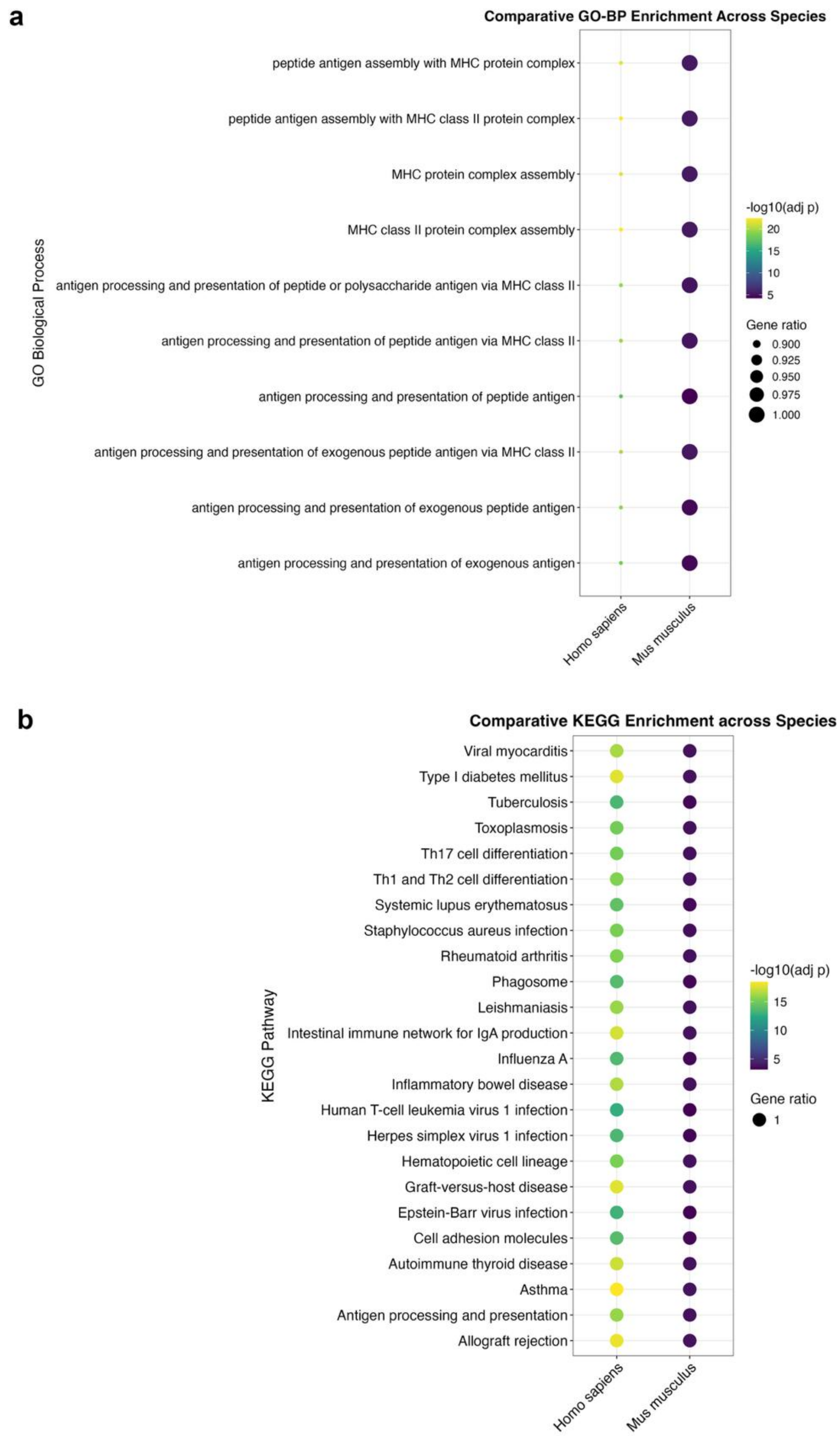

Supplementary Figure 29. Gene Ontology term enrichment and KEGG enrichment plots for the component genes of macrogene 2077 for *Macaca mulatta, Macaca fascicularis, Homo sapiens, Sus scrofa* and *Mus musculus* in the AH atlas. (**a**) Gene ontology term enrichment plot. (**b**) KEGG enrichment plot. Source data are provided as a Source Data file.

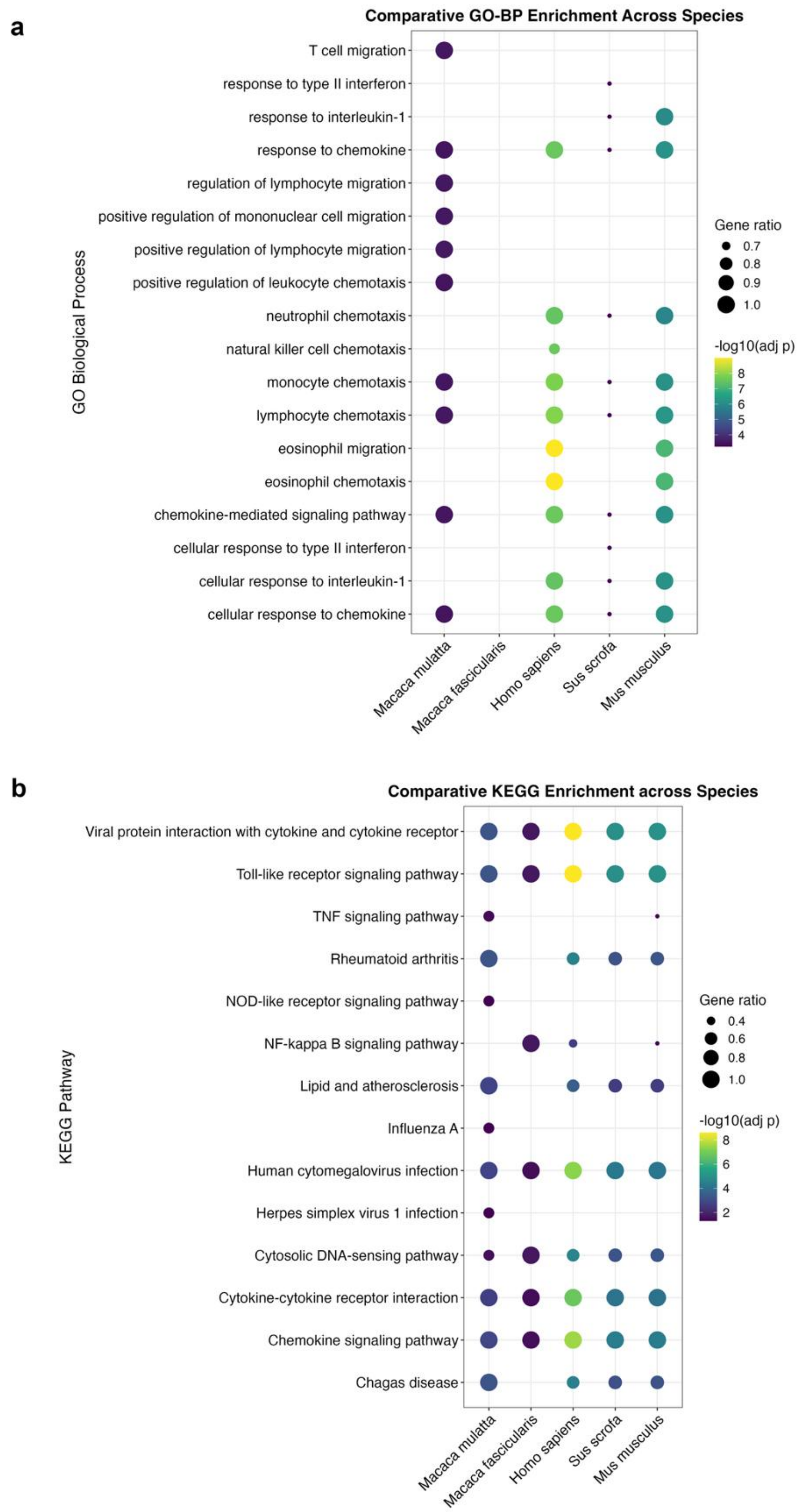

Supplementary Figure 30. Gene Ontology term enrichment and KEGG enrichment plots for the component genes of macrogene 1490 for *Macaca mulatta, Macaca fascicularis, Homo sapiens, Sus scrofa* and *Mus musculus* in the AH atlas. (**a**) Gene ontology term enrichment plot. (**b**) KEGG enrichment plot. Source data are provided as a Source Data file.

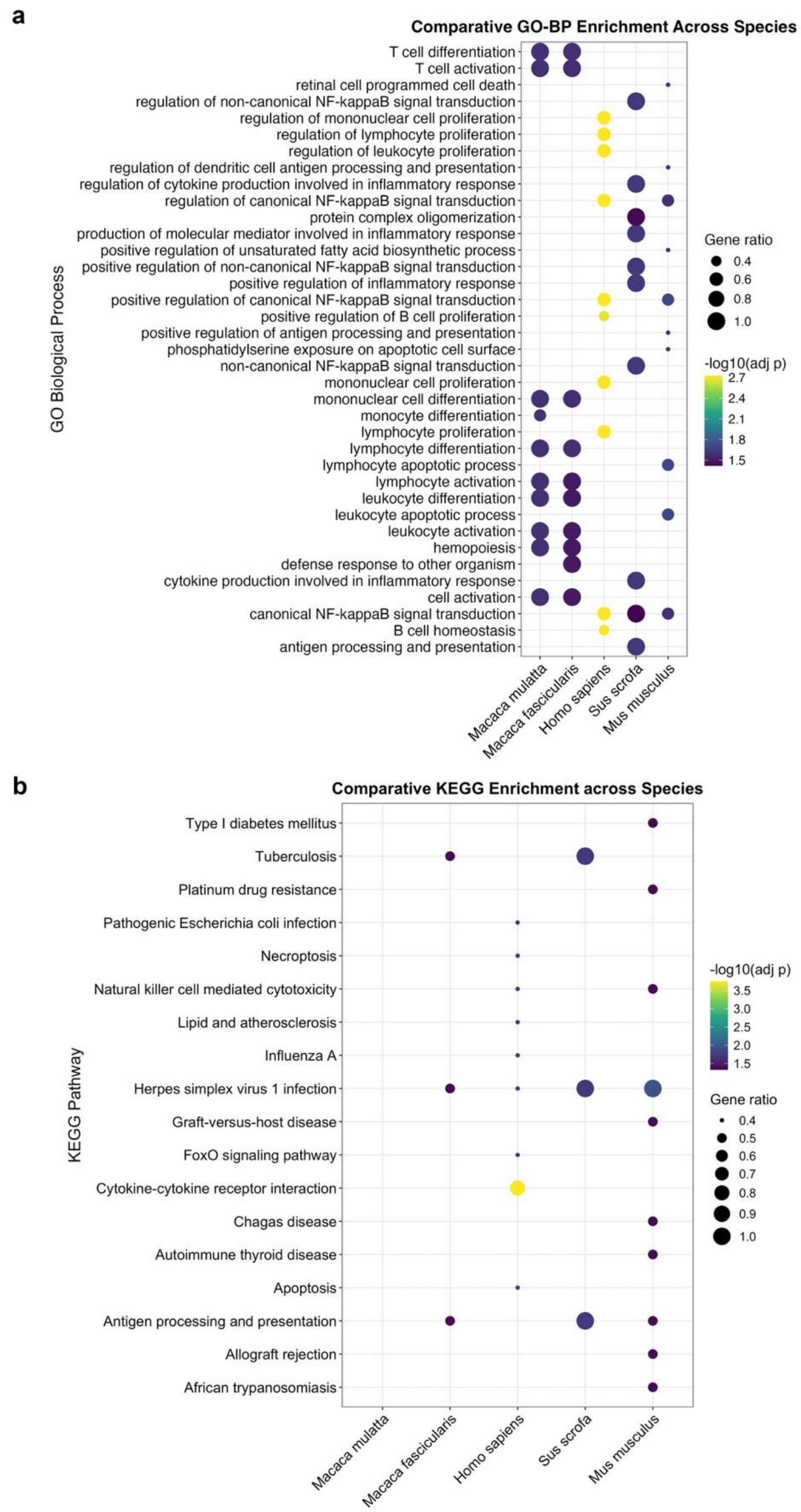

Supplementary Figure 31. Gene Ontology term enrichment and KEGG enrichment plots for the component genes of macrogene 141 for *Macaca mulatta, Macaca fascicularis, Homo sapiens, Sus scrofa* and *Mus musculus* in the AH atlas. (**a**) Gene ontology term enrichment plot. (**b**) KEGG enrichment plot. Source data are provided as a Source Data file.

a

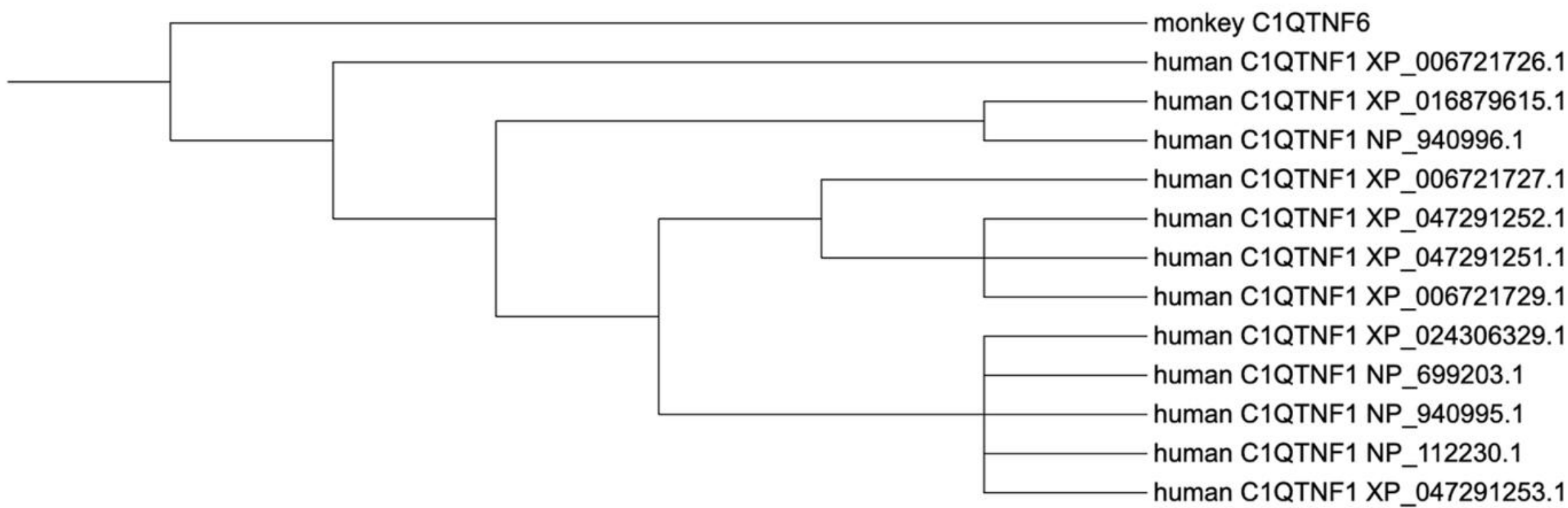

b

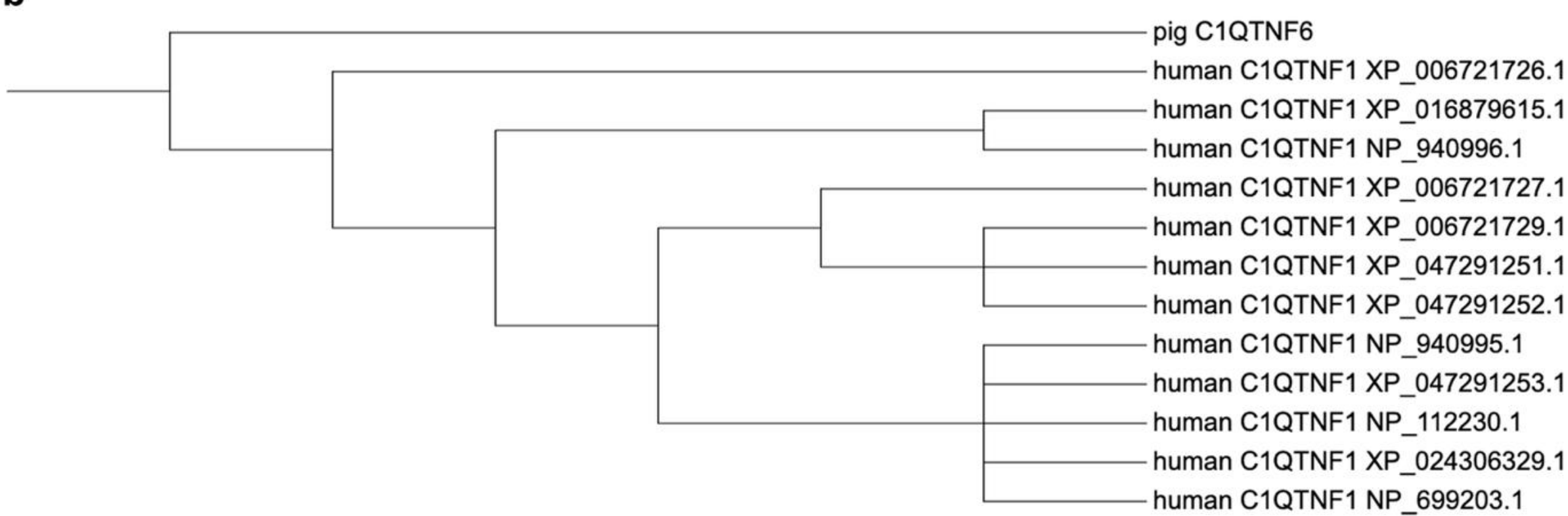

Supplementary Figure 32. Phylogenetic tree of the C1QTNF6 proteins sourced from human (*Homo sapiens*), monkey (*Macaca mulatta*) and pig (*Sus scrofa*). (**a**) Phylogenetic relationships between C1QTNF6 protein sourced from monkey and human. (**b**) Phylogenetic relationships between C1QTNF6 protein sourced from pig and human.

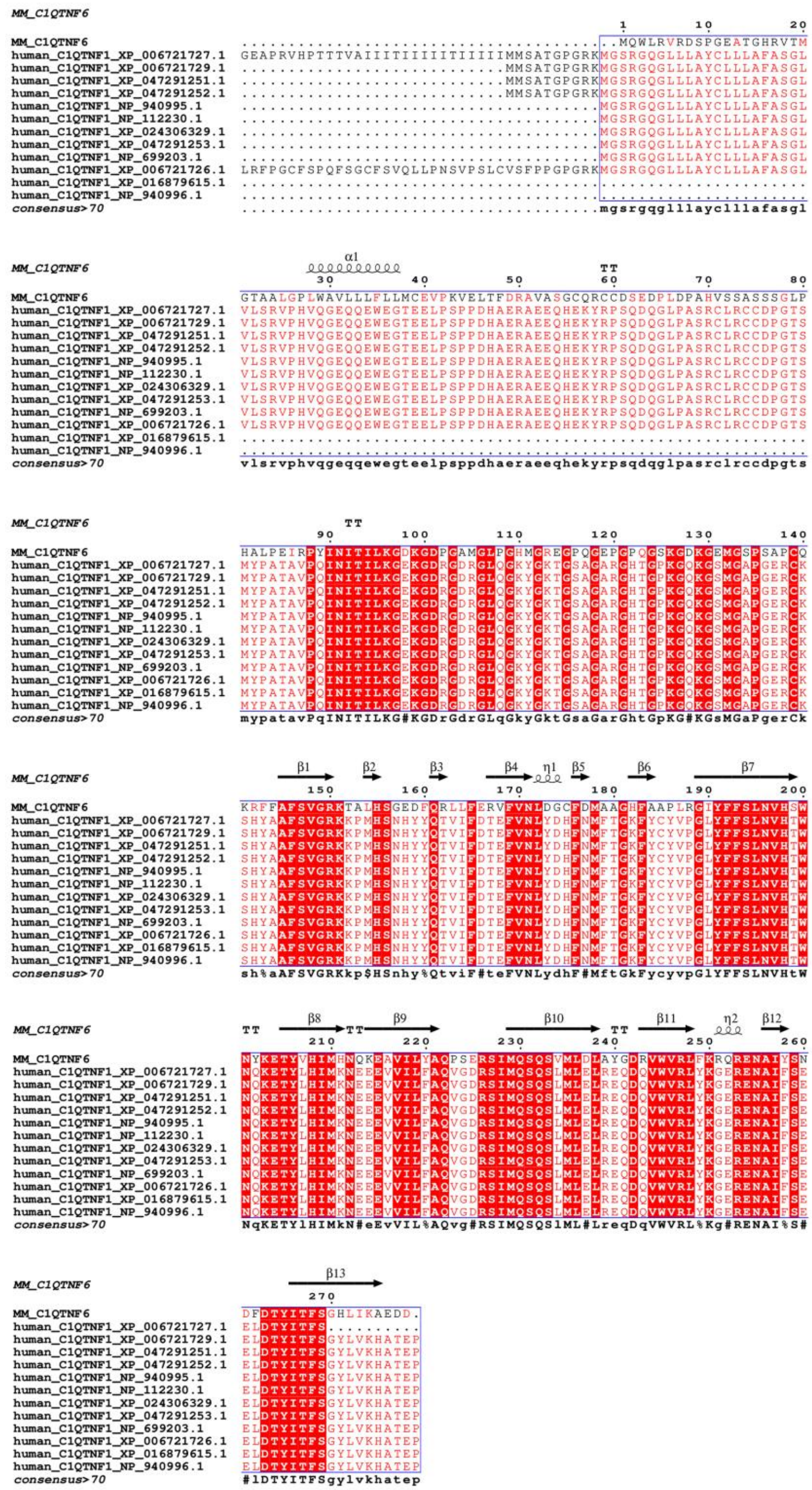

Supplementary Figure 33. Comparison of the sequence and the structure of the C1QTNF6 proteins sourced from monkey (*Macaca mulatta*) and human (*Homo sapiens*). η: secondary structure, 310 helix. Red box with white character means strict identity. Red characters mean similarity in a group: ISc (in-Group Score) > 0.7. Blue frame means similarity across groups: TSc (Total Score) > 0.7. TT: strict β-turns.

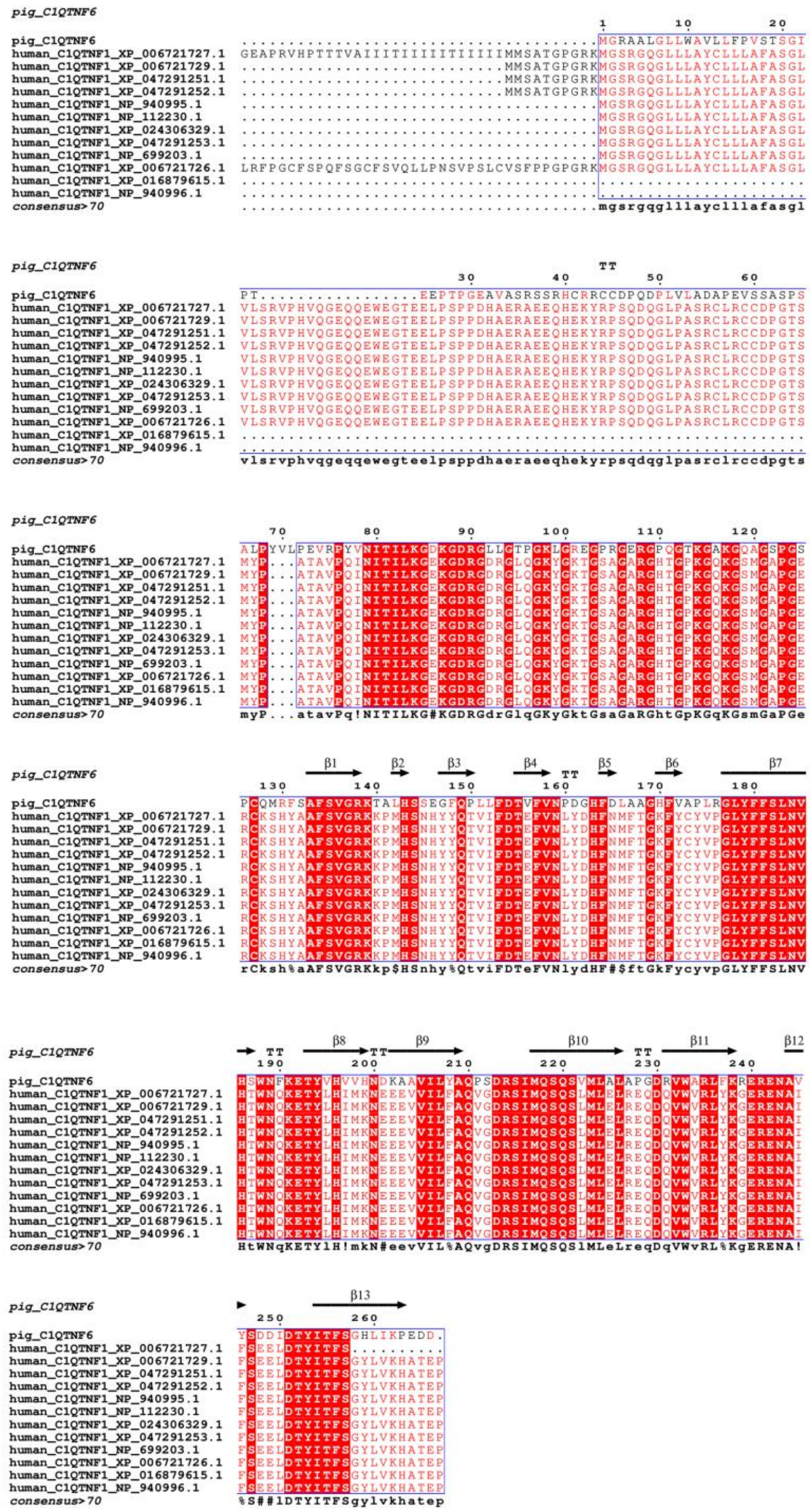

Supplementary Figure 34. Comparison of the sequence and the structure of the C1QTNF6 proteins sourced from pig (*Sus scrofa*) and human (*Homo sapiens*). η: secondary structure, 310 helix. Red box with white character means strict identity. Red characters mean similarity in a group: ISc (in-Group Score) > 0.7. Blue frame means similarity across groups: TSc (Total Score) > 0.7. TT: strict β-turns.

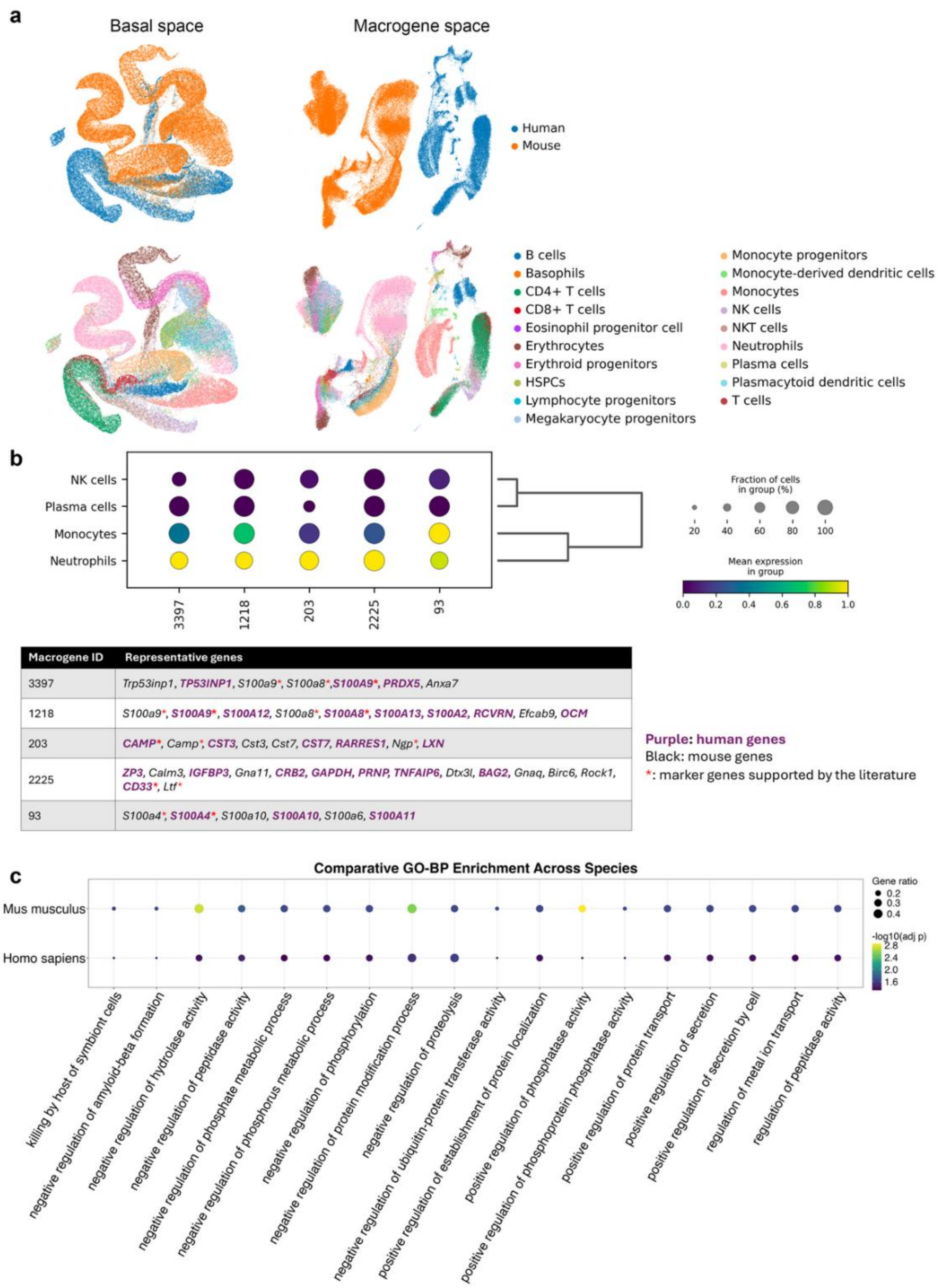

Macrogene ID	Representative genes
3397	Trp53inp1, TP53INP1, S100a9*, S100a8*, S100A9*, PRDX5, Anxa7
1218	S100a9*, S100A9*, S100A12, S100a8*, S100A8*, S100A13, S100A2, RCVRN, Efcab9, OCM
203	CAMP*, Camp*, CST3, Cst3, Cst7, CST7, RARRES1, Ngp*, LXN
2225	ZP3, Calm3, IGFBP3, Gna11, CRB2, GAPDH, PRNP, TNFAIP6, Dtx3l, BAG2, Gnaq, Birc6, Rock1, CD33*, Ltf*
93	S100a4*, S100A4*, S100a10, S100A10, S100a6, S100A11

Supplementary Figure 35. Integration results and the downstream differential macrogene analysis of immune datasets between human (*Homo sapiens*) and mouse (*Mus musculus*) using Unify. (**a**) UMAP plot of the macrogene space and the basal space after integration. (**b**) Dotplot of the expression level of the differential expressed macrogenes in macrophage (Upper) and the component genes of each differential expressed macrogenes (Lower). (**c**) Gene Ontology term enrichment plot of the component genes from human and mouse of macrogenes 2225. Source data are provided as a Source Data file.

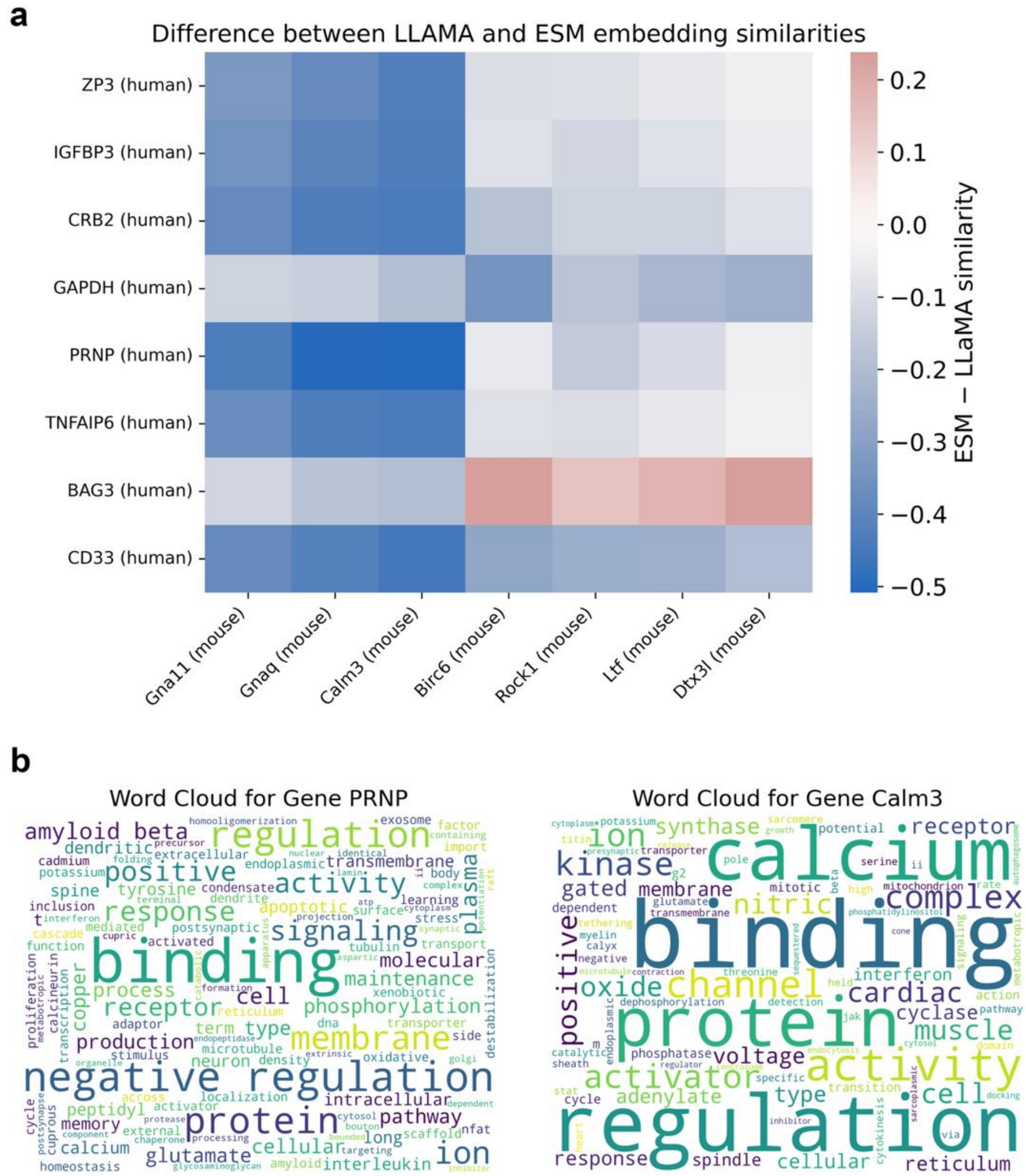

Supplementary Figure 36. Comparison of protein sequence and functional semantic similarities among selected macrogene component genes. (**a**) Heatmap of the ESM similarity and LLAMA similarity difference of the gene pairs within the component genes of the macrogene 2225 of human and mouse. (**b**) Word cloud of the Gene Ontology terms description of the human gene *PRNP* and mouse gene *Calm3*. Source data are provided as a Source Data file.

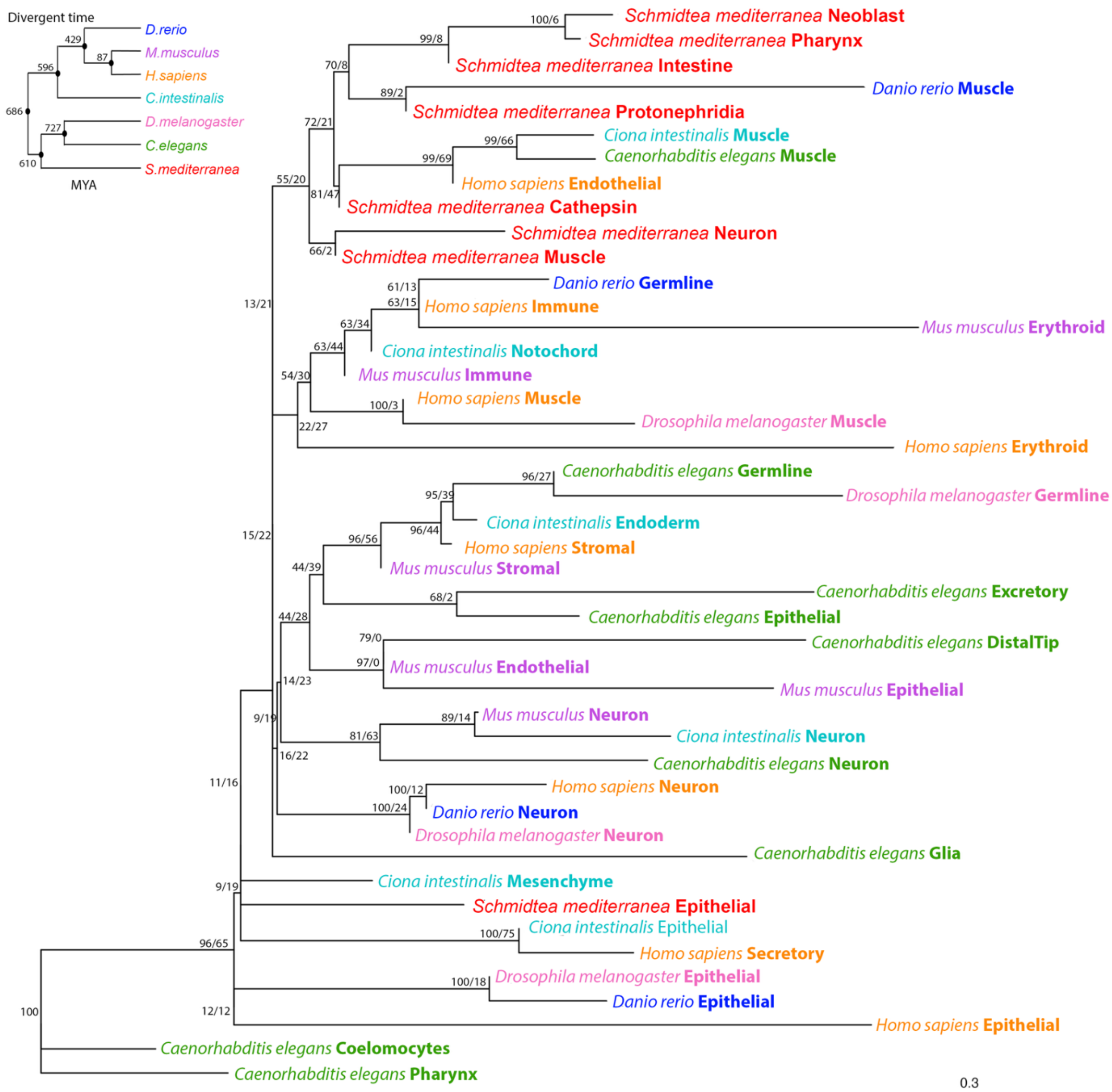

Supplementary Figure 37. Unrooted cell type tree for seven phylogenetically distant species (*Schmidtea mediterranea, Danio rerio, Ciona intestinalis, Mus musculus, Homo sapiens, Drosophila melanogaster*, and *Caenorhabditis elegans*) constructed from the integrated basal embedding generated by SATURN. Reproduced from Zhong *et al.*[18] by permission of Oxford University Press. Species and cell types are labeled at the tips. Node support values are printed as "jumble score/scjackknife score". MYA: million years ago.

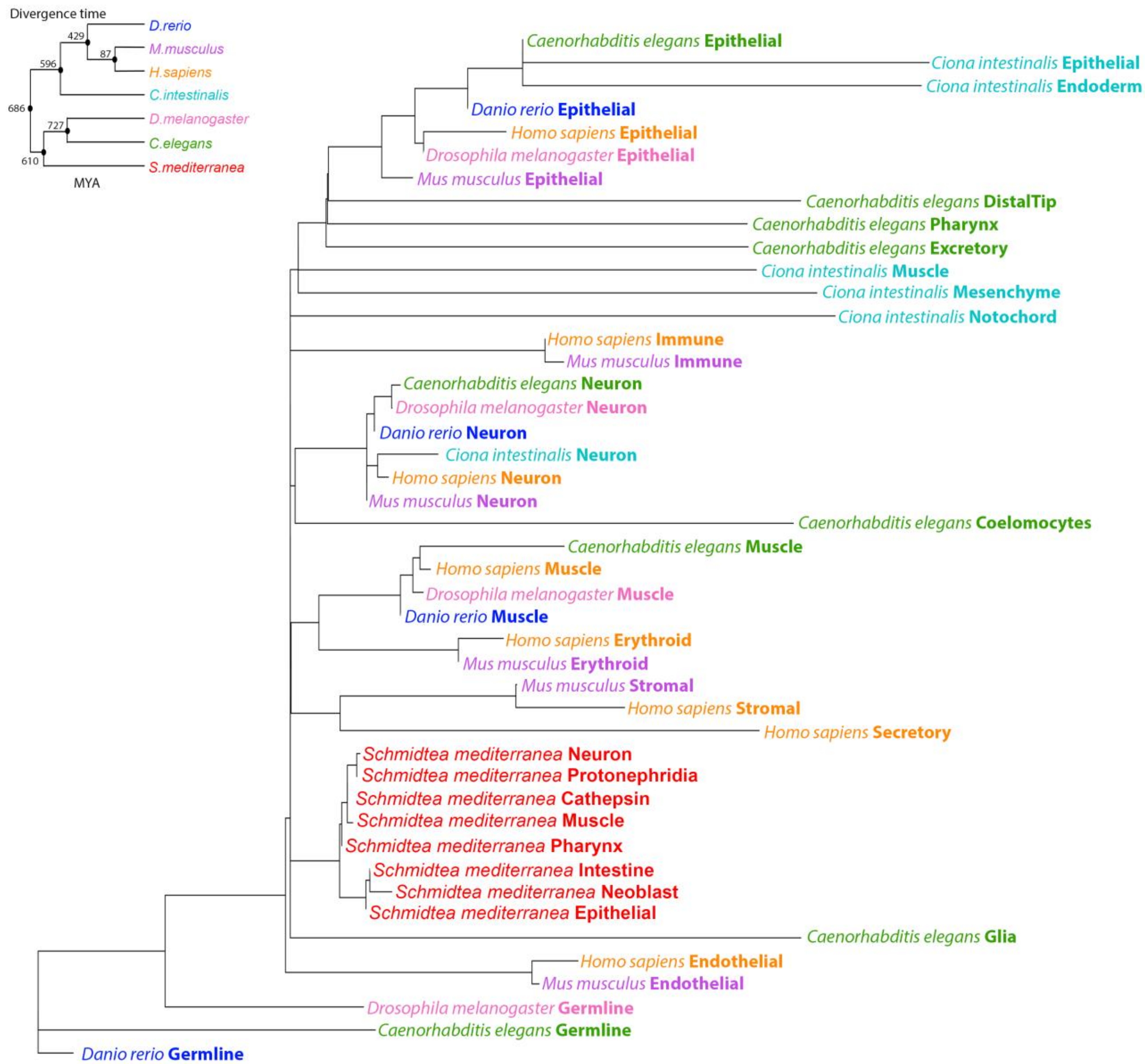

Supplementary Figure 38. Unrooted cell type tree across seven phylogenetically distant species (*Schmidtea mediterranea, Danio rerio, Ciona intestinalis, Mus musculus, Homo sapiens, Drosophila melanogaster and Caenorhabditis elegans*) constructed based on the meta-cell embeddings from Unify. MYA, million years ago.

Supplementary Table 1. Component genes of the differentially expressed macrogenes of macrophages.

Macrogene ID	Representative genes
857	*HLA-DOA (hs)*,*HLA-DQA2* (hs), *B2M* (hs), *HLA-DQA1*(hs), *HLA-DPA1* (hs), *HLA-DRA* (hs), *B2m* (mm), *H2-Aa* (mm), *H2-Oa* (mm), *MAMU-DRA* (mu), *MAMU-DOA* (mu), *HLA-DQA1*(mu), *MAMU-DQA1*(mu), *MAMU-DPA* (mu), *B2M* (mu), *LOC102144039* (mf), *LOC102141176* (mf), *LOC102136862* (mf), *SLA-DOA* (ss)
838	*C1QC** (hs), *C1QB** (hs), *C1QTNF3* (hs), *C1QTNF8* (hs), *CTHRC1* (hs), *C1QTNF1* (hs), *ADIPOQ* (hs), *C1QA** (hs), *C1qtnf3* (mm), *C1qa** (mm), *C1qb** (mm), *C1qtnf1*(mm), *C1qc** (mm), *C1QTNF1*(mu), *C1QC** (mu), *C1QTNF6* (mu), *C1QB** (mu), *C1QC** (mf), *C1QB** (mf), *C1QTNF1* (mf), *C1QTNF3* (mf), *C1QA** (mf), *C1QB** (ss), *ADIPOQ* (ss), *C1QC** (ss), *C1QA** (ss), *C1QTNF6* (ss), *C1QTNF1*(ss)
2077	*HLA-DQA2* (hs), *HLA-DPA1* (hs), *HLA-DQA1*(hs), *HLA-DRB1*(hs), *HLA-DRB5* (hs), *HLA-DRA* (hs), *HLA-DPB1* (hs), *HLA-DQB1* (hs), *HP* (hs), *H2-Ab1*(mm), *H2-Dma* (mm)
1490	*CCL3** (hs), *CCL3L3* (hs), *CCL5* (hs), *CCL4L2* (hs), *CCL4** (hs), *Ccl3** (mm), *Ccl5* (mm), *Ccl4** (mm), *CCL3** (mu), *CCL5* (mu), *CCL3** (mf), *LOC107127762* (mf), *LOC102136805* (mf), *CCL3L1* (ss), *CCL4** (ss), *CCL5* (ss)
141	*TNFSF10* (hs), *TNFSF8* (hs), *MCEMP1*(hs), *LSMEM1*(hs), *TNFSF13B* (hs), *FASLG* (hs), *CD74* (hs), *Mcemp1* (mm), *Fasl* (mm), *Lsmem1*(mm), *Cd74* (mm), *TNFSF8* (mu), *CD74* (mu), *MCEMP1*(mf), *TNFSF8* (mf),*CD74* (mf), *CD74* (ss), *LSMEM1* (ss)

* : Known marker genes of the macrophage. hs: human (*Homo sapiens*); ss: pig (*Sus scrofa*); mu: monkey (*Macaca mulatta*); mm (*Mus musculus*)

Supplementary references

1. UniProt Consortium, T. UniProt: the universal protein knowledgebase. *Nucleic Acids Research* **46**, 2699-2699 (2018).
2. The Gene Ontology resource: enriching a GOld mine. *Nucleic Acids Research* **49**, D325-D334 (2021).
3. Cantalapiedra, C.P., Hernández-Plaza, A., Letunic, I., Bork, P. & Huerta-Cepas, J. eggNOG-mapper v2: functional annotation, orthology assignments, and domain prediction at the metagenomic scale. *Molecular Biology and Evolution* **38**, 5825-5829 (2021).
4. Huerta-Cepas, J. *et al*. eggNOG 5.0: a hierarchical, functionally and phylogenetically annotated orthology resource based on 5090 organisms and 2502 viruses. *Nucleic Acids Research* **47**, D309-D314 (2019).
5. Rosen, Y. *et al*. Toward universal cell embeddings: integrating single-cell RNA-seq datasets across species with SATURN. *Nature Methods* **21**, 1492-1500 (2024).
6. Tarashansky, A.J. *et al*. Mapping single-cell atlases throughout Metazoa unravels cell type evolution. *eLife* **10**, e66747 (2021).
7. Haghverdi, L., Lun, A.T., Morgan, M.D. & Marioni, J.C. Batch effects in single-cell RNA-sequencing data are corrected by matching mutual nearest neighbors. *Nature Biotechnology* **36**, 421-427 (2018).
8. Korsunsky, I. *et al*. Fast, sensitive and accurate integration of single-cell data with Harmony. *Nature Methods* **16**, 1289-1296 (2019).
9. Lotfollahi, M., Naghipourfar, M., Theis, F.J. & Wolf, F.A. Conditional out-of-distribution generation for unpaired data using transfer VAE. *Bioinformatics* **36**, i610-i617 (2020).
10. Lopez, R., Regier, J., Cole, M.B., Jordan, M.I. & Yosef, N. Deep generative modeling for single-cell transcriptomics. *Nature Methods* **15**, 1053-1058 (2018).
11. Polański, K. *et al*. BBKNN: fast batch alignment of single cell transcriptomes. *Bioinformatics* **36**, 964-965 (2020).
12. Hie, B., Bryson, B. & Berger, B. Efficient integration of heterogeneous single-cell transcriptomes using Scanorama. *Nature Biotechnology* **37**, 685-691 (2019).
13. Hao, Y. *et al*. Integrated analysis of multimodal single-cell data. *Cell* **184**, 3573-3587. e3529 (2021).
14. Luecken, M.D. *et al*. Benchmarking atlas-level data integration in single-cell genomics. *Nature Methods* **19**, 41-50 (2022).
15. Wolf, F.A., Angerer, P. & Theis, F.J. SCANPY: large-scale single-cell gene expression data analysis. *Genome Biology* **19**, 15 (2018).
16. Mah, J.L. & Dunn, C.W. Cell type evolution reconstruction across species through cell phylogenies of single-cell RNA sequencing data. *Nature Ecology & Evolution* **8**, 325-338 (2024).
17. Robinson, D.F. & Foulds, L.R. Comparison of phylogenetic trees. *Mathematical Biosciences* **53**, 131-147 (1981).
18. Zhong, H. *et al*. Benchmarking cross-species single-cell RNA-seq data integration methods: towards a cell type tree of life. *Nucleic Acids Research* **53**, gkae1316 (2025).
