# Supplementary material for: Unify learns cellular evolution with universal multimodal embeddings
Source: Nat Commun. 2026 Jul 31;17:9290. doi: 10.1038/s41467-026-76230-y (PMC13527131; doi:10.1038/s41467-026-76230-y)
Supplement: Supplementary file 3 [file 41467_2026_76230_MOESM2_ESM.pdf]

nature portfolio

Peer Review File

Unify Learns Cellular Evolution with Universal Multimodal Embeddings

Corresponding Author: Professor Manuel Aranda

This file contains all reviewer reports in order by version, followed by all author rebuttals in order by version.

Version 0:

Reviewer comments:

Reviewer #1

(Remarks to the Author)
The paper “Unify: Learning Cellular Evolution with Universal Multimodal Embeddings” presents a transfer learning framework designed to integrate single-cell RNA-sequencing data across species. The method introduces a novel multi-modal "macrogene" representation by combining protein embeddings (ESM-2) with functional text embeddings (LLaMA-2). Unify employs an adversarial autoencoder guided by a Pareto-optimal multi-task learning strategy (MGDA-UB) to balance reconstruction, batch correction, and biological conservation. The authors benchmark Unify against state-of-the-art methods and demonstrate its utility in identifying differentially expressed macrogenes, predicting cross-species perturbation responses, and reconstructing a 700-million-year cell-type evolutionary tree.
While the integration of multimodal embeddings and Pareto optimization is conceptually interesting, the manuscript lacks critical rigorous validation of these components. There are substantial concerns regarding the fairness of benchmarks, the necessity of complex architectural choices, and the robustness of the evaluation datasets. Major and minor points are listed as below:

Major points:
1. In Figure 4B–F, Unify-basal often performs on par with or even slightly better than Unify-macrogene, especially for the “All genes” setting. Since the text emphasizes macrogenes as a core innovation, it would be helpful if the authors could clarify in more detail what additional benefits Unify-macrogene provides beyond Unify-basal in this prediction task (e.g., interpretability, evolutionary analyses), and whether the performance differences between the two variants are statistically significant.
2. While the choice of ESM and LLaMA is reasonable and well-motivated, the manuscript would benefit from a brief discussion of why these particular foundational models were selected, and whether alternative protein or text models (e.g., ProtT5, ProtTrans, PubMedBERT, Bloom, or other LLMs) might yield similar or improved performance. Since multimodal macrogene construction is a central innovation of Unify, even a small benchmark or an ablation comparing different foundational models—or a discussion explaining why such benchmarking is unnecessary—would help strengthen the rationale and reproducibility of this component.
3. Absence of ablation studies: There is a complete absence of ablation studies to justify the model's complexity. It is unclear whether the reported performance gains stem from the novel multimodal embeddings, the specific optimization algorithm, or simply the supervised nature of the training. The authors must deconstruct their model to validate the necessity of each component:
a) Pareto Optimization: The authors claim MGDA-UB is superior to fixed weighting. An ablation comparison of Unify (MGDA-UB) vs. Unify (Fixed Weights/Grid Search) is required to prove the robustness of this contribution.
b) Cell-type Supervision: The model uses a cell-type classifier loss. The authors should test the model without this supervision signal to determine its impact on bio-conservation scores.
c) Multimodality: The authors must demonstrate that the inclusion of text-based embeddings provides a statistically significant performance gain over using protein embeddings alone. A comparison of Unify (Full) vs. Unify (Only ESM-2) vs. Unify (Only LLaMA-2) is essential. Without this, it is difficult to conclude that the LLM contributes meaningfully to performance, and the inclusion may appear unnecessary.
4. Insufficient benchmark datasets: The benchmarking section relies on a very limited set of species pairs—only one pair per phylogenetic level (e.g., one cross-genus pair, one cross-family pair). This is insufficient to establish the stability and generalizability of the method. The authors should include additional cross-genus and cross-family pairs to demonstrate that Unify's performance is consistent and statistically significant compared to other tools across varied biological contexts.

5. Unfair comparison regarding supervision: Unify explicitly utilizes cell type annotation information during training, whereas many competing methods are unsupervised. For a fair evaluation, the benchmark results should clearly distinguish between methods that require cell type labels (e.g., Unify, SATURN) and those that do not.
6. Dependency on cell type labels: It is unclear how Unify handles scenarios where cell type labels are missing or inconsistent. The authors should evaluate: 1) Performance when one or both datasets lack cell type annotations. 2) Performance when labels are not aligned or comparable. This is critical for real-world applications where ground-truth cross-species annotations are often unavailable.
7. Discrepancy in Trajectory Conservation: In Supplementary Table 2, the "Trajectory conservation" scores for Unify in Cross-genus and Cross-family tasks appear significantly lower or inconsistent compared to other metrics or tools. The authors should explain this phenomenon in the Results or Discussion section. Does the method trade off trajectory preservation for cluster distinctness?
8. Computational resource reporting: Given the use of large foundation models (ESM-2, LLaMA-2) and adversarial training, the computational cost is a concern. The authors should provide detailed statistics on the hardware used (GPU/CPU type), memory/VRAM usage, and runtime for the major experiments.

Minor points:
1. Line 122 refers to 'Figure 1A', but only 'Figure 1' appears in the manuscript.
2. In line 133, MGDA-UB appears for the first time in the Results section, but its full name is only provided later in the Methods (line 544). Readers who are not familiar with this algorithm may find it confusing. I suggest adding the full term when MGDA-UB is first introduced.
3. Authors may cite previous benchmark studies to justify the use of a 40% batch-correction and 60% bio-conservation weighting scheme, but the manuscript does not explain why this ratio is appropriate for the present study. Given that the overall score is central to method ranking, it would be helpful for the authors to provide a brief rationale or sensitivity analysis showing that the conclusions are robust to alternative weighting choices.
4. Line 228 contains a typographical error: “marcogenes” should be corrected to “macrogenes.”
5. In Figure 3C, the red stars marking known marker genes are not clearly visible for some species. Improving their visibility would enhance the readability of the figure.
6. Authors refer to “top DEGs” when evaluating perturbation prediction performance (e.g., Figure 4D), but the manuscript does not define how “top DEGs” are selected (e.g., based on log fold change, adjusted p-value, fixed number of genes, or percentage thresholds). Since these definitions directly influence hit rate and direction accuracy, a clear explanation in the Methods section would improve reproducibility.

(Remarks on code availability)

Reviewer #2

(Remarks to the Author)
Zhong et al. proposed a computational method, Unify, to align cells across species through macrogenes. The idea is an extension of a previous framework, SATURN, in which macrogenes are initiated through structural embeddings; Unify further incorporates functional gene embeddings to initiate macrogenes. Unify is claimed to perform better at cell alignment, characterizing evolutionary functional divergence, predicting perturbation effects across species, and inferring cell-type trees. Although these applications are important to the field, the description of the method is not clear, and the results are not convincing in the following areas:

1. The notation in Line 408 is not shown in the pdf format.

2. Some notations are not intuitive – e.g., on Line 466, species is indicated as Si, but then Ei is introduced as average protein embedding. Should it be E_Si? What is it averaged across?

3. Regarding cell alignment performance comparison, it is not fair as Unify already has seen cell type labels, thus it is not surprising that it outperforms others at aligning cell types. The question is, if cell type labels and matching across species are already given, why do we need to align single cells across species?

4. How were hyperparameters tuned for other methods?

5. Regarding functional divergence and convergence, only case studies are shown, and a systematic evaluation is missing.

6. Figure 3E and 3F: There are several dotted lines that are not annotated. Also, it is unclear how the similarity score between ESM and LLaMA is calculated. Intuitively, since these two embeddings are generated separately, a direct correlation analysis between them does not make sense due to the separate coordinates of these two embeddings.

7. Regarding perturbation effect prediction, it is not clear how and why the linear transformation of macrogenes works. For example, for the upper right box in Figure 4A, I am guessing the addition and subtraction are done on the cell by macrogene space, but then why is the subtraction done when there is no cell correspondence across species and perturbation status?

8. The authors claim “all genes” in the figure legend, but indicated that they tested on “highly variable genes” on Line 298 for those figures. It would be more practically useful if the experiments were truly done on all genes instead of highly variable

genes.

9. It is also unclear whether the difference between Unify and other methods is statistically significant.

10. Similarly, regarding cell type tree construction, it is not surprising that Unify performs well, given that it has already seen the cell type mapping across species. It would be useful if Unify can identify some new connections that are not indicated in the original mapping.

11. Ablation study on whether adding functional embeddings or structural embeddings improves the performance is needed.

(Remarks on code availability)

Reviewer #3

(Remarks to the Author)
In this manuscript, Zhong et al. present Unify, a deep learning framework designed for integrating single-cell RNA-seq data across species. The authors introduce universal multimodal embeddings that incorporate gene expression, structural features, and functional annotations derived from LLMs. They also employ MGDA-UB to automatically correct batch effects with biological conservation, eliminating the need for manual weight adjustment. The study shows that Unify outperforms existing methods across various evolutionary distances, particularly excelling in comparisons between distant species where other methods struggle. Furthermore, the authors applied Unify to predict cellular responses to perturbations across species. However, several major and minor concerns need to be addressed.

Major
1. In the “Cell type tree construction” section, the strategy of randomly sampling “one cell per cell type per species” is mentioned. Relying on a single cell to represent an entire cell type for phylogenetic analysis is statistically problematic given the sparsity and noise inherent in scRNA-seq data (e.g., dropouts). Please perform a sensitivity analysis by increasing the number of sampled cells (e.g., sample 10, 50, 100 cells per type and calculate a consensus) or by using “pseudobulks/centroids” to represent cell types. Demonstrate that the tree topology is consistent under these more robust representations would significantly strengthen the biological validity of your conclusions regarding cell type evolution.
2. In the “Benchmark evaluations”, it is noted that Unify uses all genes, whereas baseline methods (such as scGen or scVI) are limited to one-to-one orthologs. Although leveraging all genes is a methodological advantage of Unify, it introduces a confusion in the evaluation: does the observed performance gain come from the Unify architecture itself or simply from having access to more input features?
3. The paper claims significant benefits from ESM-2 and LLaMA-2 embeddings. To quantify this benefit, an ablation study is necessary. Please report Unify’s performance (e.g., bio-conservation scores) when: (a) Only sequence homology/structural embeddings are used; (b) Only LLM-derived functional text embeddings are used.

Minor
1. The framework incorporates complex components, such as LLMs and adversarial training with MGDA-UB. Please provide a table comparing the runtime and peak memory usage (GPU VRAM) of Unify versus major competitors (e.g., SATURN and SAMap) on datasets of varying sizes. This will useful to assess the practical accessibility of the tool.
2. The manuscript reports predicting perturbation responses (e.g., Mouse to Human) and mentions validation against “experimentally measured data”. Please explicitly describe the data-splitting strategy. Was the target species' perturbed data completely excluded during the training phase (zero-shot prediction)? Clarification is needed to ensure that no data leakage occurred, as this is a common concern in generative integration tasks.
3. In line 112, the text mentioned ‘Figure 1A’. However, Figure 1 does not include panel A. Please carefully review and correct the main text and figures.
4. In Figure 2A, please include a legend explaining the meaning of the dark and light colors.
5. In line 408, the characters are not displayed correctly.

(Remarks on code availability)
The authors refer to a GitHub repository. Please ensure that it includes a “minimal reproducible example” (a toy dataset) accompanied with a clear tutorial. This will enable reviewers and users to quickly verify installation and basic runs without downloading large datasets.

Reviewer #4

(Remarks to the Author)
I co-reviewed this manuscript with one of the reviewers who provided the listed reports. This is part of the Nature Communications initiative to facilitate training in peer review and to provide appropriate recognition for Early Career Researchers who co-review manuscripts.

(Remarks on code availability)

Version 1:

Reviewer comments:

Reviewer #1

(Remarks to the Author)
The authors have been thorough in their revisions, and I appreciate the additional analyses provided. The scientific concerns I previously raised have been largely resolved, and the model's robustness is now much more transparent. The study is ready for publication, provided the following minor points are addressed:

1. A correction is needed for the scientific name. The manuscript uses Macaque mulatta, which should be Macaca mulatta.
2. The citation for scGen needs to be standardized. It is currently linked to reference 17 (the transfer VAE paper) in some sections, while correctly attributed to reference 26 elsewhere. Please ensure all instances point to the correct source.
3. There is a persistent typo in the Methods section where SATURN is written as SATRUN. Please correct this.

(Remarks on code availability)

Reviewer #2

(Remarks to the Author)
Thank you for addressing many of the previous comments. I have one additional comment regarding the responses:

In Line 802, the authors state that "In all ablation analyses, model parameters and training settings were kept identical to those of the Unify model, and only the component under investigation was modified." It would be helpful for the authors to clarify which model parameters and training settings were tuned. In addition, could the authors comment on how the results might change if the ablation models were independently tuned, rather than using the identical settings from the full Unify model?

This is also related to my original question regarding hyperparameter tuning for competing methods. In practice, users would likely either tune hyperparameters for all methods or use default settings for all methods. Therefore, if Unify benefits from tuned hyperparameters, a fair comparison would require a comparable level of hyperparameter tuning for the competing methods as well. Clarifying this point would help support the claimed robustness, generalizability, and performance advantages of the proposed method on new datasets.

(Remarks on code availability)

Reviewer #3

(Remarks to the Author)
Overall,authors have addressed all my concerns.

(Remarks on code availability)

Version 2:

Reviewer comments:

Reviewer #2

(Remarks to the Author)
The authors have addressed all my concerns.

(Remarks on code availability)

Open Access This Peer Review File is licensed under a Creative Commons Attribution 4.0 International License, which permits use, sharing, adaptation, distribution and reproduction in any medium or format, as long as you give appropriate credit to the original author(s) and the source, provide a link to the Creative Commons license, and indicate if changes were made.
In cases where reviewers are anonymous, credit should be given to 'Anonymous Referee' and the source.
The images or other third party material in this Peer Review File are included in the article's Creative Commons license, unless indicated otherwise in a credit line to the material. If material is not included in the article's Creative Commons license and your intended use is not permitted by statutory regulation or exceeds the permitted use, you will need to obtain permission directly from the copyright holder.
To view a copy of this license, visit https://creativecommons.org/licenses/by/4.0/

Table of Contents

Overview: ***... 1***
Response to Reviewer 1: ***.. 4***
Response to Reviewer 2: ***.. 25***
Response to Reviewer 3: ***.. 40***
Reference ***... 48***

Overview:

We sincerely thank the reviewers for their careful reading of our manuscript and for the many insightful and constructive comments. Their feedback has been extremely valuable in strengthening both the rigor and clarity of the study. In response, we have substantially revised the manuscript, expanded the benchmarking analyses, added multiple new ablation and sensitivity studies, clarified key methodological details, and updated the main and supplementary figures, tables, and notes accordingly.

The revisions are summarized as follows:

1. **Expansion and clarification of the benchmarking framework.**

Additional benchmark datasets were included for each phylogenetic integration category, together with statistical comparisons across methods. We also clarified the role of supervision in benchmark setting by explicitly indicating whether each benchmarked method is supervised (cell type label-informed) or unsupervised. We added additional justification for the weighting scheme used to calculate the overall benchmark score, together with a sensitivity analysis across alternative weighting ratios.

2. **Addition of multiple ablation studies to dissect the contribution of key model components.**

These now include ablations for:

(i) Multimodal versus single-modality embeddings (Unify-full, Unify-ESM, and Unify-LLaMA).

(ii) Pareto-optimal training versus fixed-weight optimization.

(iii) Alternative protein (ProtT5, ProstT5) and text foundation models (PubMedBERT and BLOOM).

(iv) Varying levels of cell-type supervision, comparing fully supervised, semi-supervised (replacing one species' labels with unsupervised clusters), and completely unsupervised training regimes.

(v) Unify taking all genes as input versus Unify taking one2one orthologous genes as input.

3. **Substantial revision of the perturbation prediction analyses.**

The previous “all genes” setting was replaced with the true full available gene set rather than 8,000 highly variable genes. We also added formal statistical comparisons between the two Unify variants and the competing methods and clarified the zero-shot evaluation setup to remove any ambiguity regarding data leakage.

4. **Addition of a systematic evaluation of functional divergence and convergence.**

In response to concerns that the original manuscript relied too heavily on illustrative case studies, we added a quantitative, systematic analysis of functional divergence and convergence, along with corresponding revisions to the Results and Methods sections.

5. **Strengthening of the cell-type tree analysis.**

We revised the description of tree construction, added sensitivity analyses comparing alternative cell representations, and expanded the Results section on the biologically informative connections identified by Unify relative to SATURN.

6. **Reporting of computational scalability and resource usage.**

We added supplementary benchmarking of runtime and memory usage across methods and dataset sizes.

7. **Clarification of several methodological details throughout the manuscript.**

These revisions include clearer definitions of notation and symbols, an explanation of hyperparameter tuning for baseline methods, clarification of the linear transformation used in perturbation prediction, and explanations of the dotted lines and similarity calculations in Figure 3e-f.

8. **Correction of minor textual, notation, and figure issues.**

These include correction of figure references (for example, “Figure 1A” to “Figure 1”), expansion of the full term for MGDA-UB at first mention, correction of typographical errors, improved visibility of annotations in Figure 3c, restoration of missing characters in Line 408, addition of a legend to Figure 2a, and correction of notation such as E_Si .

9. **Improved reproducibility resources.**

We added a minimal reproducible example based on a toy dataset, together with a clearer tutorial in the GitHub repository.

Below please find the point-by-point response to all the reviewer’s comments. We marked the comments from reviewers in black and our responses in blue. For your convenience, we have not attached the relevant figures and tables to this response; they are available in the revised manuscript and supplementary materials under their respective names.

==

Response to Reviewer #1:

Comments for the Authors

The paper “Unify: Learning Cellular Evolution with Universal Multimodal Embeddings” presents a transfer learning framework designed to integrate single-cell RNA-sequencing data across species. The method introduces a novel multi-modal "macrogene" representation by combining protein embeddings (ESM-2) with functional text embeddings (LLaMA-2). Unify employs an adversarial autoencoder guided by a Pareto-optimal multi-task learning strategy (MGDA-UB) to balance reconstruction, batch correction, and biological conservation. The authors benchmark Unify against state-of-the-art methods and demonstrate its utility in identifying differentially expressed macrogenes, predicting cross-species perturbation responses, and reconstructing a 700-million-year cell-type evolutionary tree.

While the integration of multimodal embeddings and Pareto optimization is conceptually interesting, the manuscript lacks critical rigorous validation of these components. There are substantial concerns regarding the fairness of benchmarks, the necessity of complex architectural choices, and the robustness of the evaluation datasets.

We sincerely thank the reviewer for the thoughtful summary of our work and for the constructive and insightful comments. We appreciate the reviewer’s recognition of the conceptual contribution of Unify, particularly its integration of multimodal embeddings and Pareto-optimal multi-task learning for cross-species single-cell analysis. We also value the reviewer’s concerns regarding the rigor of validation, benchmark fairness, architectural complexity, and dataset robustness. In response, we have conducted additional analyses and experiments and revised the manuscript extensively to address these points. Below, we provide a detailed point-by-point response to each comment.

Major Concerns:

1. In Figure 4B–F, Unify-basal often performs on par with or even slightly better than Unify-macrogene, especially for the “All genes” setting. Since the text emphasizes macrogenes as a core innovation, it would be helpful if the authors could clarify in more detail what additional benefits Unify-macrogene provides beyond Unify-basal in this prediction task (e.g., interpretability, evolutionary analyses), and whether the performance differences between the two variants are statistically significant.

We sincerely thank the reviewer for this insightful comment. We agree that it is crucial to clearly distinguish the predictive nuances and the broader conceptual advantages of the macrogene framework compared to the basal embedding. To address this, we have performed the requested statistical analyses, clarified the interpretation of the evaluation metrics, and explicitly detailed the unique benefits of Unify-macrogene in the revised manuscript.

(1) Statistical Evaluation of Performance Differences

Per the reviewer's suggestion, we have added formal statistical comparisons between Unify-macrogene, Unify-basal, and all competing methods. We used paired two-sided t-tests across cell types/conditions, followed by Benjamini-Hochberg (BH) correction for multiple testing. The updated statistical significance levels are now incorporated into Figure 4b–h.

This statistical evaluation revealed that the relative performance of the two variants depends on the complexity of the task and the metric evaluated:

(a) **Highly Complex Task (IFN-β Perturbation)**: This task requires cross-context extrapolation (from in vivo mouse lymph node dataset to predict in vitro human PBMCs dataset). Here, Unify-macrogene achieves a significantly higher R^2 than Unify-basal (in both one-to-one and all-gene settings). Conversely, Unify-basal shows a modestly but significantly higher DEG hit rate (Figure 4b–d).

(b) **Less Complex Task (LPS Perturbation)**: This task involves matched in vitro data from the same cell type across species. Here, there are no statistically significant differences between the two variants across any metrics (R^2, DEG hit rate, or direction accuracy) (Figure 4f–h).

We interpret the divergent performance in the more challenging IFN-β task as reflecting the different nature of the two embedding spaces:

(a) R^2 (Global Transcriptome Recovery): R^2 measures the holistic, continuous agreement across the entire transcriptome. The macrogene framework excels here because it integrates structural and functional information across related genes, capturing broader transcriptional programs. This leads to a much better global agreement with the ground truth state.

(b) DEG Hit Rate (Local, Threshold-Dependent Signals): DEG hit rate relies on strict thresholds (e.g., p-adjusted-value). Because macrogenes distribute signals across

functionally correlated modules (resulting in smoother, more stable predictions), fewer individual genes may cross extreme fold-change thresholds. Unify-basal, lacking this module-level smoothing, preserves sharper, noisier individual gene-level contrasts, which can marginally improve the threshold-dependent DEG hit rate despite having weaker overall (R^2) transcriptome alignment.

(2) The Conceptual Advantage of the Macrogene Framework

Most importantly, as the reviewer rightly points out, the value of the macrogene framework extends beyond perturbation prediction accuracy. While Unify-basal provides strong integration performance, Unify-macrogene provides an interpretable biological coordinate system.

This macrogene framework enables gene-program-level interpretation through differential macrogene expression analysis, explicit analysis of evolutionary convergence and divergence by decoupling structural and functional similarity, and biologically grounded grouping of genes beyond strict one-to-one orthology. These capabilities underlie the analyses presented in Figure 3 and represent key biological advantages of Unify-macrogene that cannot be achieved using the basal embedding alone. In the context of perturbation prediction, the macrogene framework also improves interpretability by enabling comparison of responses at the level of coordinated gene programs rather than isolated genes, thereby helping to distinguish conserved from species-specific response mechanisms. Such program-level insights may be particularly valuable for translational studies, as they can highlight perturbation-associated programs that are more likely to generalize from model organisms to humans.

Changes to the manuscript:

To make this explicitly clear to the readers, we have expanded the main text in the Results section (Page 13, Line 388–394):

"*Direct comparison of the two Unify variants revealed distinct strengths. In the more challenging IFN-β task, Unify-macrogene achieved significantly higher R^2 than Unify-basal in both the one-to-one and all-gene settings, whereas Unify-basal showed a modest advantage in DEG hit rate. This pattern suggests that the macrogene framework tends to better capture broad transcriptional programs, whereas the basal variant is more sensitive to gene-specific changes that cross differential-expression thresholds. In the less complex LPS tasks, where perturbation signals were stronger and more conserved, no significant differences were observed between the two variants.*"

We added discussion on the benefit of Unify-macrogene could bring comparing to Unify-basal in the prediction tasks (Page 17, Line 486-494):

"*A similar principle of balance applies to Unify's dual representation spaces. The distinction between Unify-basal and Unify-macrogene in the perturbation prediction task highlights the complementary value of gene-level sensitivity versus biologically structured abstraction. While the basal variant provides strong predictive performance by maintaining fine-grained gene resolution, the macrogene framework introduces a higher-level, evolutionarily informed representation that better captures broad transcriptional programs. This structured representation enhances interpretability and provides a natural framework for gene-program-level comparison across species that are not accessible in the basal embedding alone. Together, the two variants illustrate how predictive accuracy and biological interpretability can be integrated within a unified cross-species framework.*"

We have also updated the Methods section to reflect the new statistical tests (Page 24, Line 742-746):

"*Statistical comparisons of method performance in perturbation prediction were performed in R (stats package) using paired two-sided t-tests across cell types/conditions, comparing Unify-macrogene and Unify-basal against each other and all competing methods. Resulting p-values were adjusted for multiple testing using the Benjamini-Hochberg procedure.*"

2. While the choice of ESM and LLaMA is reasonable and well-motivated, the manuscript would benefit from a brief discussion of why these particular foundational models were selected, and whether alternative protein or text models (e.g., ProtT5, ProtTrans, PubMedBERT, Bloom, or other LLMs) might yield similar or improved performance. Since multimodal macrogene construction is a central innovation of Unify, even a small benchmark or an ablation comparing different foundational models—or a discussion explaining why such benchmarking is unnecessary—would help strengthen the rationale and reproducibility of this component.

We thank the reviewer for this highly constructive suggestion. We entirely agree that justifying the choice of foundational models strengthens the rationale for our multimodal macrogene

construction. In response, we have clarified our selection rationale and conducted a comprehensive new ablation benchmark comparing alternative protein and text models.

(1) Rationale for Model Selection

Our initial selection of ESM and LLaMA was driven by their standing as state-of-the-art, highly accessible models in their respective domains:

ESM1/2: ESM is widely recognized as a gold-standard protein language model. Its embeddings capture evolutionarily meaningful protein features at scale. Furthermore, prior leading work (e.g., SATURN[1], UCE[2]) has validated the specific effectiveness of ESM embeddings for learning universal cross-species gene representations, making it the most rigorously tested baseline for the structural modality.

LLaMA: We selected LLaMA because it is a state-of-the-art, fully open-weights large language model with exceptional semantic reasoning capabilities. Because gene functions are often described in diverse, complex natural language formats across different databases, a highly capable general-purpose LLM ensures robust encoding of functional context.

However, as we noted in the Methods section, the Unify framework is fundamentally modular; any high-quality sequence or text model can theoretically be plugged into the pipeline.

(2) New Ablation Benchmark (Alternative Foundation Models)

To empirically validate our choice, we took the reviewer's excellent suggestion and performed a new ablation benchmark testing alternative foundation models. We evaluated a total of nine combinations of alternative protein models (e.g., ESM[3], ProstT5[4], ProtT5[5]) and alternative text models (e.g., LLaMA[6], PubMedBERT[7], BLOOM[8]).

We tested these various protein and text combinations across 5 different cross-species integration tasks spanning various phylogenetic distances.

Results: The results of this benchmark are now provided in Supplementary Figure 22 and Supplementary Table 7. While several alternative combinations yielded competitive integration performance (confirming the generalizability of Unify's multimodal architecture), the ESM + LLaMA pairing achieved the highest average performance, particularly in biological conservation and the overall integration score. This empirically justifies our default model pairing.

Changes to the manuscript:

To ensure readers understand the rationale and robustness of these design choices, we have added a dedicated summary of our ablation studies to the main text. We have also added a new Supplementary Figure 22 and Supplementary Table 7 detailing the ablation results.

"*In this study, ESM was used to generate protein-based embeddings because Its suitability is supported by previous work, which showed that ESM-based embeddings are effective for constructing biologically meaningful macrogene representations in cross-species integration.*" (Page 19, Line 572-574)

"*LLaMA-2 was chosen because it provides a strong, fully open-weight framework for representing semantic information from functional descriptions. As gene functions are often described in diverse and complex natural-language formats across databases, a general-purpose language model with robust semantic understanding offers an effective way to encode functional context.*" (Page 20, Line 587-591)

"*While performance remained broadly robust across different foundation-model combinations, distinct trade-offs between protein and text encoders were observed. Multiple pairings of protein language models (ESM, ProstT5, and ProtT5) and text models (LLaMA, BLOOM, and PubMedBERT) performed competitively, demonstrating that Unify is not dependent on a specific embedding source (Supplementary Figure 22, Supplementary Table 7). However, the ESM + LLaMA combination emerged as the most effective, achieving the highest bio-conservation while retaining competitive batch-correction performance.*" (Page 7, Line 228-234)

We also included the relevant description in the Methods section:

"*To assess dependence on foundation-model choice, alternative combinations of protein language models (ESM, ProstT5, and ProtT5) and text models (LLaMA, BLOOM, and PubMedBERT) were benchmarked. ... In all ablation analyses, model parameters and training settings were kept identical to those of the Unify model, and only the component under investigation was modified. Performance was assessed using the same integration metrics as in the main benchmark."* (Page 26, Line 790-804)

3. Absence of ablation studies: There is a complete absence of ablation studies to justify the model's complexity. It is unclear whether the reported performance gains stem from the novel multimodal embeddings, the specific optimization algorithm, or simply the supervised nature of the training. The authors must deconstruct their model to validate the necessity of each component:

a) Pareto Optimization: The authors claim MGDA-UB is superior to fixed weighting. An ablation comparison of Unify (MGDA-UB) vs. Unify (Fixed Weights/Grid Search) is required to prove the robustness of this contribution.

Thank you for this important comment. We agree that it is essential to disentangle the contributions of individual components of the Unify model and to justify architectural choices.

In response, we performed a dedicated ablation study comparing Unify with MGDA-UB Pareto optimization to Unify with fixed loss weighting across multiple cross-species integration tasks. For the fixed-weight baseline, we evaluated multiple configurations of the reconstruction loss (α), species classifier loss (β), and cell-type classifier loss (γ), including four representative weight combinations that span commonly used regimes (reconstruction-dominant, batch-correction-dominant, balanced, and biologically-conservation-dominant weighted settings).

Across all evaluated species-integration tasks, Unify (MGDA-UB) consistently achieved higher average batch-correction scores and biological conservation scores than any fixed-weight configuration. Importantly, the fixed-weight variants showed substantial sensitivity to hyperparameter choices, with performance varying markedly across tasks and weight settings, whereas MGDA-UB provided stable, robust optimization without manual tuning.

These results, now reported in Supplementary Table 6 and Supplementary Figure 21, demonstrate that adaptive multi-objective balancing via MGDA-UB can improve the model’s performance compared to the fixed weight methods. This ablation, therefore, supports the contribution of Pareto optimization as a core component of the Unify framework.

Changes to the manuscript:

“The choice of optimization strategy proved equally critical to model performance. Training with MGDA-UB yielded significantly higher bio-conservation and overall scores compared to fixed loss weighting (Supplementary Figure 21, Supplementary Table 6). These findings suggest that the inherent trade-off between species mixing and preservation of cell identity is better resolved

through Pareto-optimal multi-objective optimization than through manually specified, static weights." (Page 7, Line 223-227)

We also included the relevant description in the Methods section:

"*For the optimization ablation, Unify (MGDA-UB) was compared with fixed-weight variants in which the relative contributions of the reconstruction loss (α), species-classifier loss (β), and cell-type-classifier loss (γ) were manually specified. ... In all ablation analyses, model parameters and training settings were kept identical to those of the Unify model, and only the component under investigation was modified. Performance was assessed using the same integration metrics as in the main benchmark."* (Page 26, Line 785-804)

b) Cell-type Supervision: The model uses a cell-type classifier loss. The authors should test the model without this supervision signal to determine its impact on bio-conservation scores.

We thank the reviewer for raising this important point regarding the role of cell-type supervision in Unify. In response, we conducted a targeted ablation study to explicitly assess the impact of the cell-type classifier loss on integration performance.

To thoroughly address this, we evaluated three training variants across multiple cross-species integration tasks:

- Unify (fully supervised): Using cell-type labels for all species.
- Unify (semi-supervised): Where one species retains true cell-type labels, while the other species uses unsupervised cluster IDs (simulating a scenario where only a reference atlas is annotated).
- Unify (no cell-type supervision): In which the cell-type classifier loss is completely removed.

(1) The Theoretical Necessity of Supervision in Adversarial Integration

The results of this ablation study (Supplementary Figure 23, Table 8) are consistent with the theoretical design of adversarial integration models. When the cell-type classifier loss is completely removed, the model is left with only two objectives: reconstruction and an adversarial penalty to mix species. In this scenario, the optimal path for the adversarial network is to simply

collapse the latent space, mushing disparate cell populations together into a biologically meaningless distribution to aggressively fool the species discriminator.

Consequently, as expected, removing the cell-type classifier entirely leads to a marked, substantial reduction in biological conservation scores. This suggests that the supervision signal is not merely an enhancement; it acts as the essential biological counter-weight to the adversarial species-mixing loss, ensuring that homologous cell states are aligned correctly rather than indiscriminately.

(2) The Power of Partial Supervision

Crucially, our new experiments also demonstrate that Unify does not require perfect, global annotation to succeed.

While the fully supervised Unify model consistently achieves the highest biological conservation scores, the semi-supervised variant remains highly competitive. Despite relying on unsupervised cluster IDs for one species, this variant still outperforms most baseline methods in bio-conservation. This indicates that Unify can effectively leverage partial supervision, using the structure of one well-annotated reference atlas to guide the integration of a newly generated, unannotated dataset from another species.

Changes to the manuscript:

We have added a new Supplementary Figure 23 and Supplementary Table 8 detailing these ablation results. We have also updated the main text to explicitly discuss the role of this supervision:

"We assessed the contribution of biological supervision by testing a semi-supervised variant, where unsupervised cluster identities replaced one species' cell-type labels, and a fully unsupervised variant lacking the cell-type classifier entirely (Supplementary Figure 23, Supplementary Table 8). Remarkably, Unify (semi-supervised) retained strong bio-conservation performance, performing comparably to SAMap and becoming increasingly competitive as phylogenetic distance increased. These results indicate that while explicit cross-species supervision maximizes alignment, it is not the sole source of biological structure. The multimodal macrogene priors alone capture substantial conserved biological signals, allowing Unify to remain highly competitive even when labels are missing or unaligned." (Page 7, Line 235-243)

We also included the relevant description in the Methods section:

"*For the supervision ablation, three variants were evaluated: Unify, the original model uses cell type labels from both input species; Unify (semi-supervised), in which one species retained curated cell-type labels while the other used unsupervised cluster identities; and Unify (no cell-type classifier), in which the cell-type classifier was removed entirely. ... In all ablation analyses, model parameters and training settings were kept identical to those of the Unify model, and only the component under investigation was modified."* (Page 26, Line 793-804)

c) Multimodality: The authors must demonstrate that the inclusion of text-based embeddings provides a statistically significant performance gain over using protein embeddings alone. A comparison of Unify (Full) vs. Unify (Only ESM-2) vs. Unify (Only LLaMA-2) is essential. Without this, it is difficult to conclude that the LLM contributes meaningfully to performance, and the inclusion may appear unnecessary.

Thank you for raising this important point about the need to incorporate text-based embeddings. In response, we performed a dedicated ablation study to explicitly quantify the contribution of multimodality in Unify.

We compared three model variants across multiple cross-species integration tasks spanning different evolutionary distances:

- Unify (Full), incorporating both protein-based (ESM) and text-based (LLaMA) embeddings.
- Unify (ESM-only), using only protein embeddings.
- Unify (LLaMA-only), using only text embeddings.

Across all evaluated tasks, Unify (Full) achieved higher biological conservation and overall integration scores than either unimodal variant. These results are now reported in Supplementary Table 5 and Supplementary Figure 20. Notably, the unimodal variants exhibit complementary strengths: protein embeddings alone better preserve structural similarity, while text embeddings alone capture higher-level functional semantics but lack sequence-level resolution. Their combination in Unify enables a more comprehensive representation that jointly captures structural and functional signals, thereby improving cross-species alignment and biological conservation.

Together, these results demonstrate that the inclusion of text-based embeddings is not redundant but instead provides a significant performance gain when integrated with protein embeddings, supporting the necessity of the multimodal macrogene design.

We have updated the Results section to contain this new ablation study:

"*Across ten integration tasks spanning cross-genus to cross-phylum comparisons, the full multimodal model, initialized with both ESM-derived protein embeddings and LLaMA-derived functional text embeddings, outperformed the single-modality variants. The full model achieved significantly higher bio-conservation and overall scores (Supplementary Figure 20, Supplementary Table 5). This indicates that structural and functional priors provide complementary information for macrogene construction, enabling the latent space to better preserve shared cellular programs across vast evolutionary distances.*" (Page 7, Line 216-222)

We also included the relevant description in the Methods section:

"*For the multimodality ablation, three model variants were compared: Unify, using both ESM-derived protein embeddings and LLaMA-derived functional text embeddings; Unify (ESM-only), using only protein embeddings; and Unify (LLaMA-only), using only text embeddings. ... In all ablation analyses, model parameters and training settings were kept identical to those of the Unify model, and only the component under investigation was modified. Performance was assessed using the same integration metrics as in the main benchmark.*" (Page 25, Line 782-804)

4. Insufficient benchmark datasets: The benchmarking section relies on a very limited set of species pairs—only one pair per phylogenetic level (e.g., one cross-genus pair, one cross-family pair). This is insufficient to establish the stability and generalizability of the method. The authors should include additional cross-genus and cross-family pairs to demonstrate that Unify's performance is consistent and statistically significant compared to other tools across varied biological contexts.

Thank you for this important comment. We agree that broader benchmarking across multiple species pairs is necessary to better demonstrate the stability and generalizability of Unify across phylogenetic contexts.

In response, we expanded the benchmark dataset collection by adding one additional dataset for each cross-species integration category, resulting in a broader evaluation across 10 cross-species integration tasks as below Table 1 shows (details provided in the updated Supplementary Table 2). Accordingly, we also updated Figure 2 to reflect the expanded benchmarking design.

To further assess robustness, we performed pairwise statistical comparisons between Unify and baseline methods across all 10 tasks using paired two-sided t-tests with multiple-testing corrections. The results show that Unify consistently achieves significantly higher overall scores than all baseline methods. In particular, Unify shows significantly higher biological conservation scores than all methods except SATURN, while also achieving significantly stronger batch correction performance than SATURN.

The results for the additional datasets are now provided in Supplementary Figures 1-18 and Supplementary Table 3, further supporting that Unify maintains strong and stable performance across diverse biological settings and evolutionary distances.

Table 1. Datasets used in each cross-species integration category

Task	Species	Cell amount	Mean_UMI/cell
Cross genus	*Felis catus* (Cat)	11037	4908.85
	Panthera tigris altaica (Tiger)	16163	5063.33
	Felis catus (Cat)	49625	1800.66
	Panthera tigris altaica (Tiger)	5844	5251.59
Cross family	*Felis catus* (Cat)	11037	4908.85
	Canis lupus familiaris (Dog)	7453	4283.9
	Homo sapiens (Human)	15086	7217.65
	Macaque mulatta (Monkey)	3073	7801.41
Cross order	*Nematostella vectensis* (Sea anemone)	10581	1025.46
	Stylophora pistillata (hard coral)	16080	1473.34
	Macaque mulatta (Monkey)	1889	4205.98
	Sus scrofa (Pig)	2541	7124.78
Cross class	*Xenopus tropicalis* (Frog)	96935	3225.71
	Danio rerio (Zebrafish)	63371	6098.8
	Danio rerio (Zebrafish)	3816	1508.38
	Homo sapiens (Human)	1770	4614.18
Cross phylum	*Drosophila melanogaster* (Fly)	156524	1066.37
	Danio rerio (Zebrafish)	72732	2021.74
	Strongylocentrotus purpuratus (Sea urchin)	60399	4812.33
	Danio rerio (Zebrafish)	66381	2021.74

Changes to the manuscript:

"*To assess the statistical robustness of the benchmark results, we performed pairwise comparisons across integration tasks. Unify achieved significantly higher overall scores than baseline methods (Supplementary Figures 17, 18a).*" (Page 6, Line 189-191)

5. Unfair comparison regarding supervision: Unify explicitly utilizes cell type annotation information during training, whereas many competing methods are unsupervised. For a fair evaluation, the benchmark results should clearly distinguish between methods that require cell type labels (e.g., Unify, SATURN) and those that do not.

We thank the reviewer for highlighting this important point. We completely agree that clearly distinguishing between supervised (label-informed) and fully unsupervised methods is essential for a fair and transparent interpretation of the benchmarking results.

As the reviewer correctly notes, Unify is designed primarily as a tool for the deep comparative analysis of *already-annotated* cell atlases, rather than for *de novo* cell-type discovery in a completely unlabeled setting. To ensure this context is clear to all readers, we have updated the manuscript in the following three ways:

(1) Explicit Categorization in Benchmarks

We have added a new summary table (Supplementary Table 1) that explicitly categorizes the supervision requirements of every method evaluated in our study. Furthermore, we have revised the main text and figure captions to clearly distinguish label-informed methods (Unify, SATURN, and scGen), which utilize cell-type labels during at least part of model fitting, from unsupervised methods (e.g., fastMNN, SAMap, scVI, Seurat), which do not.

(2) Clarifying the Evaluation Context

We have revised the benchmark description to emphasize that Unify is evaluated in a label-aware integration setting. We explicitly state that its performance should be interpreted in this context—leveraging established biological knowledge to create a high-fidelity shared space for cross-species comparison—rather than as a direct drop-in replacement for unsupervised clustering pipelines.

(3) Performance Under Reduced Supervision

Crucially, while Unify benefits from full supervision, its performance gains are not solely driven by access to labels. As demonstrated in our new ablation analyses (prompted by Reviewer's Comment 3), Unify's multimodal macrogene representation and Pareto optimization strategy provide significant advantages even when labels are scarce. When operating in a semi-supervised mode (where only one species is labeled and the other relies on unsupervised clustering), Unify remains highly competitive with, and often outperforms, fully unsupervised baselines (Supplementary Table 8, Supplementary Figure 23).

Changes to the manuscript:

These clarifications have been incorporated into the revised manuscript.

"*To ensure a fair and transparent comparison, we explicitly distinguish between "label-informed" methods, which utilize cell-type annotations to guide model fitting (e.g., Unify, SATURN, scGen), and fully "unsupervised" methods (e.g., SAMap, scVI) (Supplementary Table 1).*" (Page 6, Line 165-167)

"*We assessed the contribution of biological supervision by testing a semi-supervised variant, where unsupervised cluster identities replaced one species' cell-type labels, and a fully unsupervised variant lacking the cell-type classifier entirely (Supplementary Figure 23, Supplementary Table 8). Remarkably, Unify (semi-supervised) retained strong bio-conservation performance, performing comparably to SAMap and becoming increasingly competitive as phylogenetic distance increased. These results indicate that while explicit cross-species supervision maximizes alignment, it is not the sole source of biological structure. The multimodal macrogene priors alone capture substantial conserved biological signals, allowing Unify to remain highly competitive even when labels are missing or unaligned."* (Page 7, Line 235-243)

We believe these revisions make the comparisons fully transparent and allow readers to interpret the performance of all methods in light of the supervision available to them.

6. Dependency on cell type labels: It is unclear how Unify handles scenarios where cell type labels are missing or inconsistent. The authors should evaluate: 1) Performance when one or both datasets lack cell type annotations. 2) Performance when labels are not aligned or comparable. This is critical for real-world applications where ground-truth cross-species annotations are often unavailable.

We thank the reviewer for raising this critical point regarding real-world applications. We completely agree that in many cross-species scenarios, annotations may be missing, incomplete, or lack a 1-to-1 consensus mapping.

To directly evaluate Unify's robustness under the exact scenarios the reviewer described, we conducted a systematic ablation analysis evaluating three distinct training regimes:

(1) Evaluating Performance with Missing Annotations (Reviewer's Point 1)
To simulate environments where one or both datasets lack annotations, we tested:

- Unify (Unsupervised - Both Missing): The cell-type classifier loss is entirely removed. As discussed previously, while this removes batch effects, the lack of a biological counterweight to the adversarial loss leads to a clear reduction in biological conservation.
- Unify (Semi-supervised - One Missing): One species utilizes curated cell-type annotations, while the other species is provided only with unsupervised cluster IDs (e.g., from Leiden clustering).

Results: Strikingly, the semi-supervised variant remained highly competitive, outperforming most baseline methods in bio-conservation despite missing ground-truth labels for an entire species. This demonstrates that Unify can effectively leverage a single annotated "reference" atlas to integrate an unannotated query dataset.

(2) Evaluating Performance with Unaligned/Non-Comparable Labels (Reviewer's Point 2)
The Semi-supervised regime described above simultaneously tests the scenario where labels are unaligned or non-comparable. Because the second species uses arbitrary cluster IDs (e.g., "Cluster 0", "Cluster 1"), while the first uses semantic labels (e.g., "Macrophage", "T-cell"), there is no explicit or pre-existing mapping between the two datasets' label sets.

Results: The strong performance of Unify that is comparable with SAMap in this setting indicates that the framework *does not* strictly require perfectly matched, harmonized annotations across

species. The network successfully learns to align unaligned cluster IDs with semantic labels in the shared latent space, based on underlying transcriptional and macrogene similarities.

Changes to the manuscript:

While Unify (fully supervised) achieves the highest biological conservation (representing an upper bound when perfect labels are available), these new experiments confirm that Unify remains highly applicable and competitive in realistic, messy scenarios where annotations are sparse or inconsistent.

We have added these analyses (Supplementary Figure 23, Supplementary Table 8) and updated the manuscript to explicitly discuss Unify's performance in semi-supervised and unaligned scenarios:

"We assessed the contribution of biological supervision by testing a semi-supervised variant, where unsupervised cluster identities replaced one species' cell-type labels, and a fully unsupervised variant lacking the cell-type classifier entirely (Supplementary Figure 23, Supplementary Table 8). Remarkably, Unify (semi-supervised) retained strong bio-conservation performance, performing comparably to SAMap and becoming increasingly competitive as phylogenetic distance increased. These results indicate that while explicit cross-species supervision maximizes alignment, it is not the sole source of biological structure. The multimodal macrogene priors alone capture substantial conserved biological signals, allowing Unify to remain highly competitive even when labels are missing or unaligned." (Page 7, lines 235-243)

We also included the relevant description in the Methods section:

"*For the supervision ablation, three variants were evaluated: Unify, the original model use cell type labels from both input species; Unify (semi-supervised), in which one species retained curated cell-type labels while the other used unsupervised cluster identities; and Unify (no cell-type classifier), in which the cell-type classifier was removed entirely. ... In all ablation analyses, model parameters and training settings were kept identical to those of the Unify model, and only the component under investigation was modified. Performance was assessed using the same integration metrics as in the main benchmark."* (Page 26, Line 793-804)

7. Discrepancy in Trajectory Conservation: In Supplementary Table 2, the "Trajectory conservation" scores for Unify in Cross-genus and Cross-family tasks appear significantly lower or inconsistent compared to other metrics or tools. The authors should explain this phenomenon in the Results or Discussion section. Does the method trade off trajectory preservation for cluster distinctness?

We thank the reviewer for this keen observation. The reviewer's hypothesis, that the method trades off trajectory preservation for cluster distinctness, is indeed correct and gets to the heart of Unify's optimization strategy.

The comparatively lower trajectory conservation scores, particularly in closely related species where continuous developmental manifolds are naturally prominent, are a direct consequence of Unify's cell-type supervision**.** Because Unify utilizes a classification loss to ensure rigorous alignment of homologous cell states across species, the latent space is optimized for discrete separability (cluster distinctness). Consequently, the model tends to pull cells into tight, distinct, label-specific clusters. While this is highly effective for maximizing cross-species alignment, batch correction, and cluster-level bio-conservation, it inevitably "shatters" the continuous transitional states that make up developmental trajectories.

By contrast, fully unsupervised methods (which do not force cells into discrete classes) naturally preserve these continuous pseudo-temporal bridges, yielding higher trajectory scores but often failing to accurately align distinct homologous clusters across vast evolutionary distances. Unify therefore tends to favor accurate mapping of distinct cell identities across species over the preservation of continuous intra-species developmental topologies.

Changes to the Manuscript:

We have explicitly clarified this architectural trade-off in the revised manuscript:

"Interestingly, Unify exhibited comparatively lower trajectory conservation scores in integrations of closely related species, highlighting a trade-off inherent to supervised cross-species alignment. Although cell-type labels are not enforced as strict matching constraints, the classification objective still promotes compact, label-consistent organization in the latent space. This favors accurate cross-species alignment and strong cluster-level biological conservation, but may compress transitional intermediate states and thereby reduce trajectory conservation. In these settings, Unify therefore favors robust alignment of annotated cell identities over strict preservation of fine-scale developmental continuity. To assess the statistical robustness of the

benchmark results, we performed pairwise comparisons across integration tasks. Unify achieved significantly higher overall scores than baseline methods (Supplementary Figures 17, 18a). This result remained robust across alternative weighting schemes, with Unify retaining the highest average overall score and remaining significantly better than most competing methods (Supplementary Figure 18b-f)." (Page 6, Line 182-193)

8. Computational resource reporting: Given the use of large foundation models (ESM-2, LLaMA-2) and adversarial training, the computational cost is a concern. The authors should provide detailed statistics on the hardware used (GPU/CPU type), memory/VRAM usage, and runtime for the major experiments.

Thank you for this insightful suggestion. We agree that reporting computational requirements is important for evaluating the practical applicability of a new integration framework, particularly given the use of foundation-model-derived embeddings and adversarial multi-objective training in Unify. We would like to clarify that, since we use the pretrained ESM/LLaMa embeddings, the cost of feature extraction is fixed; however, the overall computational cost during training and inference still scales with dataset size.

In response, we performed an additional computational benchmark using a representative large-scale integration task between *Danio rerio* and *Strongylocentrotus purpuratus*, which poses a challenging scenario due to a substantial imbalance in dataset sizes (approximately 1 million cells versus 60,000 cells). To systematically assess scalability, we subsampled the zebrafish dataset at 1%, 6%, 20%, 40%, 60%, 80%, and 100%, generating datasets ranging from approximately 70,000 to 1.1 million cells.

For each method, we recorded hardware configuration, memory usage, and runtime across all dataset sizes. These results are now summarized in Supplementary Table 4 and Supplementary Figure 19, including CPU/GPU configuration, memory consumption, and runtime statistics.

The analysis shows that lightweight methods such as Harmony, scVI, and Scanorama remain highly efficient and complete integration within one hour even at million-cell scale. In contrast, methods such as Seurat and graph-based approaches, including SAMap show substantial runtime increases as dataset size grows.

Unify demonstrated favorable scalability across all tested dataset sizes. Runtime increased smoothly from medium-scale datasets to approximately 1–5 hours at 1.1 million cells, remaining consistently below SATURN (which is the deep learning-based method most similar to ours) and

substantially below Seurat v4 CCA at large scale. Although slower than lightweight methods such as Harmony and fastMNN, Unify maintained a practical computational profile while delivering stronger biological integration performance.

We have further revised the manuscript to contain this assessment:

"*Beyond its strong integration performance, Unify also showed favorable computational scalability across datasets ranging from 70K to 1.1M cells. It scaled more favorably than several competing cross-species integration approaches, including SATURN, SAMap, Seurat v4 CCA, and BBKNN, particularly at larger dataset sizes (Supplementary Figure 19, Supplementary Table 4). This indicates that Unify combines strong integration accuracy with practical scalability for large comparative atlases.*" (Page 7, Line 204-209)

Minor:

1. Line 122 refers to 'Figure 1A', but only 'Figure 1' appears in the manuscript.

Thank you for pointing out this inconsistency. We apologize for the confusion caused by the incorrect figure reference. We have corrected the text to refer to Figure 1 instead of Figure 1A to ensure consistency between the manuscript text and the figure labeling.

2. In line 133, MGDA-UB appears for the first time in the Results section, but its full name is only provided later in the Methods (line 544). Readers who are not familiar with this algorithm may find it confusing. I suggest adding the full term when MGDA-UB is first introduced.

Thank you for this helpful suggestion. We agree that introducing abbreviations without definition can be confusing. We have now added the full name "Multi-Gradient Descent Algorithm – Upper Bound algorithm" for MGDA-UB at its first occurrence in the Results section (line 133), ensuring that readers are provided with the necessary context before encountering the abbreviation.

3. Authors may cite previous benchmark studies to justify the use of a 40% batch-correction and 60% bio-conservation weighting scheme, but the manuscript does not explain why this ratio is appropriate for the present study. Given that the overall score is central to method ranking, it

would be helpful for the authors to provide a brief rationale or sensitivity analysis showing that the conclusions are robust to alternative weighting choices.

Thank you for raising this important point regarding the weighting scheme used to compute the overall integration score. We agree that, because the overall score directly affects method ranking, its formulation should be explicitly justified.

The 40% batch-correction and 60% biological conservation weighting follow established practice in previous large-scale benchmarking studies, including scIB[9], where biological conservation is intentionally given greater weight to reduce the risk of over-correction that may artificially remove biologically meaningful variation. In the context of cross-species integration, this consideration is particularly important because preserving species-shared biological structure is a primary objective of the analysis.

To assess whether our conclusions depend on this weighting choice, we additionally performed a sensitivity analysis using alternative weighting schemes (including **50:50**, **30:70, 40:60, 60:40, 70:30** weights between batch-correction to bio-conservation). Across these settings, the relative ranking of methods remained highly consistent, and the overall conclusions regarding Unify's performance were unchanged (Supplementary Figure 18).

These results indicate that Unify's performance advantage is not driven by a specific weighting definition, but reflects robust improvements across both batch correction and biological conservation metrics. We have added this clarification and the corresponding sensitivity analysis to the revised manuscript.

Changes to the manuscript:

"*This weighting acknowledges that while removing technical artifacts is essential, preserving shared biological structure remains the paramount objective of cross-species comparative analysis.*" (Page 6, Line 172-174)

"*To assess the statistical robustness of the benchmark results, we performed pairwise comparisons across integration tasks. Unify achieved significantly higher overall scores than baseline methods (Supplementary Figures 17, 18a). This result remained robust across alternative weighting schemes, with Unify retaining the highest average overall score and remaining significantly better than most competing methods (Supplementary Figure 18b-f).*" (Page 6, Line 189-193)

4. Line 228 contains a typographical error: “marcogenes” should be corrected to “macrogenes.”

Thank you for pointing out this typographical error. We have corrected “marcogenes” to “macrogenes” in Line 228 of the revised manuscript.

5. In Figure 3C, the red stars marking known marker genes are not clearly visible for some species. Improving their visibility would enhance the readability of the figure.

Thank you for this helpful suggestion. We have improved the visibility of the red star markers in Figure 3c by increasing their size and contrast and adjusting their layering to ensure they are clearly visible across all species. These changes enhance the readability of the figure without altering the underlying results.

6. Authors refer to “top DEGs” when evaluating perturbation prediction performance (e.g., Figure 4D), but the manuscript does not define how “top DEGs” are selected (e.g., based on log fold change, adjusted p-value, fixed number of genes, or percentage thresholds). Since these definitions directly influence hit rate and direction accuracy, a clear explanation in the Methods section would improve reproducibility.

We thank the reviewer for this suggestion. We have added the explanation in the Methods section:

“*Datasets were first filtered to retain cells with at least 200 detected genes and genes expressed in at least 10 cells. The filtered data were then normalized and log1p-transformed. Differentially expressed genes (DEGs) were identified using the rank_genes_groups function in SCANPY with multiple-testing correction applied to obtain adjusted p-values. Genes with adjusted p-value < 0.05 were defined as significant DEGs. For top-N DEG evaluation, significant genes were ranked by descending absolute log fold change, and the highest-ranked genes were used for hit-rate and directional accuracy analyses.*” (Page 24, Line 728-734)

Response to Reviewer 2:

Comments for the Authors

Zhong et al. proposed a computational method, Unify, to align cells across species through macrogenes. The idea is an extension of a previous framework, SATURN, in which macrogenes are initiated through structural embeddings; Unify further incorporates functional gene embeddings to initiate macrogenes. Unify is claimed to perform better at cell alignment, characterizing evolutionary functional divergence, predicting perturbation effects across species, and inferring cell-type trees. Although these applications are important to the field, the description of the method is not clear, and the results are not convincing in the following areas.

We sincerely thank the reviewer for the careful evaluation of our manuscript and for the concise summary of our work. We appreciate the reviewer's recognition of the importance of the applications addressed by Unify, including cross-species cell alignment, characterization of evolutionary functional divergence, perturbation prediction, and cell-type tree inference. We also thank the reviewer for the constructive comments regarding the clarity of the method description and the strength of the supporting results. However, Unify is not a straightforward extension of SATURN, but a distinct framework with a different model architecture, representation strategy, and training paradigm. Whereas SATURN relies primarily on protein-based initialization, Unify introduces a universal multimodal macrogene framework that integrates both structural and functional embeddings, and optimizes integration through Pareto-optimal multi-task learning rather than the original SATURN training strategy. These differences are not merely incremental, but enable new capabilities beyond cross-species alignment alone. In addition to achieving stronger integration performance than SATURN in our benchmarks, Unify also supports analyses that SATURN does not, including cross-species perturbation prediction and systematic characterization of functional divergence and convergence. In response, we have substantially revised the manuscript to improve methodological clarity and strengthen the evidence supporting our conclusions. In particular, we have added a more systematic evaluation of functional divergence and convergence, as well as additional ablation studies examining the contributions of functional and structural embeddings. Below, we provide a detailed point-by-point response to each comment.

1. The notation in Line 408 is not shown in the pdf format.

Thank you for bringing this formatting issue to our attention. We have corrected the notation in Line 408 to ensure that it is properly rendered and visible in the PDF version of the manuscript. We apologize for the inconvenience caused by this display error.

2. Some notations are not intuitive – e.g., on Line 466, species is indicated as Si, but then Ei is introduced as average protein embedding. Should it be E_Si? What is it averaged across?

Thank you for pointing out this ambiguity. We agree that the original notation was not sufficiently clear. In this context, the protein embedding is averaged across all protein isoforms corresponding to the same gene, since a single gene may produce multiple protein sequences due to alternative splicing. We now explicitly clarify this in the revised manuscript and have updated the notation (e.g., using $e_{g_{si}}^{prot}$) to make the definition and averaging procedure clear and intuitive. We also reviewed and corrected other potentially ambiguous notations throughout the manuscript.

"*For each gene g_{si} from species S_i, we obtained its protein embedding $e_{g_{si}}^{prot} \in \mathbb{R}^{d_p}$, derived from pretrained protein language models. For genes encoding multiple protein isoforms, protein embeddings were generated for each isoform sequence independently and subsequently averaged to represent the corresponding coding gene. All embeddings across species are concatenated into a joint matrix:*

$$\mathcal{E}^{prot} = \left\{ e_{g_{si}}^{prot} \;\middle|\; \forall i, \forall s \right\} \in \mathbb{R}^{\mathrm{N} \times d_p}$$

"(Page 20, Line 605-610)

3. Regarding cell alignment performance comparison, it is not fair as Unify already has seen cell type labels, thus it is not surprising that it outperforms others at aligning cell types. The question is, if cell type labels and matching across species are already given, why do we need to align single cells across species?

Thank you for raising this important point regarding the fairness and interpretation of cell alignment performance. We agree that Unify leverages cell-type supervision during training. However, Unify is not the only supervised method in our benchmark; methods such as SATURN and scGen also use cell type labels during training. To make this distinction explicit, we have added a new supplementary table (Supplementary Table 1) summarizing the supervision regime of all benchmarked methods, and we now highlight this point in the revised main text (Page 6, Line 165-167).

Importantly, in Unify, cell-type supervision serves as a soft biological prior rather than a hard alignment constraint. It encourages biologically plausible correspondences in the embedding space but does not enforce exact overlap between similarly labeled populations or prescribe higher-order relationships across species. As a result, the learned embedding is not simply a recapitulation of the input annotations but supports comparative analyses beyond discrete label matching.

More importantly, the purpose of cross-species cell alignment in Unify extends well beyond reproducing existing cell-type labels. In practice, single-cell annotation in non-model species is typically inferred in two main ways: either from canonical marker genes defined in well-studied model species and transferred through one-to-one orthologs or broader homology relationships, or from expression-profile similarity, often after restricting comparisons to one-to-one ortholog space. Both strategies capture only part of the underlying cellular-state information and become increasingly limited as phylogenetic distance grows. Consequently, identically named cell types across species do not necessarily represent strictly homologous cell types or fully equivalent cellular states; instead, they may reflect only partially overlapping or functionally analogous programs. This is precisely why cross-species integration remains necessary: it enables us to move beyond discrete annotations and ask broader comparative questions, such as how cell types evolved and diversified, how perturbation responses can be transferred across species, and how conserved versus lineage-specific cellular functions are maintained or lost through evolution. To address these questions, cross-species integration is needed to reduce technical batch effects and establish a shared space for biologically meaningful comparison.

Within this context, Unify uses cell-type supervision only as a soft biological prior while learning continuous cell-level representations that support downstream cross-species perturbation prediction, cell type tree construction, evolutionary and functional analyses. We have clarified this motivation in the revised manuscript.

Changes to manuscript:

"*To ensure a fair and transparent comparison, we explicitly distinguish between "label-informed" methods, which utilize cell-type annotations to guide model fitting (e.g., Unify, SATURN, scGen), and fully "unsupervised" methods (e.g., SAMap, scVI) (Supplementary Table 1).*" (Page 6, Line 165-167)

"*Although Unify leverages cell-type annotations during training, these labels are used as a soft biological prior rather than as a rigid alignment constraint. This is particularly important in cross-species settings, where annotations, especially in non-model organisms, often represent approximate rather than strict one-to-one correspondences. In practice, such labels are frequently inferred from canonical marker genes or expression similarity within a limited ortholog space, and therefore may not define fully equivalent cellular states across species. Accordingly, the main value of Unify lies not in de novo cell-type discovery, but in enabling comparative analysis in a shared embedding space that goes beyond discrete label assignments. Rather than forcing identically labeled populations into exact overlap, Unify supports the analysis of comparative relationships among partially overlapping cell states, thereby providing a basis for downstream analyses such as cell-type tree construction, evolutionary inference, perturbation prediction, and the identification of conserved and species-specific regulatory programs.*" (Page 17, Line 475-485)

4. How were hyperparameters tuned for other methods?

Thank you for this question. For all competing methods, we followed the recommended default hyperparameter settings provided in their official documentation or tutorials. These defaults are those used by the original authors in their published benchmarks and example workflows, and are intended to represent standard, commonly used configurations. To avoid introducing unintended bias, we did not perform additional method-specific hyperparameter tuning beyond these recommended settings, consistent with common practice in large-scale benchmarking studies. We have added this information in the Methods section:

"*All the baseline methods are run with the recommended default hyperparameter settings provided in their official documentation or tutorials.*" (Page 25, Line 768-769)

5. Regarding functional divergence and convergence, only case studies are shown, and a systematic evaluation is missing.

We thank the reviewer for raising this important point. We completely agree that while case studies are illustrative, relying on them solely is insufficient to support broad claims about functional divergence and convergence. In response, we have developed and conducted a rigorous, systematic, and quantitative evaluation of these evolutionary dynamics, which is now included in the revised manuscript.

(1) Establishing an Independent Ground Truth

Because no curated, "gold-standard" datasets explicitly annotate functionally divergent or convergent gene pairs across species, we first established robust operational definitions. Crucially, these definitions rely on metrics *entirely independent* of the embeddings used by our model (ESM and LLaMA). Specifically, reciprocal best-hit (RBH) orthologs were identified by BLAST[10], where each gene is the best cross-species match of the other, and functional similarity was quantified using Gene Onotology (GO) Lin semantic similarity[11], an information-content-based measure derived from GO graph structure and annotation that ranges from 0 to 1.

- **Functional Divergence:** Defined as gene pairs sharing a common evolutionary origin but exhibiting reduced functional similarity. We operationalized this as reciprocal best-hit (RBH) orthologs with low GO Lin semantic similarity (≤ 0.3).
- **Functional Convergence:** Defined as evolutionarily distant gene pairs displaying high functional similarity. We operationalized this as non-RBH gene pairs lacking significant sequence homology (BLAST E-value > 0.05) but exhibiting high GO Lin similarity (≥ 0.7).

To ensure the highest quality annotations for this systematic evaluation, we focused this analysis on the human-mouse comparison, as these species possess the most comprehensive and reliable GO annotations.

(2) Macrogene Enrichment Analysis (Permutation Testing)

Having established this independent ground truth, we tested whether Unify's macrogenes systematically capture these complex relationships better than chance.

For each macrogene, genes were ranked according to their learned gene-to-macrogene assignment weights, and the top K genes in each species were defined as the core component genes. Ground-truth divergent and convergent cross-species gene pairs were then quantified

within these core gene sets. To evaluate whether the observed enrichment exceeded chance expectation, a null-model analysis with 1,000 permutations was performed, in which gene-to-macrogene assignments were randomized while preserving the number of genes assigned to each macrogene in each species.

- Results: This analysis revealed that Unify's macrogenes are significantly enriched for both functionally divergent and convergent gene pairs compared to the random null model (Supplementary Figure 26a-d). This provides systematic, statistical evidence that macrogenes are not merely grouping genes by superficial similarity, but instead capture biologically meaningful modules reflecting complex evolutionary histories.

(3) The Advantage of a Multi-Modal Approach (AUROC Analysis)

Finally, we investigated *why* Unify is able to capture these relationships by testing the predictive power of its individual modalities (ESM and LLaMA) against the established ground truth. We computed AUROC values to determine if structural (ESM) or text-based functional (LLaMA) similarity alone could distinguish the divergent/convergent pairs.

- Results: The results clearly demonstrated the advantage of our multi-modal approach (Supplementary Figure 26e).
    - For identifying **functional divergence** (orthologs with different functions), structural similarity (ESM) performed near random chance (AUROC ~0.57), whereas text-based functional similarity (LLaMA) was highly predictive (AUROC ~0.73). This confirms that protein structure alone is often blind to functional divergence.
    - Conversely, for identifying **functional convergence** (non-homologous genes with similar functions), functional text similarity (LLaMA) was a stronger predictor (AUROC ~0.70) than structural similarity (ESM) (AUROC ~0.65), suggesting that convergent gene pairs may be more consistently linked by shared functional semantics than by structural similarity alone.

Together, these new analyses provide a robust, systematic validation of functional divergence and convergence. They prove that Unify's multi-modal macrogenes integrate evolutionary and functional signals in a way that cannot be achieved by single modalities alone. We have added a dedicated sub-section to the Results and comprehensive details to the Methods section describing this systematic evaluation framework.

Changes to manuscript:

"To systematically assess whether Unify captures functional divergence and convergence, an annotation-supported human-mouse benchmark was constructed. Functionally divergent pairs were defined as reciprocal best-hit (RBH) orthologs, in which each gene is the best cross-species match of the other in BLAST, with low GO Lin semantic similarity, a measure of functional similarity derived from Gene Ontology (GO) structure. Functionally convergent pairs were defined as non-homologous gene pairs with high GO Lin semantic similarity (Methods). Under these definitions, Unify macrogenes were significantly enriched for both divergent and convergent gene pairs relative to permutation-based null models, indicating that the learned macrogenes capture biologically meaningful complex evolutionary relationships rather than random gene co-assignment. Consistent with this, the area under the receiver operating characteristic curve (AUROC) analysis showed that neither structural similarity nor functional text similarity alone was sufficient to recover both patterns, supporting the value of Unify's multimodal design (Supplementary Figure 26)." (Page 10, Line 285-296)

"Because no curated benchmark explicitly labels cross-species gene pairs as functionally divergent or convergent, these categories were defined using sequence relationships and GO-based functional similarity. To ensure high-quality annotation support, this analysis was restricted to the human–mouse comparison.

...

To assess the contribution of the individual modalities, structural similarity and functional text similarity were evaluated separately for their ability to distinguish divergent and convergent gene pairs. Area under the receiver operating characteristic curve (AUROC) values were computed through the function roc_auc_score in sklearn.metrics by using ESM-derived structural similarity and LLaMA-derived functional similarity against the benchmark definitions described above, allowing assessment of whether either modality alone was sufficient to recover these relationships." (Page 27, Line 836-862)

6. Figure 3E and 3F: There are several dotted lines that are not annotated. Also, it is unclear how the similarity score between ESM and LLaMA is calculated. Intuitively, since these two

embeddings are generated separately, a direct correlation analysis between them does not make sense due to the separate coordinates of these two embeddings.

Thank you for raising this important point. We have revised Figure 3e–f to improve clarity and address both issues raised.

First, we have added explicit legends and annotations to indicate that the dotted lines correspond to the identity line ($y = x$), which serves as a visual reference for comparing relative agreement between the two similarity measures.

Second, we clarify the interpretation of the similarity comparison between ESM and LLaMA embeddings. The x-axis represents the ESM-based protein embedding similarity between cross-species gene pairs, while the y-axis represents the LLaMA-based functional (text) embedding similarity for the same gene pairs. Because these embeddings are generated in different latent spaces, we do **not** interpret this analysis as a direct coordinate-wise correlation. Instead, each similarity score is computed within its own embedding space (using cosine similarity) and then independently normalized to the range [0, 1] using min–max scaling.

The purpose of this visualization is therefore comparative rather than correlational: it illustrates how structural similarity (ESM) and functional similarity (LLaMA) function at the level of gene pairs, highlighting cases of agreement (near the y=x dotted line) as well as systematic deviations that motivate the distinction between conserved, convergent, and divergent gene relationships. We have clarified this interpretation in the figure caption and the main text to avoid confusion.

These revisions improve the interpretability of Figure 3e–f and more clearly convey the intended biological insight rather than implying a direct embedding-space correlation.

We have now updated the method section to make it clear:

"*Structural similarity between genes was calculated as the cosine similarity between their ESM-derived protein embeddings, and functional similarity was calculated as the cosine similarity between their LLaMA-derived functional description embeddings. Because these two similarity distributions are derived from different embedding spaces, ESM- and LLaMA-based similarities were min–max scaled separately to [0,1] before comparison. This normalization enabled relative structural and functional similarity to be compared on a common scale.*" (Page 24, Line 747-752)

7. Regarding perturbation effect prediction, it is not clear how and why the linear transformation of macrogenes works. For example, for the upper right box in Figure 4a, I am guessing the addition and subtraction are done on the cell by macrogene space, but then why is the subtraction done when there is no cell correspondence across species and perturbation status?

Thank you for raising this important point. We apologize that the rationale behind the linear transformation was not sufficiently explained in the original manuscript.

Our perturbation transfer strategy follows the linear-manifold assumption introduced in scGen[12] for cross-species perturbation modeling. Specifically, we assume that the effect of a perturbation can be approximated as a direction, or shift vector, in the shared macrogene embedding space, and that this shift is sufficiently conserved across species after integration. Accordingly, the subtraction and addition operations are performed in the cell-by-macrogene embedding space, rather than between explicitly matched individual cells across species or perturbation conditions.

Specifically, we compute the perturbation effect in the reference species A as:

$$\Delta_A = \text{Embedding}(A_{\text{perturb}}) - \text{Embedding}(A_{\text{control}})$$

where each term represents the centroid (or average representation) of cells under that condition. This difference vector captures the global perturbation-induced transcriptional shift in the aligned macrogene space.

Under the assumption that homologous cell types respond to the same perturbation along comparable biological axes, we transfer this effect to species B by:

$$\hat{B}_{\text{perturb}} = \text{Embedding}(B_{\text{control}}) + \Delta_A$$

Thus, no one-to-one cell correspondence is required. The subtraction is performed between condition-level centroids within the same species, and the resulting perturbation vector is applied to the control centroid of the target species.

This approach is analogous to vector arithmetic commonly used in representation learning (e.g., latent space translation), where biological effects are modeled as approximately linear transformations in a shared embedding space.

We have clarified this assumption and computational procedure in the revised Methods section (Page 23, Line 700-704) to make the logic more explicit.

"*Our perturbation transfer strategy follows the linear-manifold assumption introduced in scGen for cross-species perturbation modeling. We assumed that perturbation effects can be represented as a direction (or shift vector) in the shared macrogene embedding space, and that this perturbation-induced shift is approximately conserved across species.*"

8. The authors claim "all genes" in the figure legend, but indicated that they tested on "highly variable genes" on Line 298 for those figures. It would be more practically useful if the experiments were truly done on all genes instead of highly variable genes.

Thank you for pointing out this inconsistency. We agree that the terminology in the original manuscript was unclear. In the revised version, we have corrected this issue by redefining the "All genes" setting to truly include all available genes in the dataset, rather than restricting the analysis to highly variable genes.

Accordingly, we have updated the main text description and Figure 4 to reflect this change and to ensure consistency between the text and the experiments. This revision makes the evaluation more comprehensive and better aligned with practical applications.

9. It is also unclear whether the difference between Unify and other methods is statistically significant.

Thank you for raising this important point. To address this concern, we performed additional pairwise statistical comparisons using paired two-sided t-tests across cell types/conditions, followed by Benjamini–Hochberg correction for multiple testing. These analyses were conducted for both Unify variants (Unify-basal and Unify-macrogene) against all competing methods, and the resulting significance annotations are now included in Figure 4b–h and described in the revised Results section.

In the **IFN-β perturbation task**, a more challenging task required cross-context extrapolation (in vivo mouse lymph node to in vitro human PBMCs), both Unify-basal and Unify-macrogene achieved significantly higher R^2 than the linear model and scGen in the one-to-one gene setting, and significantly higher R^2 than all competing methods in the all-gene setting (Figure 4b). For

DEG hit rate, Unify-basal performed significantly better than all baseline methods in both the one-to-one and all-gene settings (Figure 4c). For DEG direction accuracy, Unify-macrogene showed significantly better performance than competing methods (Figure 4d).

In the **LPS perturbation task**, which is a less complex task (involves matched in vitro data from the same cell type across species), both Unify variants remained competitive with scPARM in the one-to-one gene setting for R^2, while in the all-gene setting, they achieved significantly higher R^2 than all other tested baseline methods (Figure 4f). For both DEG hit rate and directional accuracy, the two Unify variants significantly outperformed the linear model and scGen in both gene settings. In addition, Unify-basal further showed significantly higher directional accuracy than scPARM in both gene settings (Figure 4g,h).

Together, these analyses show that the performance improvements of the Unify variants are statistically supported for the main evaluation metrics, particularly for transcriptome-level recovery in the all-gene setting and for DEG-level prediction accuracy.

Changes to the manuscript:

To make this explicitly clear to the readers, we have expanded the main text in the Results section:

"*When restricted to predicting changes based on one-to-one orthologs alone, Unify variants (Unify-basal and Unify-macrogene) performed comparably to scPARM, a state-of-the-art method specifically developed for perturbation effect prediction (Figure 4b, Supplementary Table 11). However, the advantage of Unify framework becomes more evident when the predictions were expanded to the full gene set. By predicting across all genes, which capture the most dynamic aspects of the cellular response, both Unify variants achieved significantly higher accuracy (R^2) in predicting the final expression state compared to all baseline methods and showed strong performance in DEG hit rate and directional accuracy (Figure 4b-e). The robustness of this framework was further assessed by performing additional zero-shot cross-species predictions, using data from lipopolysaccharide (LPS) stimulated mouse macrophages to predict the responses in rat, rabbit, and pig macrophages. Across these transfers, both Unify variants again achieved significantly higher R^2 in the all-gene setting and remained highly competitive with scPARM in DEGs hit rate and directional accuracy (Figure 4f-h, Supplementary Table 12).*" (Page 13, Line 375-387)

We have also updated the Methods section to reflect the new statistical tests:

"*Statistical comparisons of method performance in perturbation prediction were performed in R (stats package) using paired two-sided t-tests across cell types/conditions, comparing Unify-macrogene and Unify-basal against each other and all competing methods. Resulting p-values were adjusted for multiple testing using the Benjamini-Hochberg procedure*." (Page 24, Line 742-746)

10. Similarly, regarding cell type tree construction, it is not surprising that Unify performs well, given that it has already seen the cell type mapping across species. It would be useful if Unify can identify some new connections that are not indicated in the original mapping.

We appreciate the reviewer's thoughtful comment and agree that, because Unify incorporates cell-type information during training, it is important to clarify why strong performance in downstream cell-type tree construction cannot be attributed simply to having seen known mappings.

(1) Cell-type tree construction is performed post hoc and remains fully unsupervised

The cell-type tree is not learned directly during model training, but is constructed post hoc from the learned embedding space using PHYLIP (contml). PHYLIP infers a maximum-likelihood tree under a Brownian motion model of continuous trait evolution, treating each species-cell-type representation as a vector of continuous characters (derived from the top principal components of the embedding). Importantly, PHYLIP has no access to cell-type labels, species identities, or known correspondences during tree inference. Thus, the resulting topology emerges entirely from the learned latent structure rather than from supervised labels themselves.

(2) Cell-type supervision acts only as a local alignment prior

Although Unify uses cell-type labels during training, this supervision serves only as a soft alignment prior rather than a rigid alignment rule. It encourages corresponding cell populations to occupy nearby regions in latent space but does not enforce exact overlap, constrain the relative distances among distinct cell types, or prescribe higher-order relationships across all species. Consequently, the global branching structure of the tree remains an emergent property of the learned embedding.

This is illustrated by the case of *Schmidtea mediterranea*. Despite the use of supervised alignment during training, both Unify and SATURN recover a strongly species-specific clade for Schmidtea cell types. Although some Schmidtea cell types, such as muscle cells, share broad lineage relationships with those in other species, they are not simply merged with their annotated counterparts across species. Instead, they remain grouped within a distinct species-specific branch. This observation further supports that the inferred topology is not trivially dictated by the input labels, since strong species-level transcriptional signals can still dominate when biologically appropriate.

(3) Unify reveals biologically meaningful higher-order relationships beyond known mappings

Beyond recovering expected correspondences, Unify identifies global relationships that are more consistent with known evolutionary conservation.

For example, neuronal populations from multiple species form a tighter shared clade in the Unify-derived tree, whereas in the SATURN tree they separate into multiple sub-branches. Given the strong evolutionary conservation of neuronal regulatory programs, this pattern suggests that Unify better preserves shared functional relationships across species.

A similar pattern is observed for epithelial lineages. In the SATURN tree, epithelial cells from different species are relatively dispersed, whereas in the Unify tree, they form a coherent branch whose internal branching order broadly follows expected phylogenetic relationships: mammals (*Homo sapiens* and *Mus musculus*) cluster as sister groups, followed by teleost fish (*Danio rerio*), and then invertebrates, including *Drosophila melanogaster* and *Caenorhabditis elegans*. This organization is consistent with the interpretation that core epithelial regulatory programs were established early during metazoan evolution and subsequently maintained through largely vertical inheritance, while still allowing lineage-specific diversification.

(4) Potential to reveal relationships beyond predefined mappings

Because Unify learns embeddings through multimodal macrogene representations rather than relying solely on pairwise annotation transfer, the latent space integrates broader functional relationships across genes and species. As a result, the inferred tree is not merely a recapitulation

of original training labels, but can reveal biologically meaningful higher-order connections that are not explicitly encoded in the predefined mappings.

We have clarified these points in the revised manuscript.

"*In addition to recovering expected broad evolutionary relationships, Unify also revealed finer-grained higher-order connections among conserved cell lineages that were less clearly resolved by SATURN. For example, neuronal populations from multiple species formed a tighter shared clade in the Unify-derived tree, consistent with the deep conservation of neuronal regulatory programs across metazoans. Similarly, epithelial cell types from vertebrates and invertebrates formed a coherent branch whose internal ordering broadly reflected known phylogenetic relationships, with mammals (Homo sapiens and Mus musculus) clustering as sister groups, followed by teleost fish (Danio rerio), and then invertebrates including Drosophila melanogaster and Caenorhabditis elegans. This pattern is consistent with the possibility that core epithelial regulatory programs were established early in animal evolution and have remained relatively stable despite extensive lineage diversification.*" (Page 15, Line 437-447)

11. Ablation study on whether adding functional embeddings or structural embeddings improves the performance is needed.

Thank you for raising this important point about the need to incorporate functional embeddings and structural embeddings. In response, we performed a dedicated ablation study to explicitly quantify the contribution of multimodality in Unify.

We compared three model variants across multiple cross-species integration tasks spanning different evolutionary distances:

- Unify (Full), incorporating both protein-based (ESM) and text-based (LLaMA) embeddings.
- Unify (ESM-only), using only protein embeddings.
- Unify (LLaMA-only), using only text embeddings.

Across all evaluated tasks, Unify (Full) achieved higher biological conservation and overall integration scores than either unimodal variant. These results are now reported in Supplementary Table 5 and Supplementary Figure 20. Notably, the unimodal variants exhibit complementary strengths: protein embeddings alone better preserve structural similarity, while text embeddings

alone capture higher-level functional semantics but lack sequence-level resolution. Their combination in Unify enables a more comprehensive representation that jointly captures structural and functional signals, thereby improving cross-species alignment and biological conservation.

Together, these results demonstrate that the inclusion of text-based embeddings is not redundant but instead provides a significant performance gain when integrated with protein embeddings, supporting the necessity of the multimodal macrogene design.

We have updated the Results section to contain this new ablation study:

"*Across ten integration tasks spanning cross-genus to cross-phylum comparisons, the full multimodal model, initialized with both ESM-derived protein embeddings and LLaMA-derived functional text embeddings, outperformed the single-modality variants. The full model achieved significantly higher bio-conservation and overall scores (Supplementary Figure 20, Supplementary Table 5). This indicates that structural and functional priors provide complementary information for macrogene construction, enabling the latent space to better preserve shared cellular programs across vast evolutionary distances.*" (Page 7, Line 216-222)

We also included the relevant description in the Methods section:

"*For the multimodality ablation, three model variants were compared: Unify, using both ESM-derived protein embeddings and LLaMA-derived functional text embeddings; Unify (ESM-only), using only protein embeddings; and Unify (LLaMA-only), using only text embeddings. ... In all ablation analyses, model parameters and training settings were kept identical to those of the Unify model, and only the component under investigation was modified. Performance was assessed using the same integration metrics as in the main benchmark.*" (Page 25, Line 782-804)

Response to Reviewer 3:

In this manuscript, Zhong et al. present Unify, a deep learning framework designed for integrating single-cell RNA-seq data across species. The authors introduce universal multimodal embeddings that incorporate gene expression, structural features, and functional annotations derived from LLMs. They also employ MGDA-UB to automatically correct batch effects with biological conservation, eliminating the need for manual weight adjustment. The study shows that Unify outperforms existing methods across various evolutionary distances, particularly excelling in comparisons between distant species where other methods struggle. Furthermore, the authors applied Unify to predict cellular responses to perturbations across species. However, several major and minor concerns need to be addressed.

We sincerely thank the reviewer for the careful reading of our manuscript and for the thoughtful summary of our work. We appreciate the reviewer's recognition of the novelty of Unify, including its use of universal multimodal embeddings and Pareto-optimal multi-task learning to balance batch correction and biological conservation without manual loss weighting. We are also encouraged that the reviewer recognized the broad scope of our evaluation across evolutionary distances and the potential of Unify for cross-species perturbation prediction. We are grateful for the reviewer's constructive comments, which have helped us improve the clarity and rigor of the manuscript. In response, we have carried out additional analyses and revised the manuscript accordingly. Below, we provide a detailed point-by-point response to each comment.

Major concerns:

1. In the "Cell type tree construction" section, the strategy of randomly sampling "one cell per cell type per species" is mentioned. Relying on a single cell to represent an entire cell type for phylogenetic analysis is statistically problematic given the sparsity and noise inherent in scRNA-seq data (e.g., dropouts). Please perform a sensitivity analysis by increasing the number of sampled cells (e.g., sample 10, 50, 100 cells per type and calculate a consensus) or by using "pseudobulks/centroids" to represent cell types. Demonstrate that the tree topology is consistent under these more robust representations would significantly strengthen the biological validity of your conclusions regarding cell type evolution.

Thank you for raising this important concern. We also appreciate the opportunity to clarify and correct an imprecise statement in the original manuscript. After careful re-examination, we would

like to clarify that our cell type tree construction does **not** rely on randomly sampling a single cell per species-cell-type group.

Instead, for each species-cell-type, we compute a robust centroid representation in the learned embedding space. Specifically, we perform within-group k-means clustering and compute a meta-centroid by averaging the cluster centroids. We then select the cell closest to this meta-centroid as a representative ("centroid cell") for tree construction. This approach mitigates the impact of scRNA-seq sparsity and dropout by capturing the central tendency of each cell-type population rather than relying on an arbitrary single cell.

In addition, we constructed an alternative tree by averaging the species-cell-type representations and compared it with the centroid-cell-based tree using the weighted Robinson–Foulds (wRF) distance[13]. The low wRF value (0.28) indicates strong topological similarity between the two trees, suggesting that the inferred evolutionary relationships are robust to the choice of cell-type representation. Together, these results suggest that the inferred evolutionary relationships are robust to the representation strategy used for each cell type and are not highly sensitive to whether cell types are represented by centroid cells or averaged embeddings.

We have corrected the description in the manuscript accordingly and expanded the Methods section to clearly describe the centroid-based strategy and accompanying sensitivity analyses.

"*To reduce computational complexity, one cell per cell type per species was selected. Specifically, for each species-cell-type, cells were clustered by k-means in the learned embedding space, and the resulting cluster centroids were averaged to obtain a meta-centroid. The cell nearest to this meta-centroid was selected as the representative cell ("centroid cell") for tree construction.*" (Page 28, Line 870-874)

"*To assess robustness to representation choice, an alternative tree was constructed directly by averaging the cell embeddings within each species-cell-type and compared with the centroid-cell-based tree using the weighted Robinson–Foulds distance (Supplementary Figure 38, Supplementary Note 4).*" (Page 28, Line 878-881)

2. In the "Benchmark evaluations", it is noted that Unify uses all genes, whereas baseline methods (such as scGen or scVI) are limited to one-to-one orthologs. Although leveraging all genes is a methodological advantage of Unify, it introduces a confusion in the evaluation: does the observed

performance gain come from the Unify architecture itself or simply from having access to more input features?

Thank you for raising this important point. We agree that because Unify can leverage all genes, whereas several baseline methods are restricted to one-to-one orthologs, it is important to explicitly distinguish whether the observed performance gain arises from the model architecture itself or simply from expanded feature coverage.

To address this, we performed an explicit ablation study comparing Unify, which uses all available genes, with Unify (one-to-one), in which the input gene set is restricted to exactly the same one-to-one orthologs used by baseline methods such as scVI and scGen (Supplementary Table 9, Supplementary Figure 24). Both variants were evaluated alongside the baseline methods across multiple cross-species integration tasks under identical benchmarking settings, allowing us to directly isolate the effect of feature coverage from that of model design.

Across these evaluations, Unify (one-to-one) consistently outperformed the baseline methods, demonstrating that Unify's advantage is not solely attributable to access to additional genes. This indicates that the core architectural design of Unify already confers a substantial benefit even when all methods are compared within the same input feature space.

At the same time, Unify and Unify (one-to-one) achieved highly comparable overall performance across most cross-species categories, with no statistically significant difference at the global level. The benefit of using all genes became more apparent, mainly in the most challenging settings, particularly cross-phylum integration, where additional gene coverage is expected to provide functional information that cannot be captured by strict one-to-one orthologs alone.

Taken together, these results indicate that the primary source of performance improvement arises from Unify's architecture, including its multimodal macrogene representation and Pareto-optimized integration strategy, while expanded gene coverage provides an additional advantage mainly under extreme evolutionary divergence.

We have added these ablation analyses to the revised manuscript:

"*Finally, restricting the input to one-to-one orthologs (Unify (one-to-one)) reduced performance relative to the full model using all genes, but Unify (one-to-one) still significantly outperformed the baseline methods (Supplementary Figure 24, Supplementary Table 9). This underscores that the*

Unify framework remains effective and robust even under a restricted gene space." (Page 8, Line 244-247)

We also included the relevant description in the Methods section:

"*For the input-gene-space ablation, Unify, which uses all available genes as input, was compared with Unify(one-to-one), in which the input was restricted to one-to-one orthologs. ... In all ablation analyses, model parameters and training settings were kept identical to those of the Unify model, and only the component under investigation was modified. Performance was assessed using the same integration metrics as in the main benchmark."* (Page 26, Line 797-804)

3. The paper claims significant benefits from ESM-2 and LLaMA-2 embeddings. To quantify this benefit, an ablation study is necessary. Please report Unify's performance (e.g., bio-conservation scores) when: (a) Only sequence homology/structural embeddings are used; (b) Only LLM-derived functional text embeddings are used.

Thank you for raising this important point about the need to incorporate functional embeddings and structural embeddings. In response, we performed a dedicated ablation study to explicitly quantify the contribution of multimodality in Unify.

We compared three model variants across multiple cross-species integration tasks spanning different evolutionary distances:

- Unify (Full), incorporating both protein-based (ESM) and text-based (LLaMA) embeddings.
- Unify (ESM-only), using only protein embeddings.
- Unify (LLaMA-only), using only text embeddings.

Across all evaluated tasks, Unify (Full) achieved higher biological conservation and overall integration scores than either unimodal variant. These results are now reported in Supplementary Table 5 and Supplementary Figure 20. Notably, the unimodal variants exhibit complementary strengths: protein embeddings alone better preserve structural similarity, while text embeddings alone capture higher-level functional semantics but lack sequence-level resolution. Their combination in Unify enables a more comprehensive representation that jointly captures structural and functional signals, thereby improving cross-species alignment and biological conservation.

Together, these results demonstrate that the inclusion of text-based embeddings is not redundant but instead provides a significant performance gain when integrated with protein embeddings, supporting the necessity of the multimodal macrogene design.

We have updated the Results section to contain this new ablation study:

"*Across ten integration tasks spanning cross-genus to cross-phylum comparisons, the full multimodal model, initialized with both ESM-derived protein embeddings and LLaMA-derived functional text embeddings, outperformed the single-modality variants. The full model achieved significantly higher bio-conservation and overall scores (Supplementary Figure 20, Supplementary Table 5). This indicates that structural and functional priors provide complementary information for macrogene construction, enabling the latent space to better preserve shared cellular programs across vast evolutionary distances.*" (Page 7, Line 216-222)

We also included the relevant description in the Methods section:

"*For the multimodality ablation, three model variants were compared: Unify, using both ESM-derived protein embeddings and LLaMA-derived functional text embeddings; Unify (ESM-only), using only protein embeddings; and Unify (LLaMA-only), using only text embeddings. ... In all ablation analyses, model parameters and training settings were kept identical to those of the Unify model, and only the component under investigation was modified. Performance was assessed using the same integration metrics as in the main benchmark.*" (Page 25, Line 782-804)

Minor

1. The framework incorporates complex components, such as LLMs and adversarial training with MGDA-UB. Please provide a table comparing the runtime and peak memory usage (GPU VRAM) of Unify versus major competitors (e.g., SATURN and SAMap) on datasets of varying sizes. This will useful to assess the practical accessibility of the tool.

Thank you for this insightful suggestion. We agree that reporting computational requirements is important for evaluating the practical applicability of a new integration framework, particularly given the use of foundation-model-derived embeddings and adversarial multi-objective training in Unify. We would like to clarify that, since we use the pretrained ESM/LLaMa embeddings, the

cost of feature extraction is fixed; however, the overall computational cost during training and inference still scales with dataset size.

In response, we performed an additional computational benchmark using a representative large-scale integration task between *Danio rerio* and *Strongylocentrotus purpuratus*, which represents a challenging scenario due to the substantial imbalance in dataset size (approximately 1 million cells versus 60,000 cells). To systematically assess scalability, we subsampled the zebrafish dataset at 1%, 6%, 20%, 40%, 60%, 80%, and 100%, generating datasets ranging from approximately 70,000 to 1.1 million cells.

For each method, we recorded hardware configuration, memory usage, and runtime across all dataset sizes. These results are now summarized in Supplementary Table 4 and Supplementary Figure 19, including CPU/GPU configuration, memory consumption, and runtime statistics.

The analysis shows that lightweight methods such as Harmony, scVI, and Scanorama remain highly efficient and complete integration within one hour even at a million-cell scale. In contrast, methods such as Seurat and graph-based approaches, including SAMap show substantial runtime increases as dataset size grows.

Unify demonstrated favorable scalability across all tested dataset sizes. Runtime increased smoothly from medium-scale datasets to approximately 1–5 hours at 1.1 million cells, remaining consistently below SATURN (which is the deep learning-based method most similar to ours) and substantially below Seurat v4 CCA at large scale. Although slower than lightweight methods such as Harmony and fastMNN, Unify maintained a practical computational profile while delivering stronger biological integration performance.

We have further revised the manuscript to contain this assessment:

"*Beyond its strong integration performance, Unify also showed favorable computational scalability across datasets ranging from 70K to 1.1M cells. It scaled more favorably than several competing cross-species integration approaches, including SATURN, SAMap, Seurat v4 CCA, and BBKNN, particularly at larger dataset sizes (Supplementary Figure 19, Supplementary Table 4). This indicates that Unify combines strong integration accuracy with practical scalability for large comparative atlases.*" (Page 7, Line 204-209)

2. The manuscript reports predicting perturbation responses (e.g., Mouse to Human) and mentions validation against "experimentally measured data". Please explicitly describe the data-splitting strategy. Was the target species' perturbed data completely excluded during the training phase (zero-shot prediction)? Clarification is needed to ensure that no data leakage occurred, as this is a common concern in generative integration tasks.

Thank you for raising this important clarification regarding the perturbation prediction setup. We apologize for the lack of explicit descriptions in the original manuscript.

In all cross-species perturbation prediction experiments (e.g., Mouse → Human), the perturbed data from the target species is completely excluded during model training. Specifically, Unify is trained only on:

(i) control (unperturbed) cells from the target species, and

(ii) both control and perturbed cells from the source species.

The target species' perturbed condition is held out entirely and used only for evaluation, ensuring that the task constitutes a strict zero-shot prediction with no exposure to target-species perturbation signals during training. This design prevents any form of data leakage and aligns with standard practices in generative cross-domain evaluation.

We have now revised the main text to explicitly describe this data-splitting strategy and to clearly state the zero-shot nature of the perturbation prediction experiments.

"*...This prediction was performed in a zero-shot setting, where the target species perturbation condition was entirely excluded during training and inferred solely from perturbation representations learned from the source species...*" (Page 13, Line 372-375)

3. In line 112, the text mentioned 'Figure 1A'. However, Figure 1 does not include panel A. Please carefully review and correct the main text and figures.

Thank you for pointing this out. We apologize for the inconsistency. We have carefully checked the manuscript and corrected the reference from "Figure 1A" to "Figure 1" (and verified that all figure/panel citations throughout the main text now correctly match the updated figures and captions).

4. In Figure 2A, please include a legend explaining the meaning of the dark and light colors.

Thank you for this helpful suggestion. We have revised Figure 2a to include a clear legend explaining the meaning of the dark and light colors to improve clarity and readability.

5. In line 408, the characters are not displayed correctly.

Thank you for bringing this formatting issue to our attention. We have corrected the notation in the original line 408 to ensure that it is properly rendered and visible in the PDF version of the manuscript. We apologize for the inconvenience caused by this display error.

6. The authors refer to a GitHub repository. Please ensure that it includes a “minimal reproducible example” (a toy dataset) accompanied with a clear tutorial. This will enable reviewers and users to quickly verify installation and basic runs without downloading large datasets.

Thank you for this helpful suggestion. In response, we have added a lightweight toy dataset and a step-by-step tutorial to the GitHub repository.

Reference

1. Rosen, Y. et al. Toward universal cell embeddings: integrating single-cell RNA-seq datasets across species with SATURN. *Nature Methods* **21**, 1492-1500 (2024).
2. Rosen, Y. et al. Universal cell embeddings: A foundation model for cell biology. *bioRxiv*, 2023.2011. 2028.568918 (2023).
3. Rives, A. et al. Biological structure and function emerge from scaling unsupervised learning to 250 million protein sequences. *Proceedings of the National Academy of Sciences* **118**, e2016239118 (2021).
4. Heinzinger, M. et al. Bilingual language model for protein sequence and structure. *NAR Genomics and Bioinformatics* **6** (2024).
5. Ahmed, E. et al. Prottrans: towards cracking the language of life’s code through self-supervised deep learning and high performance computing. *BioRxiv* (2020).
6. Touvron, H. et al. Llama 2: Open foundation and fine-tuned chat models. *arXiv preprint arXiv:2307.09288* (2023).
7. Gu, Y. et al. Domain-specific language model pretraining for biomedical natural language processing. *ACM Transactions on Computing for Healthcare (HEALTH)* **3**, 1-23 (2021).
8. Workshop, B. et al. Bloom: A 176b-parameter open-access multilingual language model. *arXiv preprint arXiv:2211.05100* (2022).
9. Luecken, M.D. et al. Benchmarking atlas-level data integration in single-cell genomics. *Nature methods* **19**, 41-50 (2022).
10. Camacho, C. et al. BLAST+: architecture and applications. *BMC bioinformatics* **10**, 421 (2009).
11. Wang, J.Z., Du, Z., Payattakool, R., Yu, P.S. & Chen, C.-F. A new method to measure the semantic similarity of GO terms. *Bioinformatics* **23**, 1274-1281 (2007).
12. Lotfollahi, M., Wolf, F.A. & Theis, F.J. scGen predicts single-cell perturbation responses. *Nature methods* **16**, 715-721 (2019).
13. Robinson, D.F. & Foulds, L.R. Comparison of phylogenetic trees. *Mathematical biosciences* **53**, 131-147 (1981).

Table of Contents

Overview: **.. 1**
Response to Reviewer #1: **... 2**
Response to Reviewer #2: **... 3**
Response to Reviewer #3: **... 7**

Overview:

We sincerely thank all the reviewers for their continued engagement with our work. In this second round of revision, we have carefully addressed the remaining comments from Reviewer #1 and Reviewer #2. Reviewer #3 has confirmed that all previous concerns have been resolved.

The following summarizes the key revisions made in response to the second-round comments:

1. Corrections and clarifications requested by Reviewer #1 (minor points).

- Corrected the scientific name from *Macaque mulatta* to *Macaca mulatta* throughout the manuscript.
- Standardized all citations of scGen to correctly point to the original scGen paper (reference 26).
- Fixed the typo “SATRUN” to “SATURN” in the Methods section.

2. Methodological clarifications and updated benchmarking requested by Reviewer #2.

- Explicitly listed all tuned hyperparameters of the full Unify model (batch size, latent size, gradient normalization type, adversarial network hidden sizes, learning rates, dropout ratios, and training epochs) in the Methods section.
- Clarified that ablation models use identical hyperparameters to isolate component contributions.
- Standardized the comparison protocol: Unify now uses its fixed default hyperparameters, while competing methods use their published defaults. The benchmark results have been updated accordingly, and Unify continues to outperform baseline methods in both batch correction and bio-conservation scores.

- Revised the Methods section to ensure full transparency regarding hyperparameter tuning fairness and model robustness.

All changes in the revised manuscript have been marked using red text. In this response, we marked the comments from reviewers in black and our responses in blue. We believe these revisions have strengthened the manuscript and addressed the remaining concerns raised by the reviewers. We hope the revised manuscript is now suitable for publication in *Nature Communications*.

==

Response to Reviewer #1:

Comments: *The authors have been thorough in their revisions, and I appreciate the additional analyses provided. The scientific concerns I previously raised have been largely resolved, and the model's robustness is now much more transparent. The study is ready for publication, provided the following minor points are addressed:*

We sincerely thank the reviewer for their positive assessment and for the additional minor suggestions. Below is our point-by-point response. We have revised the manuscript accordingly.

1. A correction is needed for the scientific name. The manuscript uses Macaque mulatta, which should be Macaca mulatta.

We thank the Reviewer for catching this oversight. We have corrected "*Macaque mulatta*" to "*Macaca mulatta*" throughout the manuscript (Main text, Methods, and Supplementary Information).

2. The citation for scGen needs to be standardized. It is currently linked to reference 17 (the transfer VAE paper) in some sections, while correctly attributed to reference 26 elsewhere. Please ensure all instances point to the correct source.

Thank you for this careful observation. We have reviewed the entire manuscript and standardized all citations of scGen to correctly point to reference 26 (the original scGen paper). The incorrect citations linking to reference 17 have been removed.

3. There is a persistent typo in the Methods section where SATURN is written as SATRUN. Please correct this.

We apologize for this typographical error. We have corrected “SATRUN” to “SATURN” in the Methods section. The manuscript has been double-checked for any additional typographical errors.

Response to Reviewer #2:

Comments: *Thank you for addressing many of the previous comments. I have one additional comment regarding the responses:*

In Line 802, the authors state that “In all ablation analyses, model parameters and training settings were kept identical to those of the Unify model, and only the component under investigation was modified.” It would be helpful for the authors to clarify which model parameters and training settings were tuned. In addition, could the authors comment on how the results might change if the ablation models were independently tuned, rather than using the identical settings from the full Unify model?

This is also related to my original question regarding hyperparameter tuning for competing methods. In practice, users would likely either tune hyperparameters for all methods or use default settings for all methods. Therefore, if Unify benefits from tuned hyperparameters, a fair comparison would require a comparable level of hyperparameter tuning for the competing methods as well. Clarifying this point would help support the claimed robustness, generalizability, and performance advantages of the proposed method on new datasets.

We sincerely thank the reviewer for recognizing our previous revisions and for raising this important point regarding benchmarking fairness and ablation study design.

We agree that a fair comparison requires a consistent and transparent hyperparameter strategy. In the original submission, Unify was evaluated using task-specific hyperparameters for the integration benchmarks, whereas the competing methods were run using their author-recommended or default pipelines.

We recognize that this asymmetric tuning strategy could complicate the interpretation of the benchmarking results and make it difficult to assess the generalizability of Unify under a standard-use setting. We agree that extensive hyperparameter optimization across all methods represents another possible benchmarking strategy. However, because each method has a distinct architecture and parameter space, performing a comprehensive and equally thorough optimization for every method would require a substantial additional study, while still making it difficult to ensure a comparable optimization effort across approaches. Instead, we adopted a standardized evaluation framework in which competing methods were run using their recommended settings, while Unify was evaluated using a single fixed configuration across all datasets. We believe this provides a practical and transparent assessment of performance under conditions that more closely reflect how these methods are typically applied to new datasets.

We would also like to clarify that Unify is evaluated in two settings in this study: cross-species integration and cross-species prediction. In the previous revision, the prediction experiments were already conducted using a unified hyperparameter configuration across all prediction tasks. The reviewer's concern therefore primarily applied to the integration benchmarks, for which the previous version used task-specific Unify hyperparameters. To address this concern in the current revision, we re-evaluated all integration benchmarks using a single universal Unify configuration across all integration tasks, without dataset-specific hyperparameter tuning.

1. Hyperparameter Tuning for Unify and Competing Methods

To address this concern and assess the robustness of the Unify architecture, we have completely updated our benchmarking evaluation. We discarded all task-specific tuning. Instead, we established a single, universal set of default hyperparameters for Unify, listed below:

Batch size: 256

Latent embedding size: 256

Gradient normalization type: l2

Hidden size for species discriminator: 256

Hidden size for cell type discriminator: 256

Learning rate for cell type discriminator: 0.0002

Learning rate for species discriminator: 0.0001

Learning rate for encoder: 0.0002

learning rate for generator: 0.0001

Decoder dropout ratio: 0.2

Decoder hidden size: 256

Encoder dropout ratio: 0.2

Encoder hidden size: 256

Train epochs: 500

We have revised the Methods section to explicitly list the specific parameters and training settings that were tuned for the full Unify model.

Furthermore, to ensure the ablation studies isolate the architectural components under investigation, all ablation models were also run using this identical, universal default configuration. We intentionally did not independently tune the ablation models, as doing so would confound the results; performance differences would become a mix of missing biological priors and hyperparameter artifacts. We have updated Figure 2, Supplementary Figure 1-18, 20-24, Supplementary Table 3 and 5-9 accordingly.

Even when restricted to a single universal default setting, Unify maintains its significant performance advantage over most baseline methods across the integration tasks. In addition, the conclusions from the ablation studies remain consistent with those reported in the previous version, further supporting the robustness of the proposed architecture (see updated Figure 2, Supplementary Figures 1-18, and 20-24).

We attribute this robustness primarily to Unify's core design:

(1) Multimodal Macrogenes: The biologically grounded priors provided by the combined ESM and LLaMA embeddings reduce the reliance on network fine-tuning to find meaningful latent structures.
(2) Pareto-Optimal (MGDA-UB) Training: In standard integration models, the most difficult and bias-prone aspect of tuning is finding the correct scalar weights to balance reconstruction, batch mixing, and bio-conservation. Unify's MGDA-UB optimization handles this trade-off dynamically and automatically at every training step, which reduces the largest source of hyperparameter dependency.

We appreciate the reviewer's suggestion, as it prompted a more rigorous evaluation of Unify under standardized conditions. The revised benchmark provides stronger and more transparent evidence for the robustness and generalizability of the method.

2. Clarification on the Ablation Studies

In machine learning, standard ablation practice dictates holding all hyperparameters constant to isolate the effect of the specific architectural component being removed (e.g., the LLaMA embedding). If we had independently tuned each ablation model, any observed performance difference could be confounded by the hyperparameter changes rather than the missing modality itself.

If the ablation variants (e.g., Unify-ESM only) were independently tuned, it is possible their absolute performance metrics might marginally improve on one specific task as the network adjusts to the restricted input space. However, this independent tuning would not be expected to substantially alter the relative ordering of the methods. The performance gap between single-modality and multimodal Unify is likely driven primarily by differences in the biological information available to the model (e.g., structural embeddings cannot capture purely functional convergence), which cannot be fully compensated for through hyperparameter tuning.

We have updated the Methods section accordingly (page 26, lines 802-813):

"Hyperparameter Configuration and Benchmarking Fairness

To ensure a fair and rigorous evaluation, we benchmarked Unify and all competing methods using consistent, default hyperparameter strategies, explicitly avoiding dataset-specific fine-tuning.

For all baseline methods (e.g., SATURN, scGen, SAMap, scVI), we utilized the default parameters, standard preprocessing pipelines, and recommended best practices provided in their official software implementations.

For Unify, we established a single, universal set of default hyperparameters that was applied rigidly across all integration tasks. This global configuration is available in Supplementary Table 15.

Finally, in all ablation studies, these exact network hyperparameters were held strictly constant. This ensures that any observed performance degradation in an ablated model (e.g., Unify-ESM only) is directly attributable to the removal of specific biological priors or architectural components, rather than confounding tuning artifacts."

Response to Reviewer #3:

Comments: *Overall, authors have addressed all my concerns.*

We thank the Reviewer for the positive assessment and for the previous constructive comments that helped improve the manuscript.
