# Supplementary material for: Unify learns cellular evolution with universal multimodal embeddings
Source: Nat Commun. 2026 Jul 31;17:9290. doi: 10.1038/s41467-026-76230-y (PMC13527131; doi:10.1038/s41467-026-76230-y)
Supplement: Supplementary file 4 [file 41467_2026_76230_MOESM3_ESM.pdf]

Description of Additional Supplementary Files

File Name: Supplementary Data 1
Description: Summary of cross-species integration methods

File Name: Supplementary Data 2
Description: Details of the datasets used in this study

File Name: Supplementary Data 3
Description: Details of the benchmark results for each task

File Name: Supplementary Data 4
Description: Runtime and computational resource usage of benchmarked methods across datasets of increasing cell numbers

File Name: Supplementary Data 5
Description: Detailed benchmark result of Unify variants using ESM embeddings only, LLaMA embeddings only, or combined ESM+LLaMA embeddings across cross-species integration tasks

File Name: Supplementary Data 6
Description: Detailed performance of Unify ablation variants using MGDA-UB or fixed loss-weighting strategies across cross-species integration tasks

File Name: Supplementary Data 7
Description: Detailed performance of Unify ablation variants using different protein language models and large language models across cross-species integration tasks

File Name: Supplementary Data 8
Description: Detailed performance of Unify ablation variants with partial annotation supervision and without the cell type classifier across cross-species integration tasks

File Name: Supplementary Data 9
Description: Detailed benchmark comparison of the Unify variant restricted to one-to-one orthologs across cross-species integration tasks

File Name: Supplementary Data 10
Description: Enriched GO terms for the component genes in macrogene 2077

File Name: Supplementary Data 11
Description: Details results of the IFN-beta cytokine perturbation prediction across different cell types for human using mouse dataset as reference

File Name: Supplementary Data 12
Description: Details results of the LPS perturbation prediction from mouse to pig, rat and rabbit

File Name: Supplementary Data 13
Description: Protein sequences files used by each dataset used in this study

File Name: Supplementary Data 14
Description: Evaluation tasks used in the ablation studies

File Name: Supplementary Data 15
Description: Default hyperparameters used in Unify
